# Synthesis and evaluation of the antimicrobial activity of novel esters, β-diketones, chromones and pyrazoles

**DOI:** 10.1039/d6ra07223b

**Published:** 2026-10-07

**Authors:** A. S. Shinde, S. P. Badadhe, S. P. Kunde, D. N. Gaikwad, B. S. Pawar, P. V. Badadhe

**Affiliations:** a P. G. Department of Chemistry and Research Centre, R. B. Narayanrao Borawake College Shrirampur M.S. India 413709 pvbadadhe@gmail.com; b P. G. Department of Chemistry and Research Centre, S. M. Joshi College Hadapsar M.S. India 411028; c Department of Microbiology, Yashavantrao Chavan Institute of Science, A Lead College of Karmveer Bhaurao Patil University Satara M.S. India 415001

## Abstract

In the present investigation, a new series of 2-acetylphenyl 6-chloropyridine-2-carboxylate derivatives were synthesised as the key intermediates and converted into 1,3-diketone derivatives *via* the Baker–Venkataraman rearrangement, which played a crucial role in the construction of the target chromone and pyrazole frameworks. 1-(6-Chloropyridin-2-yl)-3-(2-hydroxyphenyl)propane-1,3-diones, 2-(6-chloropyridin-2-yl)-4*H*-chromen-4-ones and 2-(5-(6-chloropyridin-2-yl)-1*H*-pyrazol-3-yl)phenols were synthesised. ^1^H-NMR, ^13^C-NMR and HRMS techniques were used to identify the structures of the synthesized compounds. The *in vitro* antimicrobial activity of the synthesized compounds was evaluated against three bacterial strains, one yeast and two filamentous fungi using the agar well diffusion method. The synthesized derivatives exhibited variable and organism-dependent antimicrobial activity, with selected compounds showing appreciable inhibition against the tested microorganisms. These results demonstrate the promising antibacterial activity of the synthesized compounds and encourage additional cytotoxicity and quantitative susceptibility testing to assess their biological potential.

## Abstract 2

A new series of 2-acetylphenyl 6-chloropyridine-2-carboxylates was synthesized and converted into 1,3-diketones *via* the Baker–Venkataraman rearrangement for constructing chromone and pyrazole frameworks.

## Graphical abstract

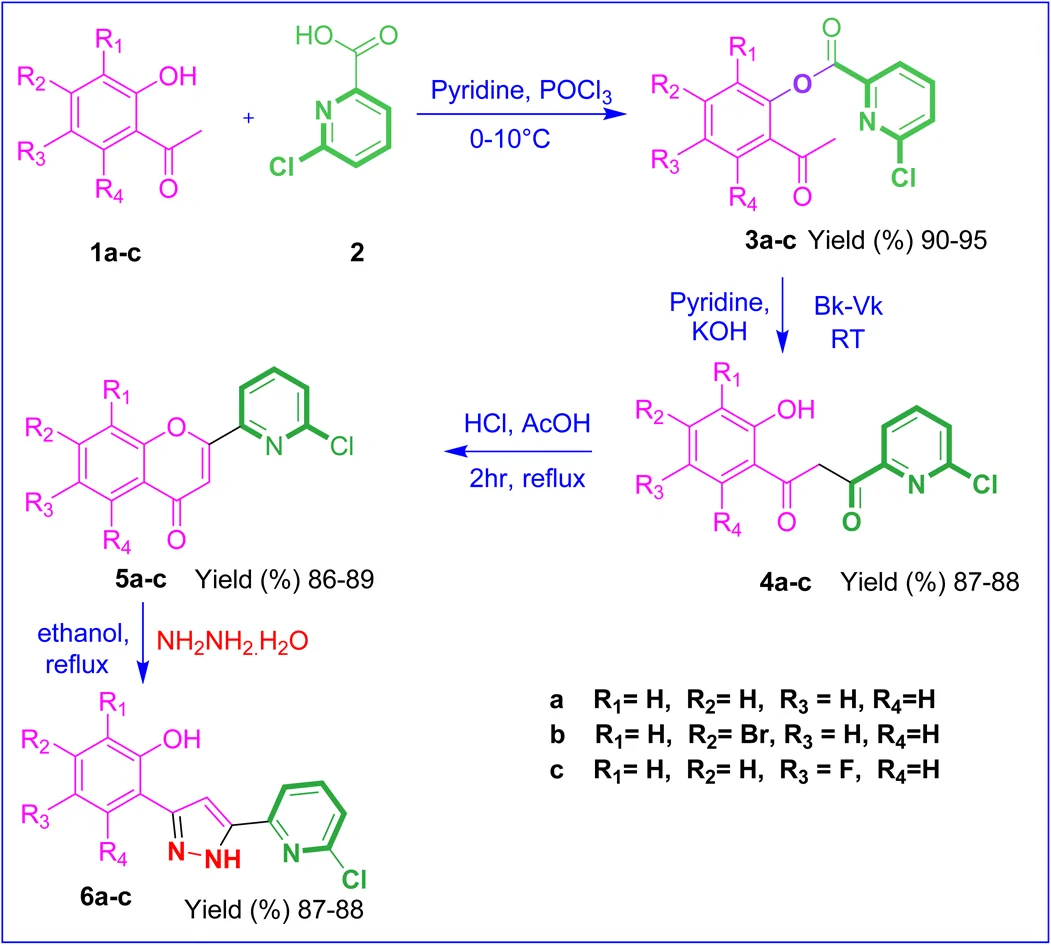

## 1. Introduction

Ramsay synthesized pyridine for the first time in 1877 by reacting hydrogen cyanide and acetylene in a hot tube furnace.^1,2^ Since then, pyridine has become one of the most significant nitrogen-containing heterocycles in chemistry and pharmacology. In addition to being a component of many natural compounds, such as vitamins, alkaloids, and coenzymes, the pyridine nucleus is found in a variety of medicinal substances.^3^ Pyridine improves the solubility and bioavailability of medicinal molecules because of its aromaticity, basicity, hydrogen-bonding capacity, and advantageous solubility properties. Because of these characteristics, it is an essential structural component in pharmaceutical chemistry.^4,5^

The rapid rise in microbial resistance has accelerated the hunt for new heterocyclic scaffolds with enhanced biological activity. Because of their varied pharmacophoric properties, carboxylic acids containing heteroatoms such as nitrogen are highly appealing. By conjugating the *o*-hydroxyacetophenone hydroxyl group with heterocyclic carboxylic acids, this work focuses on the design and synthesis of novel 2-acetylphenyl-6-chloropyridine carboxylate derivatives. This strategy emphasises the crucial role of heteroatoms and chloropyridine-based scaffolds in the development of novel antibacterial drugs.

Due to their major impact on metabolic stability, membrane permeability, and general pharmacokinetic behaviour, ester functionalities are also crucial in medicinal chemistry. Advances have further improved the efficiency and sustainability of esterification techniques, including the development of improved catalytic systems and environmentally friendly reaction media.^6^

Another well-known bioactive structural motif is the β-diketone framework. β-Diketone frameworks have a wide range of pharmacological effects, such as antibacterial,^7^ anti-inflammatory,^8^ anticancer,^9^ antiviral,^10^ antioxidant,^11^ and anticoagulant^12^ activities. Their anticancer properties have been thoroughly studied, highlighting their medicinal significance,^13^ and their powerful cytotoxic and enzyme-inhibitory potential makes them useful intermediates in drug development.^14,15^ β-Dicarbonyl complexes, which are closely related to β-diketones, are crucial building blocks for the synthesis of C–C bonds and heterocycles. Largely due to the antioxidant properties of their enol tautomer, compounds containing this moiety have shown therapeutic potential in the treatment of fibrosis,^16^ arthritis,^17^ obesity,^18^ hypertension,^19^ diabetes,^20^ neurological diseases,^21^ and inflammation.^22^ Research on molecules containing β-diketones is therefore still an active and promising field in chemical and medicinal chemistry. Another significant pharmacophore that is structurally related to β-diketone chemistry is the chromone scaffold. The most prevalent naturally occurring chromones, flavones, are found in a wide variety of plants and exhibit a range of biological activities.^23^ Antimicrobial,^24,25^ anti-inflammatory,^26^ antioxidant,^27^ antihistaminic,^28^ hepatoprotective,^29^ antitumor and anticancer activities,^30,31^ along with therapeutic effects in allergy disorders,^32^ are well-documented for these scaffolds. Styryl chromones have also demonstrated promising antiviral activity^33^ and protective effects against oxidative stress,^34–36^ underscoring the therapeutic flexibility of this scaffold.

The five-membered pyrazole ring is another important heterocyclic structure that has several uses in medicine. Because of their broad biological profile and structural versatility, pyrazole derivatives have been the subject of ongoing research for the past 40 years. They have antibacterial, anti-inflammatory,^37–41^ antiviral,^42^ anticancer,^43,44^ antiallergic,^45^ antihistaminic,^46^ anticonvulsant,^47,48^ antipyretic,^49^ anti-inflammatory,^50^ antidepressant,^51^ anti-arthritic,^52,53^ antihypercholesterolemic,^54^ and antifilarial properties.^55^

Crucially, the pyrazole nucleus is still useful in medicine because it is found in several therapeutically approved medications, such as lonazolac, celecoxib, ramifenazone, and phenylbutazone (Fig. 1).^56–60^ Given that pyridine, ester, β-diketone, chromone, and pyrazole frameworks have a wide range of pharmacological applications, combining these motifs into a single chemical structure represents a promising strategy for the development of new antimicrobial drugs. While β-diketone intermediates facilitate the synthesis of a variety of chromone and pyrazole derivatives, ester linkages offer structural flexibility for chemical modification.^61,62^

**Fig. 1:**
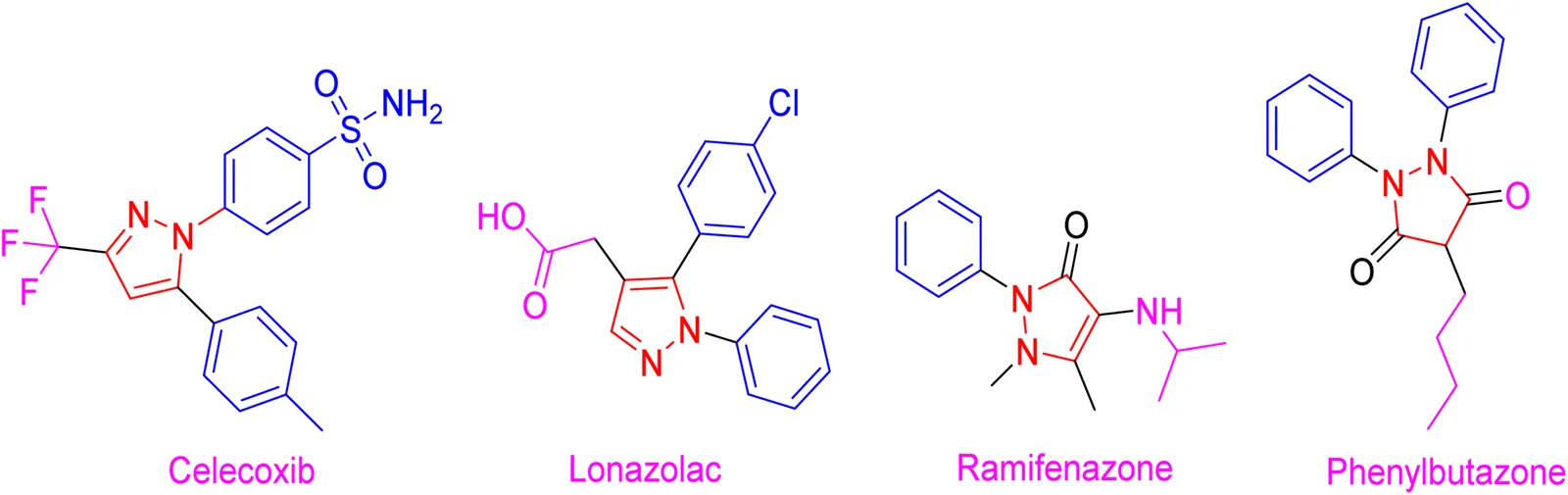
Some drugs bearing the pyrazole moiety.

Thus, the present study reports the synthesis and characterization of a novel series of ester, β-diketone, chromone and pyrazole derivatives, followed by a preliminary evaluation of their *in vitro* antimicrobial activity. The synthesized compounds were screened against selected Gram-positive and Gram-negative bacteria, yeast and filamentous fungi using the agar well diffusion method. The biological evaluation was intended to identify preliminary compound- and organism-dependent antimicrobial activity and to examine possible activity trends within the synthesized series.

## 2. Results and discussion

### 2.1. Chemistry

This study aimed to synthesise a series of ester, β-diketone, chromone, and pyrazole derivatives to generate structurally diverse molecules for the evaluation of their antimicrobial properties, given the increasing biological relevance of pyrazole-based scaffolds. The targeted esters (3a–c) were synthesised by coupling substituted *o*-hydroxy acetophenone derivatives (1a–c) with 6-chloropyridine-2-carboxylic acid (2) using pyridine and phosphorus oxychloride as activating agents. The positive test for ester (3a–c) compounds was confirmed by the hydroxamic acid test. Subsequent treatment of esters (3a–c) with potassium hydroxide in pyridine induced an intramolecular Claisen condensation reaction consistent with the Baker–Venkataraman (Bk–Vk) rearrangement, affording 1-(6-chloropyridin-2-yl)-3-(2-hydroxyphenyl)propane-1,3-diones (4a–c). Cyclodehydration of β-diketones (4a–c) in glacial acetic acid and concentrated hydrochloric acid under reflux yielded the corresponding 2-(6-chloropyridin-2-yl)-4*H*-chromen-4-ones (5a–c). As depicted in Scheme 1, these chromen-4-one derivatives (5a–c) underwent cyclisation upon reaction with hydrazine hydrate in glacial acetic acid and ethanol under reflux, producing the desired pyrazole derivatives, namely 2-(5-(6-chloropyridin-2-yl)-1*H*-pyrazol-3-yl)phenols (6a–c).

**Scheme 1:**
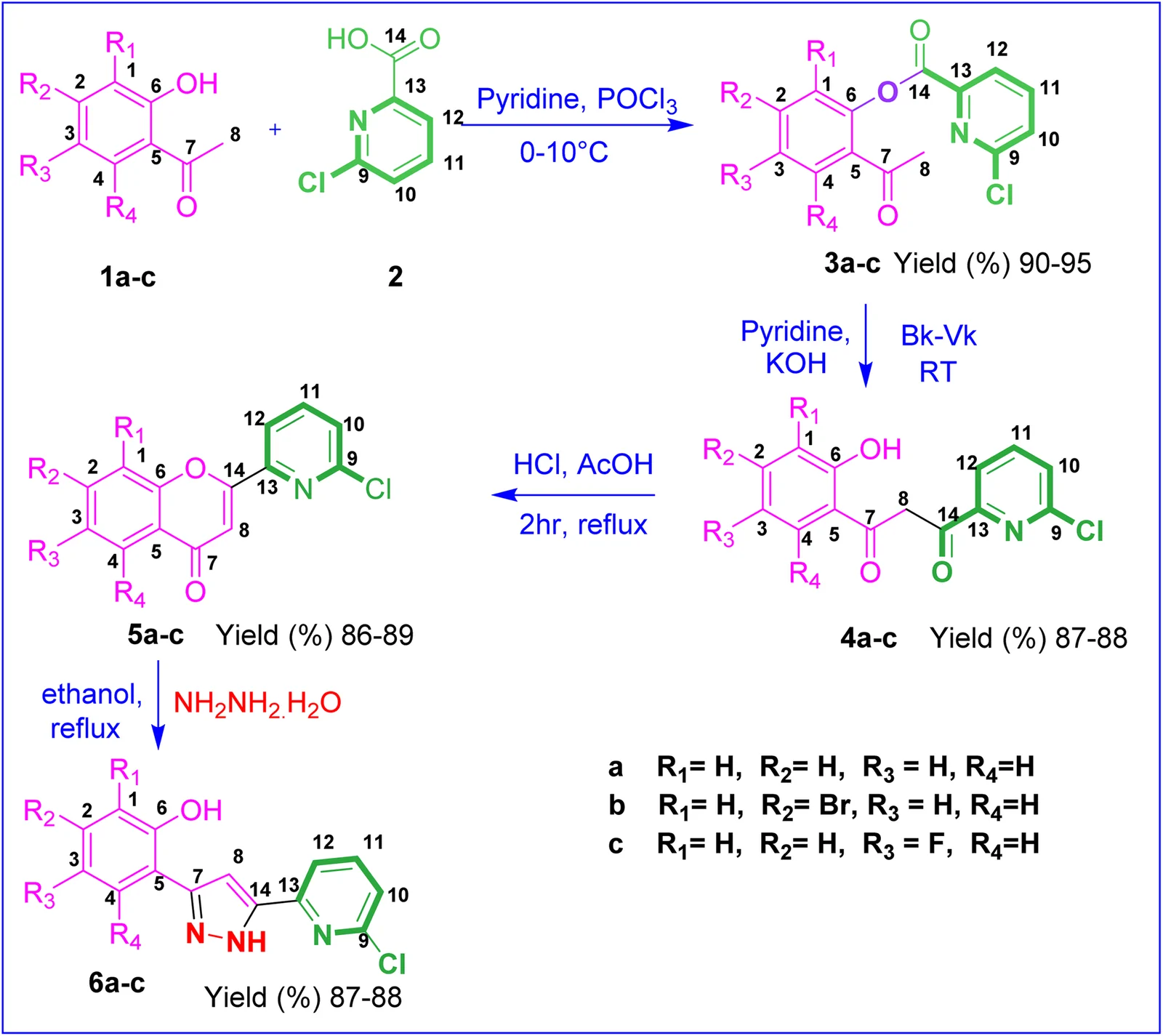
Synthesis of esters, β-diketones, chromones, and pyrazoles.

The structures of the synthesized compounds (3a–c), (4a–c), (5a–c), and (6a–c) were confirmed by FT-IR, ^1^H NMR, ^13^C NMR, and mass spectrometric analyses. For example, the infrared spectra of (3a–c) show an intense absorption band at around 1747 cm^−1^ for the ester –O–CO group, which occurs at higher frequencies than those of normal ketones because the force constant of the carbonyl bond is increased by the electron-attracting nature of the adjacent oxygen atom. The ^1^H NMR spectrum of 3a displayed a characteristic singlet at *δ* 2.58 ppm (s, 3H), which confirmed the esterification of 6-chloropyridine-2-carboxylic acid (2), and the aromatic protons resonated in the region of *δ* 8.24–7.26 ppm. The ^13^C NMR spectra of 3a showed aromatic carbon signals in the region of *δ* 151.8–123.6 ppm, a conjugated carbonyl ester peak appeared at *δ* 162.5 ppm, and a carbonyl carbon peak at *δ* 197.3 ppm. The mass spectra of 3a, 3b and 3c displayed molecular ion peaks [M + 1] at *m*/*z* 276.0406, 355.9466 and 294.0327, respectively, *via* HR-MS and at *m*/*z* 276.02, 355.95 and 294.04, respectively, *via* LC-MS, confirming the structures of the synthesised compounds. The structures of 3a, 3b and 3c were further confirmed by the observation of a fragment in their mass spectra that corresponded to protonated 6-chloropyridine-2-carboxylic acid.

The infrared spectra of 4a–c showed a strong and characteristic band for the 1,3-diketone linkage at 1712–1550 cm^−1^ and the enol –OH stretching at 3068 cm^−1^. The representative ^1^H NMR spectrum of 4a showed the disappearance of the singlet at around *δ* 2.58 ppm (corresponding to CO–CH_3_), but it displayed two sharp singlets corresponding to two protons at 15.03 ppm and 12.04 ppm, which confirm the presence of enolic proton (since the enol form in β-diketone is more stable) and phenolic –OH adjacent to the carbonyl group, respectively. The β-diketone compounds (4a–c) showed a negative hydroxamic acid test, indicating the absence of an ester functional group. The presence of characteristic ^1^H NMR signals is consistent with the structures of 4a–c, with aromatic protons observed in the region of 8.01–6.83 ppm. The mass spectrum of 4a displayed a molecular ion peak at *m*/*z* 276.04 [M + 1]. The infrared spectrum of 5a showed the appearance of absorption bands at 3062 cm^−1^ (–C–H stretching of –C=C–), 1641 cm^−1^ (C=C), 1602 cm^−1^ (Ar), 775 cm^−1^ (C–Cl). In addition, its ^1^H NMR spectrum supported its structure, as it revealed the chromone proton at *δ* 7.12 ppm (s, 1H, C-8). The mass spectrum of compound 5a showed a molecular ion peak at *m*/*z* 258.0342 [M + 1].

The infrared spectrum of 6a showed absorption bands at 3361 cm^−1^ (–OH), 3136 cm^−1^ (–NH), 1513 cm^−1^ (C=N), and 1173 cm^−1^ (C–Cl). In addition, its ^1^H NMR spectrum supported its structure, revealing the pyrazole ring proton at 7.76 (s, 1H). The mass spectrum of 6a displayed a molecular ion peak at *m*/*z* 272.95 [M + 1].

### 2.2. Key structure–activity relationship (SAR) insights derived from compounds 3a–3c, 4a–4c, 5a–5c, and 6a–6c

#### 2.2.1. Esters (3a–3c)

Activity trend: 3a > 3b ≫ 3c. According to this pattern, compound 3a exhibited the highest biological activity, followed by compound 3b with moderate activity, and compound 3c was found to be inactive. The maximum antibacterial and antifungal activities were demonstrated by 3a (unsubstituted phenyl), suggesting an ideal balance between steric and electronic factors. Despite improved lipophilicity, 3b (Br substitution) showed decreased activity due to steric hindrance. Furthermore, activity was entirely lost in 3c (F substitution), indicating that substantial electron-withdrawing effects at the *para* site are detrimental. Both steric variables and electronic distribution critically govern the antimicrobial activity, while the 6-chloropyridine-2-carboxylic acid core is essential for target contact.

#### 2.2.2. β-Diketones (4a–4c)

Trend against bacteria: 4b (Br) > 4a (H) > 4c (F). According to this pattern, compound 4b exhibited the highest biological activity, but it did not show any activity against *Pseudomonas* species. Compound 4a exhibited moderate activity but no activity against Gram-negative bacteria, and compound 4c showed relatively low activity and no activity against Gram-negative bacteria. Because of its enhanced polarisability and lipophilicity, 4b (*para*-Br) had the strongest antibacterial and antifungal activities. There was little improvement with fluorine substitution. The β-diketone pharmacophore probably increases activity by modulating the compound's lipophilicity, while the nucleus of chloropyridine plays a role in target interaction.

#### 2.2.3. Chromone derivatives (5a–5c)

Compound 5a established the baseline activity of the chromone-6-chloropyridine scaffold. Strong antifungal selectivity was demonstrated by 5b (7-Br). Broad-spectrum antibacterial and antifungal action was demonstrated by 5c (6-F). Through lipophilicity modification, electronic modulation, and halogen substitution, the potency was dramatically increased. Compound 5a lost its activity against *E. coli* and *Pseudomonas* species.

#### 2.2.4. Pyrazole-phenol derivatives (6a–6c)

Trend in antifungal activity: 6a > 6b > 6c. According to this pattern, compound 6a had the highest biological activity, but it did not show any activity against Gram-positive bacteria. Compound 6b exhibited moderate activity, while compound 6c displayed relatively low activity. Trend against bacteria: 6b ≥ 6a > 6c. Among the tested compounds, 6b showed the highest antibacterial activity, followed by 6a, while 6c was the least active. For hydrogen bonding to occur, the presence of free phenolic –OH group (6a) is essential. By increasing lipophilicity, Br substitution enhances antibacterial action. Activity is decreased by strong electron-withdrawing F substitution. It is necessary to strike an ideal balance between lipophilicity and hydrogen-bonding capabilities. Overall, our findings indicate that the 6-chloropyridine scaffold serves as an important pharmacophore, while moderate lipophilicity improves membrane penetration. The binding efficiency may be decreased by excessive steric bulk (Br). Strong electron-withdrawing effects (F) can reduce biological interactions. Compared to antibacterial activity, antifungal activity is more susceptible to structural changes.

### 2.3. Antibacterial and antifungal evaluation

The antimicrobial activity of the synthesized compounds was evaluated using the agar well diffusion method.^63,64^ The test microorganisms included three bacterial strains, *Escherichia coli*, *Pseudomonas* sp. and *Staphylococcus aureus*; one yeast, *Candida albicans*; and two filamentous fungi, *Aspergillus flavus* and *Aspergillus piperis*. The results are expressed as inhibition zone diameters (mm) and presented in Table 1.

**Table 1:** Inhibition zone diameters (mm) of the synthesized compounds against the tested microorganisms^a^

Bacteria		Gram −ve		Gram +ve	Yeast	Fungi	
Sr. no.	Sample	*Escherichia coli*	*Pseudomonas* species	*Staphylococcus aureus*	*Candida albicans*	*Aspergillus flavus*	*Aspergillus piperis*
1	3a	22 mm	—	15 mm	21 mm	21 mm	25 mm
2	3b	20 mm	—	15 mm	12 mm	12 mm	—
3	3c	—	—	—	—	—	—
4	4a	—	—	15 mm	17 mm	15 mm	19 mm
5	4b	22 mm	—	15 mm	16 mm	16 mm	19 mm
6	4c	—	—	14 mm	14 mm	12 mm	14 mm
7	5a	—	—	20 mm	13 mm	18 mm	—
8	5b	—	—	—	23 mm	33 mm	—
9	5c	18 mm	—	17 mm	18 mm	18 mm	23 mm
10	6a	18 mm	12 mm	—	15 mm	25 mm	28 mm
11	6b	19 mm	—	—	16 mm	15 mm	22 mm
12	6c	16 mm	16 mm	—	16 mm	—	—
Std.	**Gentamicin**	23 mm	24 mm	23 mm	—	—	—
Std.	**Amphotericin B (antifungal)**	—	—	—	24 mm	35 mm	30 mm

a —, no detectable inhibition under the experimental conditions.

The synthesized compounds exhibited variable and organism-dependent antimicrobial activities. Against *E. coli*, compounds 3a and 4b produced inhibition zones of 22 mm, compared with 23 mm for gentamicin. Activity against *Pseudomonas* sp. was comparatively limited, with compounds 6a and 6c producing inhibition zones of 12 and 16 mm, respectively, compared with 24 mm for gentamicin. Against *S. aureus*, compound 5a showed the largest inhibition zone (20 mm), followed by 5c (17 mm), compared with 23 mm for gentamicin.

Among the fungal test organisms, compound 5b showed appreciable activity against *C. albicans* and *A. flavus*, with inhibition zones of 23 and 33 mm, respectively. Compound 3a produced inhibition zones of 21 mm against *C. albicans* and 25 mm against *A. piperis*, while compound 6a showed inhibition zones of 25 and 28 mm against *A. flavus* and *A. piperis*, respectively. Amphotericin B produced inhibition zones of 24, 35 and 30 mm against *C. albicans*, *A. flavus* and *A. piperis*, respectively.

Overall, the results demonstrate compound- and microorganism-dependent differences in antimicrobial activity. However, inhibition zone measurements are influenced by factors such as compound concentration, solubility and diffusion through the agar medium and therefore should not be interpreted as direct quantitative measures of antimicrobial potency. The present results represent preliminary antimicrobial screening rather than evidence of superiority over standard antimicrobial agents.

Bacterial growth-curve and time-kill analyses were not performed in the present preliminary screening study; therefore, no conclusions regarding growth kinetics or time-dependent antimicrobial effects are drawn from the current data.

## 3. Experimental

### 3.1. Materials and methods

All solvents and reagents were utilised without further purification following procurement from commercial suppliers, such as Aldrich and Merck. The melting points are uncorrected and were determined in open capillary tubes utilising an Electrothermal 9100 apparatus. Thin-layer chromatography (TLC) was conducted on pre-coated silica gel aluminium plates to assess the purity of the chemicals. Electrospray ionisation (ESI) investigations were conducted utilising an ACQUITY ESI system with acetonitrile and 0.05% formic acid in water as the mobile phases. A Shimadzu FT-IR 8400 spectrometer was employed to acquire the FT-IR (ATR) spectra within the 400–4000 cm^−1^ range. A Bruker Avance 400 MHz spectrometer was employed to acquire ^1^H NMR and ^13^C NMR spectra in CDCl_3_ and DMSO-d_6_.

### 3.2. General procedure for the synthesis of ester compounds 3a–c

Anhydrous pyridine (10 mL) was used to dissolve a mixture of the substituted o-hydroxy acetophenone (1a: 1.36 g, 1b: 2.13 g, or 1c: 1.54 g; 10 mmol) and 6-chloropyridine-2-carboxylic acid (1.56 g, 10 mmol). Phosphorus oxychloride (10 mmol) was added dropwise to the reaction flask while the mixture was maintained in an ice bath, keeping the temperature between 0 and 10 °C. Upon complete addition, the reaction mixture was stirred for 2 h and allowed to stand at ambient temperature overnight. The reaction was then quenched by carefully pouring the mixture onto crushed ice, followed by acidification with cold, dilute hydrochloric acid. The resulting off-white solid was collected, dried, and subsequently recrystallised from ethanol to afford the pure products 3a–c (Scheme 1).

### 3.3. Spectral data of compounds 3a–c

#### 3.3.1. 2-Acetylphenyl 6-chloropyridine-2-carboxylate (3a)

Yield: 2 g (95%), colour: off-white solid, M.P.: 111–112 °C; FTIR (cm^−1^): 1747 (ester C=O), 1614 (C=O), 1186 (C–O), 1116 (C–Cl); ^1^H NMR (400 MHz, CDCl_3_) *δ* (ppm): 8.24–8.22 (dd, *J* = 8.2 Hz 1H, H Ar) (C-11), 7.91–7.87 (m, 2H) (C-10 and C-12), 7.62–7.58 (m, 2H, H Ar) (C-1 and C-3), 7.41–7.38 (m, 1H, H Ar) (C-4), 7.39–7.26 (m, 1H, H Ar) (C-2), 2.58 (s, 3H, CH_3_) (C-8); mass spectrum, *m*/*z* (*I*_rel_, %): 278 (55) [M(^37^Cl) + H]^+^, 276 (95) [M(^35^Cl) + H]^+^, 160 (60), 158 (100). Calculated mass: 276.69 [M + 1], HR-MS: 276.0406 [M + 1], LC-MS: 276.02 [M + 1] C_14_H_10_ClNO_3_. ^13^C NMR *δ* (ppm): 197.3 (C-7), 162.5 (C-14), 151.8 (C-6), 149.1 (C-13), 147.6 (C-9), 139.8 (C-11), 133.6 (C-2), 130.5 (C-4), 130.4 (C-10), 128.6 (C-5), 126.5 (C-3), 126.5 (C-12), 123.6 (C-1), 29.4 (C-8).

#### 3.3.2. 2-Acetyl-5-bromophenyl 6-chloropyridine-2-carboxylate (3b)

Yield: 2.3 g (90%), colour: off white, mp: 154–155 °C; FT-IR (cm^−1^): 1739 (ester C=O), 1676 (C=O), 1199 (C–Cl), 1141 (C–Cl); ^1^H NMR (400 MHz, CDCl_3_), *δ* (ppm) 8.22–820 (dd, *J* = 8.2 Hz, 1H, H Ar) (C-11), 7.98–7.97 (d, *J* = 2.4 Hz, 1H) (C-12), 7.91–7.87 (t, *J* = 8 Hz, 1H) (C-10), 7.72–7.69 (dd, *J* = 7.7 Hz, 1H) (C-1), 7.62–7.60 (dd, *J* = 7.6 Hz, 1H) (C-3), 7.26–7.18 (m, 1H) (C-4), 2.57 (s, 3H) (C-8); mass spectrum, *m*/*z* (*I*_rel_, %): 358 (15) [M + H]^+^, 356 (55) [M + H]^+^, 354 (40) [M + H]^+^, 160 (33), 158 (100), calculated mass: 355.58 [M + 1], HR-MS: 355.9466 [M + 1], LC-MS: 355.95 [M + 1] C_14_H_9_BrClNO_3_. ^13^C NMR *δ* (ppm): 195.9 (C-7), 162.3 (C-14), 152.0 (C-6), 148.1 (C-13), 147.2 (C-9), 139.1 (C-11), 136.3 (C-4), 130.3 (C-3), 132.1 (C-2), 128.5 (C-10), 125.5 (C-5), 126.4 (C-1), 129.6 (C-12), 29.5 (C-8).

#### 3.3.3. 2-Acetyl-4-fluorophenyl 6-chloropyridine-2-carboxylate (3c)

Yield: 2.1 g (90%), colour: off white, mp: 145–146 °C; FT-IR (cm^−1^): 1751 (ester C=O), 1676 (C=O) 1174 (C–O), 1099 (C–Cl); ^1^H NMR (400 MHz, CDCl_3_), *δ* (ppm): 8.22–8.20 (dd, *J* = 8.2 Hz, 1H, H Ar) (C-11), 7.91–7.87 (t, *J* = 7.9 Hz, 1H) (C-12), 7.62–7.60 (t, *J* = 7.6 Hz, 1H) (C-10), 7.57–7.54 (dd, *J* = 2.4 Hz, *J* = 7.6 Hz, 1H) (C-4), 7.32–7.26 (m, 2H) (C-1 and C-2), 2.57 (s, 3H) (C-8); mass spectrum, *m*/*z* (*I*_rel_, %): 296 (32) [M(^37^Cl) + H]^+^, 294 (80) [M(^35^Cl) + H]^+^, 160 (60), 158 (100), calculated mass: 294.68 [M + 1], HR-MS: 294.0327 [M + 1], LC-MS: 294.04 [M + 1] C_14_H_9_FClNO_3_, ^13^C NMR *δ* (ppm): 196.00 (C-7), 162.6 (C-3), 151.9 (C-14), 147.3 (C-6), 145.0 (C-13), 139.8 (C-9), 131.7 (C-11), 128.7 (C-5), 125.5 (C-10), 124.4 (C-12), 120.4 (C-1), 117.2 (C-2), 116.6 (C-4), 29.5 (C-8).

### 3.4. General procedure for the synthesis of β-diketone compounds 4a–c

Dry pyridine (10 mL) was used to dissolve the ester 2-acetylphenyl 6-chloropyridine-2-carboxylate (3a: 0.82 g, 3b: 1.059 g, or 3c: 0.88 g; 3 mmol). The reaction solution was then treated with powdered potassium hydroxide (0.17 g, 3 mmol) under continuous stirring. The mixture was stirred at room temperature for 3 h, and TLC was used to monitor the reaction progress. Upon completion, the reaction mass was poured onto crushed ice and acidified with concentrated hydrochloric acid. The resulting pale-yellow solid was collected by filtration and recrystallised from ethanol to afford the pure compounds 4a–c (Scheme 1).

### 3.5. Spectral data of compounds 4a–c

#### 3.5.1. 1-(6-Chloropyridin-2-yl)-3-(2-hydroxyphenyl)propane-1,3-dione (4a)

Yield: 1 g (87%), colour: yellow, mp: 119–120 °C; FT-IR (cm^−1^): 3123 (–OH), 3068 (enol –OH), 1712 (C=O), 739 (C–Cl); ^1^H NMR (400 MHz, CDCl_3_), *δ* (ppm): 15.03 (s, 1H, enolic H), 12.04 (s, 1H, OH), 8.01–7.82 (m, 2H) (C-11 and C-12), 7.80 (s, 1H, =CH) (C-8), 7.53–7.26 (m, 3H) (C-2, C-4 and C-10), 7.02–6.83 (d, 2H) (C-1 and C-3), calculated mass: 276.69 [M + 1], HR-MS: 276.0425 [M + 1], C_14_H_11_ClNO_3_.

#### 3.5.2. 1-(4-Bromo-2-hydroxyphenyl)-3-(6-chloropyridin-2-yl)propane-1,3-dione (4b)

Yield: 0.9 g (88%), colour: yellow, mp: 162–163 °C; FT-IR (cm^−1^): 3073 (OH), 1550 (C=O), 1137 (C–O); ^1^H NMR (400 MHz, DMSO), *δ* (ppm): 11.31 and 11.17 (s, 1H, –OH), 8.35–8.12 (m, 2H) (C-11 and C-12), 8.11–8.07 (m, 2H) (C-8), 8.06–8.00 (m, 1H) (C-10),7.88–7.85 (m, 1H) (C-4), 7.83–7.71 (m, 1H) (C-2), 7.70–7.48 (m, 1H) (C-1), calculated mass: 355.58 [M + 1], HR-MS: 355.6523 [M + 1], 353.9536 [M − 1] C_14_H_10_ClNO_3_Br.

#### 3.5.3. 1-(6-Chloropyridin-2-yl)-3-(5-fluoro-2-hydroxyphenyl)propane-1,3-dione (4c)

Yield: 1.1 g (87%), colour: yellow, mp: 160–161 °C; FT-IR (cm^−1^): 3077 (OH), 1549 (C=O), 1173 (C–Cl); ^1^H NMR (400 MHz, DMSO), *δ* (ppm): 11.32 (s, 1H, –OH), 8.34–8.06 (m, 1H) (C-12), 8.00 (m, 1H) (C-11), 7.92 (m, 2H) (C-8), 7.83 (m, 1H) (C-10), 7.71–7.69 (m, 2H) (C-1 and C-2), 7.57–7.56 (m, 1H) (C-4), calculated mass: 294.68 [M + 1], HR-MS: 294.0342 [M + 1], C_14_H_9_ClFNO_3_.

### 3.6. General procedure for the synthesis of chromone compounds 5a–c

To a solution of 1-(6-chloropyridin-2-yl)-3-(2-hydroxyphenyl)propane-1,3-dione (4a: 0.275 g, 4b: 0.354 g, or 4c: 0.293 g; 1 mmol) in ethanol (10 mL), concentrated hydrochloric acid (1 mL, 0.01 mol) was added. The reaction was refluxed for 2 h, after which it was cooled to room temperature and then poured onto crushed ice. The resulting solid was collected, filtered, and recrystallised from ethanol to afford the products 5a–c (Scheme 1).

### 3.7. Spectral data of compounds 5a–c

#### 3.7.1. 2-(6-Chloropyridin-2-yl)-4*H*-chromen-4-one (5a)

Yield: 0.9 g (86%), colour: off white, mp: 166–168 °C; FT-IR (cm^−1^): 3062 (–C–H stretching of –C=C–), 1641 (C=C), 1602 (Ar), 775 (C–Cl); ^1^H NMR (400 MHz, DMSO), *δ* (ppm): 8.27–8.26 (d, 1H, *J* = 7.6 Hz) (C-12), 8.17–8.14 (t, 1H, *J* = 7.6 Hz) (C-11), 8.09–8.06 (d, 1H, *J* = 1.6 Hz) (C-10), 7.89–7.77 (m, 2H) (C-2 and C-4), 7.75 (m, 1H) (C-1), 7.54 (m, 1H) (C-3), 7.12 (s, 1H) (C-8), calculated mass: 258.67 [M + 1], HR-MS: 258.0342 [M + 1], C_14_H_8_ClNO_2_, ^13^C NMR *δ* (ppm): 177.5 (C-7), 160.2 (C-14), 156.0 (C-6), 151.0 (C-13), 149.5 (C-9), 142.0 (C-11), 135.2 (C-2), 127.5 (C-4), 126.3 (C-5), 125.4 (C-3), 124.2 (C-10), 121.0 (C-12), 119.2 (C-1), 109.6 (C-8).

#### 3.7.2. 7-Bromo-2-(6-chloropyridin-2-yl)-4*H*-chromen-4-one (5b)

Yield: 0.8 g (88%), colour: off white, mp: 138–139 °C; FT-IR (cm^−1^): 3059 (–C–H stretching of –C=C–), 1699 (C=C), 1634 (Ar), 795 (C–Cl); ^1^H NMR (400 MHz, DMSO), *δ* (ppm): 8.34 (d, *J* = 7.6 Hz, 1H) (C-11), 8.22–8.12 (m, 2H) (C-10 and C-12), 7.94–7.92 (d, *J* = 8.4 Hz, 1H) (C-4), 7.78–7.72 (m, 1H) (C-3), 7.62–7.60 (d, *J* = 8 Hz, 1H) (C-1),7.15 (s, 1H) (C-8), calculated mass: 337.57 [M + 1], HR-MS: 337.9397 [M + 1], 335.9429 [M − 1], C_14_H_7_BrClNO_2_, ^13^C NMR *δ* (ppm): 156.3 (C-7), 154.1 (C-14), 154.0 (C-6), 150.0 (C-13), 149.4 (C-9), 148.3 (C-11), 142.2 (C-4), 141.5 (C-2), 135.8 (C-3), 131.3 (C-10), 128.7 (C-5), 127.7 (C-1), 124.2 (C-12), 122.1 (C-8).

#### 3.7.3. 2-(6-Chloropyridin-2-yl)-6-fluoro-4*H*-chromen-4-one (5c)

Yield: 1 g (89%), colour: off white, mp: 134–135 °C; FT-IR (cm^−1^): 3053 (–C–H stretching of –C=C–), 1690 (C=C), 1560 (Ar), 792 (C–Cl); ^1^H NMR (400 MHz, DMSO), *δ* (ppm): 8.29–8.18 (t, 1H) (C-11), 8.16–8.15 (d, *J* = 7.6 Hz, 1H) (C-10), 8.10–8.08 (d, *J* = 8 Hz, 1H) (C-12), 7.89–7.88 (d, 1H) (C-2), 7.85–7.76 (t, 1H) (C-1), 7.75 (s, 1H) (C-4), 7.14 (s, 1H) (C-8), calculated mass: 276.66 [M + 1], HR-MS: 276.0217 [M + 1], C_14_H_7_ClFNO_2_, ^13^C NMR *δ* (ppm): 156.3 (C-7), 154.5 (C-14), 154.0 (C-3), 150.0 (C-13), 149.4 (C-6), 148.2 (C-9), 142.2 (C-11), 141.9 (C-5), 141.5 (C-10), 138.5 (C-2), 137.7 (C-12), 128.7 (C-1), 127.5 (C-4), 124.6 (C-8).

### 3.8. General procedure for the synthesis of pyrazole compounds 6a–c

A 1 mmol solution of 2-(6-chloropyridin-2-yl)-4*H*-chromen-4-one (5a: 0.257 g, 5b: 0.334 g, or 5c: 0.275 g) in ethanol (15 mL) was placed in a 100 mL round-bottom flask. Hydrazine hydrate (0.150 g, 3 mmol) was added, and the reaction mixture was heated under reflux for 2 h. A catalytic amount of glacial acetic acid was then introduced, and reflux was continued for an additional 2 h. After completion, the mixture was allowed to cool to room temperature and subsequently poured onto crushed ice. The resulting solid was collected by filtration and recrystallised from acetic acid to afford the desired compounds 6a–c (Scheme 1).

### 3.9. Spectral data of compounds 6a–c

#### 3.9.1. 2-(5-(6-Chloropyridin-2-yl)-1*H*-pyrazol-3-yl)phenol (6a)

Yield: 0.5 g (88%), colour: brown, mp: 144–145 °C; FT-IR (cm^−1^): 3361 (–OH), 3136 (–NH), 1513 (C=N), 1173 (C–Cl); ^1^H NMR (400 MHz, DMSO), *δ* (ppm) 13.30 (s, 1H, NH), 10.56 (b, s, 1H, OH), 7.98–7.78 (m, 2H) (C-11 and C-12),7.76 (s, 1H) (C-8), 7.47–7.45 (d, *J* = 8 Hz, 1H) (C-10),7.37 (s, 1H) (C-4), 7.23–7.00 (d, *J* = 4 Hz 1H) (C-2), 6.98–6.89 (m, 2H) (C-1 and C-3), calculated mass: 272.70 [M + 1], HR-MS: 272.9595 [M + 1], C_14_H_10_ClN_3_O, ^13^C NMR *δ* (ppm): 157.0 (C-13), 153.6 (C-6), 152.2 (C-9), 150.4 (C-7), 142.2 (C-14), 122.5 (C-11), 118.1 (C-2), 117.0 (C-4), 116.0 (C-12), 115.6 (C-3), 114.8 (C-10), 113.5 (C-5),112.2 (C-1), 102.9 (C-8).

#### 3.9.2. 5-Bromo-2-(5-(6-chloropyridin-2-yl)-1*H*-pyrazol-3-yl)phenol (6b)

Yield: 0.6 g (87%), colour: brown, mp: 124–125 °C; FT-IR (cm^−1^): 3362 (OH), 3133 (NH), 1512 (C=N), 1178 (C–Cl); ^1^H NMR (400 MHz, DMSO), *δ* (ppm) 13.65 (s, 1H, NH), 10.42–10.39 (s, 1H, –OH), 7.96 (s, 2H) (C-10 and C-12), 7.47–7.05 (m, 3H) (C-1, C-3 and C-4), 7.03–6.99 (m, 2H) (C-11 and C-8), calculated mass: 351.60 [M + 1], HR-MS: 351.9671 [M + 1], 349.9695 [M − 1], C_14_H_9_ClBrN_3_O, ^13^C NMR *δ* (ppm): 157.1 (C-6), 154.8 (C-13), 151.3 (C-9), 150.5 (C-7), 141.1 (C-14), 123.5 (C-11), 119.0 (C-4), 117.7 (C-3), 117.8 (C-2), 116.0 (C-12), 115.8 (C-10), 113.4 (C-1),113.2 (C-5), 103.9 (C-8).

#### 3.9.3. 2-(5-(6-Chloropyridin-2-yl)-1*H*-pyrazol-3-yl)-4-fluorophenol (6c)

Yield: 0.7 g (87%), colour: brown, mp: 153–154 °C; FT-IR (cm^−1^): 3348 (OH), 3125 (NH), 1620 (C=N), 1173 (C–Cl); ^1^H NMR (400 MHz, DMSO), *δ* (ppm) 13.65 (s, 1H, NH), 10.70 (s, 1H, –OH), 7.96 (s, 3H) (C-10, C-11 and C-12) 7.47–7.34 (m, 3H) (C-1, C-2 and C-4) 6.96–6.94 (m, 1H) (C-8), calculated mass: 290.69 [M + 1], HR-MS: 290.9917 [M + 1], C_14_H_9_ClFN_3_O, ^13^C NMR *δ* (ppm): 172.5 (C-3), 154.3 (C-13), 150.5 (C-6), 141.1 (C-9), 131.9 (C-7), 129.6 (C-14), 123.6 (C-11), 119.9 (C-12), 119.0 (C-5), 116.0 (C-10), 114.0 (C-1), 111.0 (C-4), 104.0 (C-2), 21.51 (C-8).

### 3.10. Antimicrobial activity

#### 3.10.1. Microbial strains and culture preparation

The antimicrobial activity of the synthesized compounds was evaluated against *Escherichia coli*, *Pseudomonas* sp., *Staphylococcus aureus*, *Candida albicans*, *Aspergillus flavus*, and *Aspergillus piperis*. Fresh 18–24 h cultures of *E. coli*, *Pseudomonas* sp., and *S. aureus* were used for the bacterial inoculum preparation. For *C. albicans*, a fresh 24–48 h culture was used, whereas 5–7-day-old cultures of *A. flavus* and *A. piperis* were used for the preparation of fungal spore suspensions. For bacterial inoculum preparation, 3–5 well-isolated colonies were suspended in sterile 0.85% saline. Fresh cultures were used to ensure microbial viability and reproducibility of the antimicrobial assay, while 5–7-day-old *Aspergillus* cultures were used to obtain adequate sporulation for the preparation of fungal inocula.

#### 3.10.2. Preparation of test compounds and controls

The synthesized compounds (3a–6c) were dissolved in dimethyl sulfoxide (DMSO), and the solutions were filter-sterilized using a 0.22-µm membrane filter. The final concentration of DMSO in the assay was maintained at 1–2% (v/v). Gentamicin (10 µg/50 µL) was used as the positive control for the bacterial strains, whereas amphotericin B (10 µg/50 µL) was used as the positive control for *C. albicans* and the filamentous fungi. DMSO (≤1–2%, v/v) was used as the negative/vehicle control.

#### 3.10.3. Agar well diffusion assay

The antimicrobial activity of the synthesized compounds was evaluated using the agar well diffusion method. Mueller–Hinton agar (MHA) was used for the bacterial cultures, whereas potato dextrose agar (PDA) was used for *C. albicans* and the filamentous fungi. The prepared microbial inocula were uniformly spread over the surface of the respective agar plates. The inoculated plates were allowed to dry for 3–5 min, after which 6 mm diameter wells were aseptically prepared using a sterile cork borer. Subsequently, 50 µL of each test compound solution was introduced into the respective wells. The loaded plates were kept at room temperature for 30–45 min to allow diffusion of the compounds into the agar before incubation.

#### 3.10.4. Incubation and measurement of inhibition zones

The bacterial assay plates were incubated at 35 ± 2 °C for 16–20 h. Plates inoculated with *C. albicans* were incubated at 30–35 °C for 24–48 h, whereas plates containing *Aspergillus* spp. were incubated at 25–28 °C for 48–72 h. After incubation, antimicrobial activity was determined by measuring the diameter of the clear inhibition zone around each well in millimetres, including the 6 mm well diameter. Measurements were taken along different orientations of each inhibition zone, and the mean zone diameter was recorded. The results of the agar well diffusion assay were expressed as zones of inhibition (mm) and not as minimum inhibitory concentrations (MICs).

## 4. Conclusion

In this study, a series of ester, β-diketone, chromone and pyrazole derivatives containing the 6-chloropyridine scaffold were synthesized, characterized and evaluated for *in vitro* antimicrobial activity. The synthesized derivatives exhibited variable and organism-dependent activity, with selected compounds showing appreciable inhibition against the tested bacteria, yeast and filamentous fungi. A significant outcome of the present synthesis is the development of heterocyclic derivatives exhibiting both antibacterial and antifungal activities. Several of the synthesized compounds demonstrated promising antimicrobial potential against the tested bacterial and fungal strains, indicating that these derivatives possess a broad-spectrum antimicrobial profile. The simultaneous antibacterial and antifungal activities observed for selected derivatives highlight their biological significance.

## Conflicts of interest

The authors declare that there are no conflicts of interest regarding the publication of this paper.

## Supplementary Material

RA-OLF-D6RA07223B-s001

## Data Availability

The data supporting the findings of this study are available from the corresponding author upon reasonable request. All experimental procedures, spectral characterization data (including FT-IR spectroscopy, NMR, and mass spectrometry), antimicrobial activity results, and other relevant data generated and analyzed during this study are provided within the article and its supplementary information (SI). No additional datasets were generated beyond those presented in this manuscript. Supplementary information is available. See DOI: https://doi.org/10.1039/d6ra07223b.
