# Supplementary material for: Synthesis and evaluation of the antimicrobial activity of novel esters, β-diketones, chromones and pyrazoles
Source: RSC Adv. 2026 Oct 7;16(46):60671–80. doi: 10.1039/d6ra07223b (PMC13643702; doi:10.1039/d6ra07223b)
Supplement: Supplementary file 1 [file RA-016-D6RA07223B-s001.pdf]

Supplementary Information (SI) for RSC Advances.
This journal is © The Royal Society of Chemistry 2026

Synthesis and evaluation of antimicrobial activity of novel Esters, β-diketones, Chromones and Pyrazoles

A. S. Shinde[1], S. P. Badadhe[2], S. P. Kunde[1], D. N. Gaikwad[1], B. S. Pawar[3], P. V. Badadhe*[1]

1. P. G. Department of Chemistry and Research Centre, R. B. Narayanrao Borawake College, Shrirampur, M.S., India 413709
2. P. G. Department of Chemistry and Research Centre, S. M. Joshi College, Hadapsar, M.S., India. 411028
3. Department of Microbiology, Yashavantrao Chavan Institute of Science, A Lead College of Karmveer Bhaurao Patil University, Satara, M.S., India 415001

[Affiliated to Savitribai Phule Pune University, Maharashtra] India. (413709).

Corresponding Author Email: pvbadadhe@gmail.com

Spectra IR, ^{1}H NMR, ^{13} C NMR, MS.

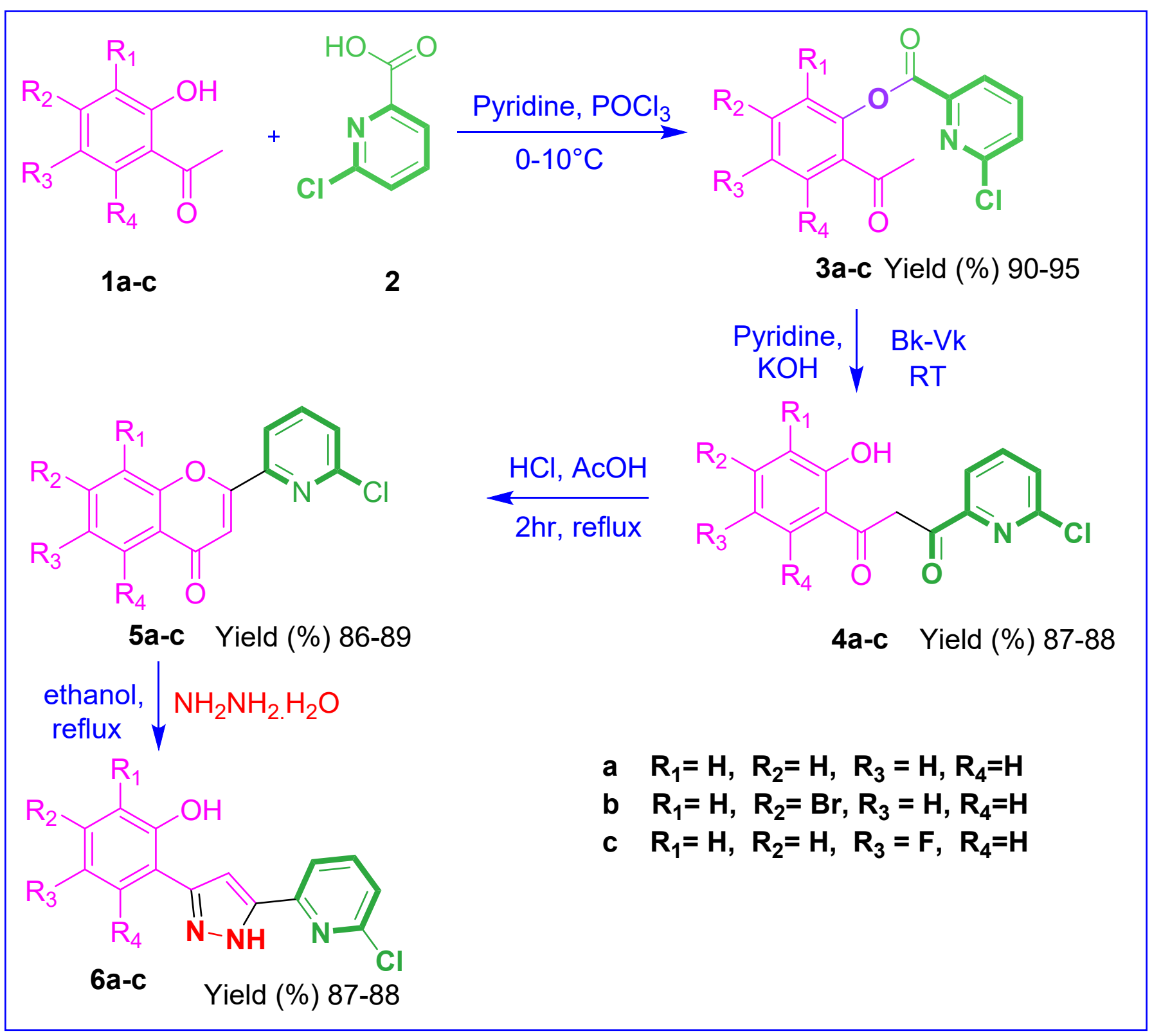

3a-c, 4a-c, 5a-c, 6a-c.

S1-3a IR

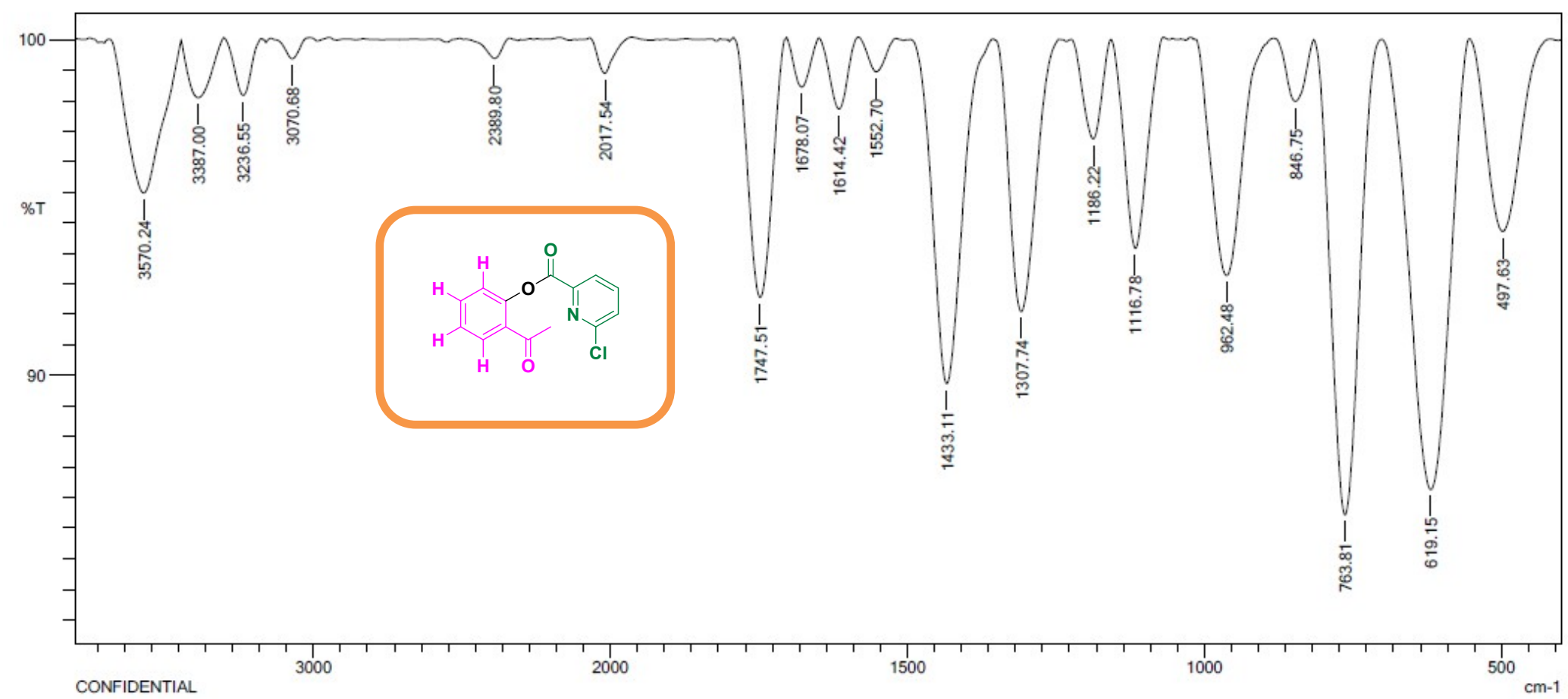

S2- 3a ^{1}H NMR

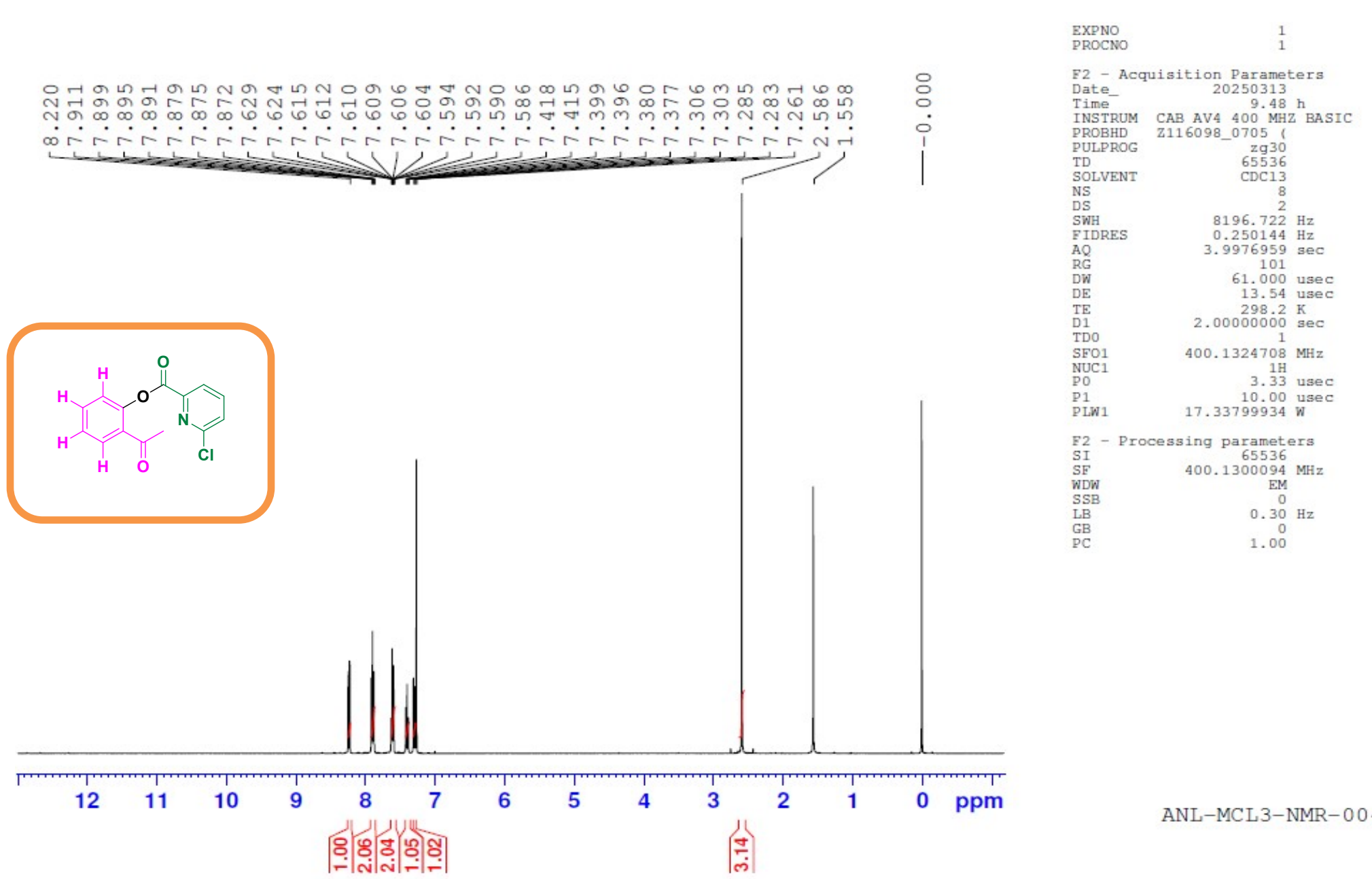

S3-3a ^{13} C NMR

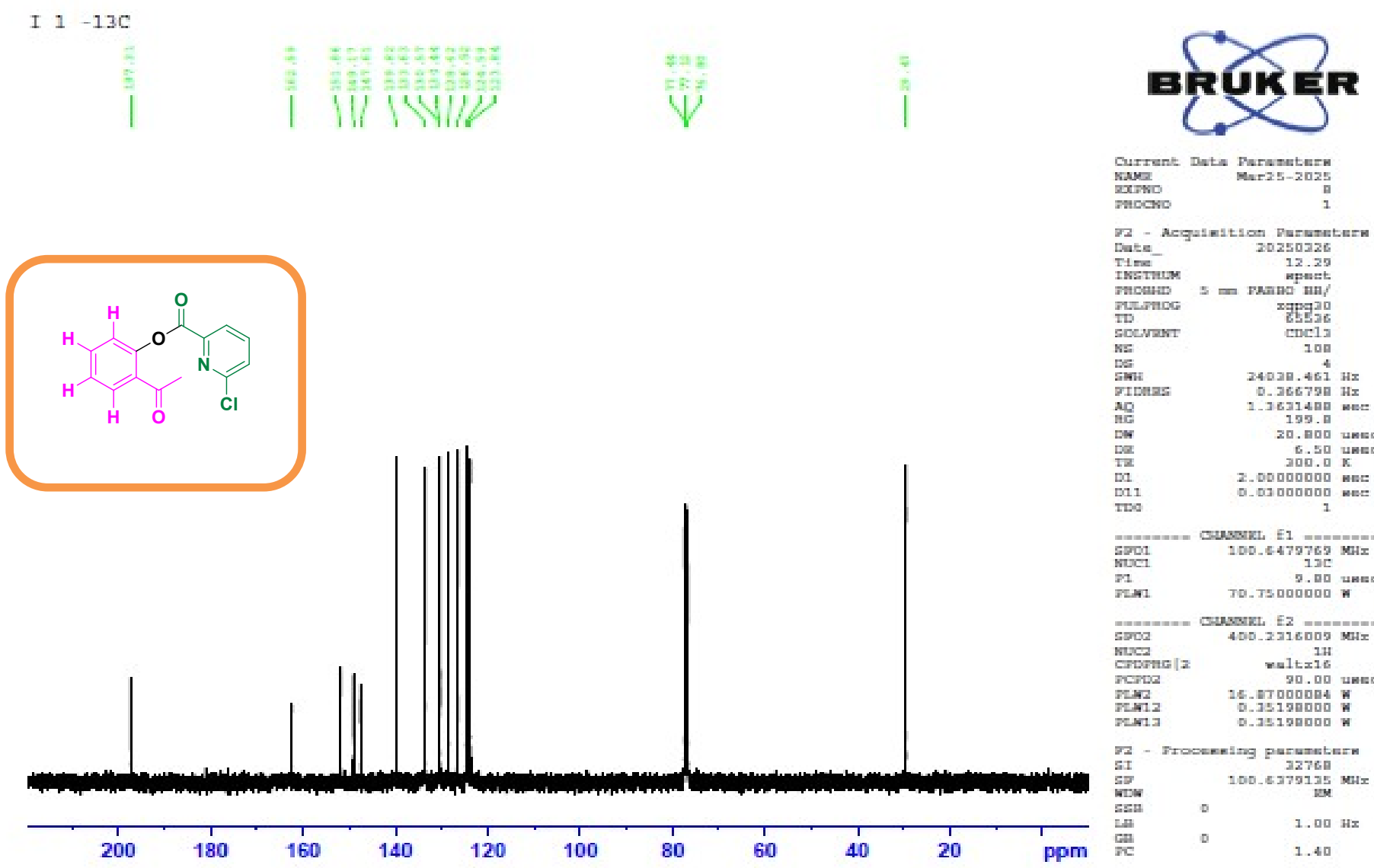

LC-MS -3a

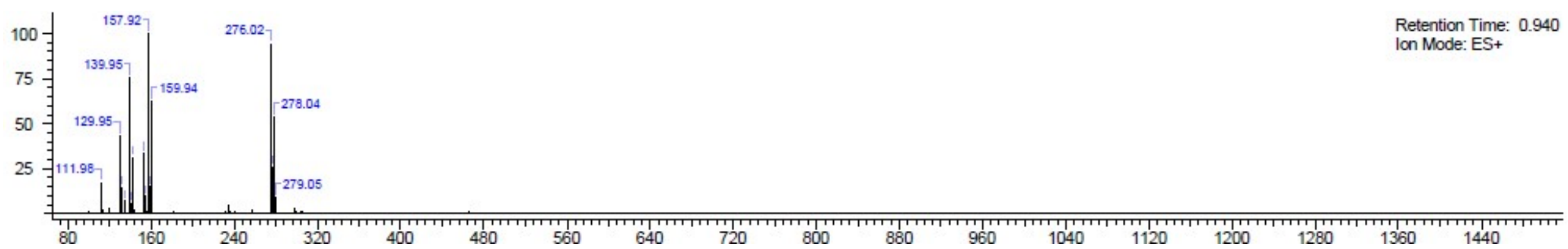

S4-3a HR-MS

Savitribai Phule Pune University - Central Instrumentation Facility

Analysis Info

Analysis Name D:\Data\2026\APR\SPPU COLLEGE\RBNB COLLEGE SHRIRAMPUR\ARUNA SHINDE\3a_RD4_01_12380.d

Method dlc_50-1200mz_pos_mode_3min_0.2ml_apr-2026.m

Sample Name 3a

Acquisition Date 4/9/2026 1:50:44 PM

Analyst : Nitin S. Kadam CIF

Instrument impact HD 1819696.00184

Acquisition Parameter

Source Type	ESI	Ion Polarity	Positive	Set Nebulizer	0.7 Bar
Focus	Active	Set Capillary	4000 V	Set Dry Heater	200 °C
Scan Begin	50 m/z	Set End Plate Offset	-500 V	Set Dry Gas	4.0 l/min
Scan End	1200 m/z	Set Charging Voltage	2000 V	Set Divert Valve	Source
		Set Corona	0 nA	Set APCI Heater	0 °C

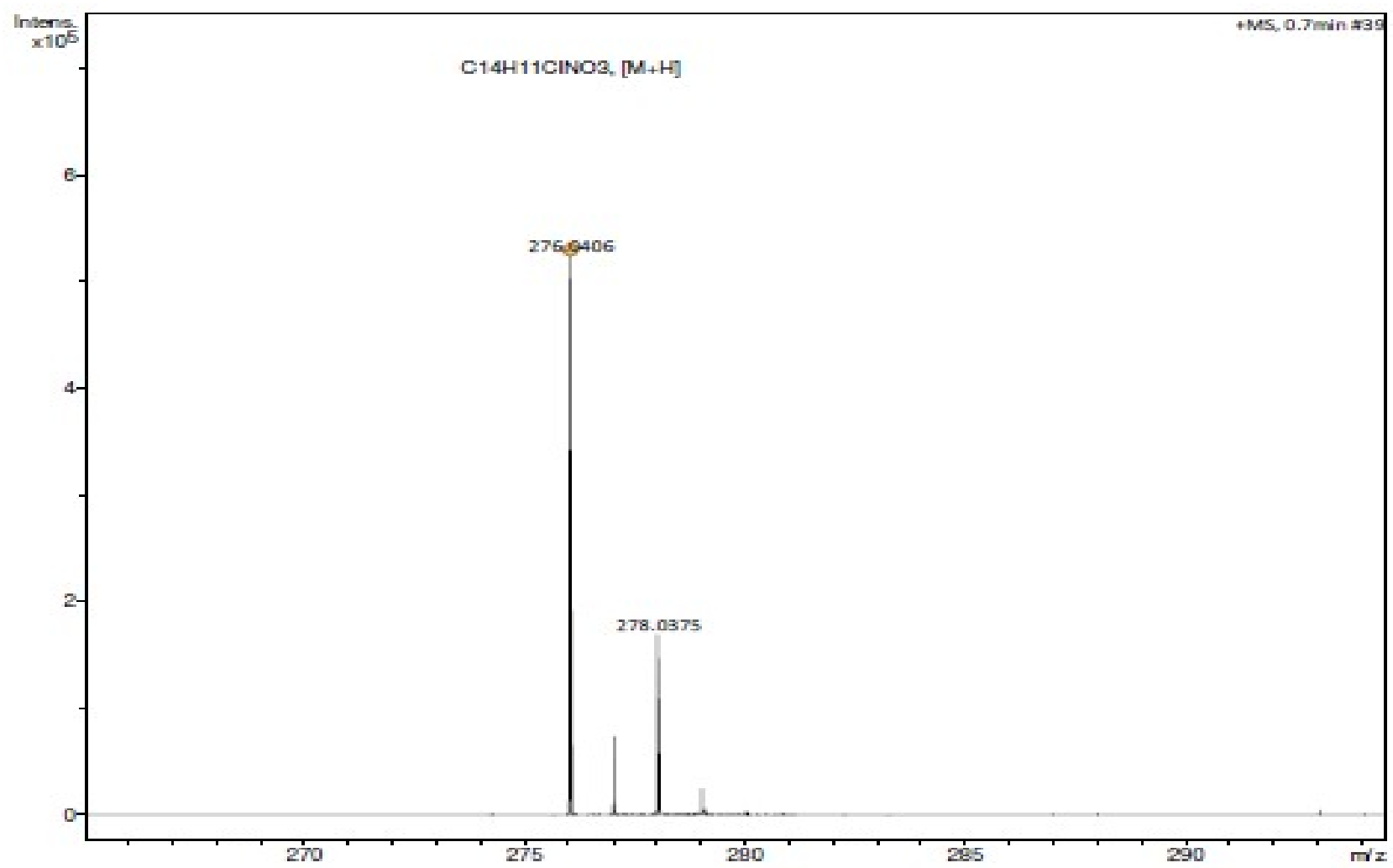

Meas. m/z	#	Ion Formula	Score	m/z	err [mDa]	err [ppm]	mSigma	rdb	e⁻ Conf	N-Rule	Adduct
276.040579	1	C14H11ClNO3	100.00	276.042197	1.6	5.9	11.1	9.5	even	ok	M+H

3a_RD4_01_12380.d

Bruker Compass DataAnalysis 4.2 printed: 4/9/2026 2:44:21 PM by: Nitin S. Kadam CIF Page 1 of 1

S5-3b IR

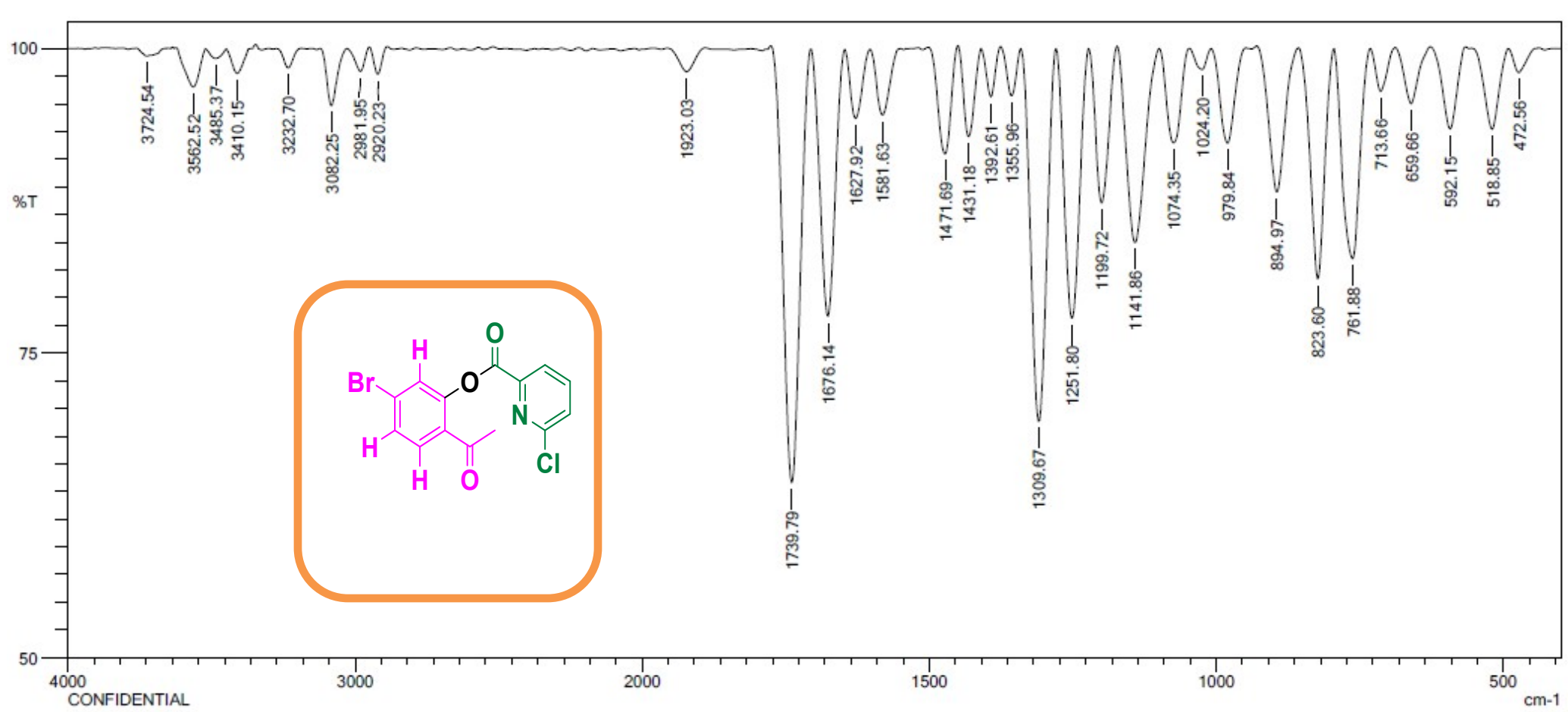

S6-3b ^{1}H NMR

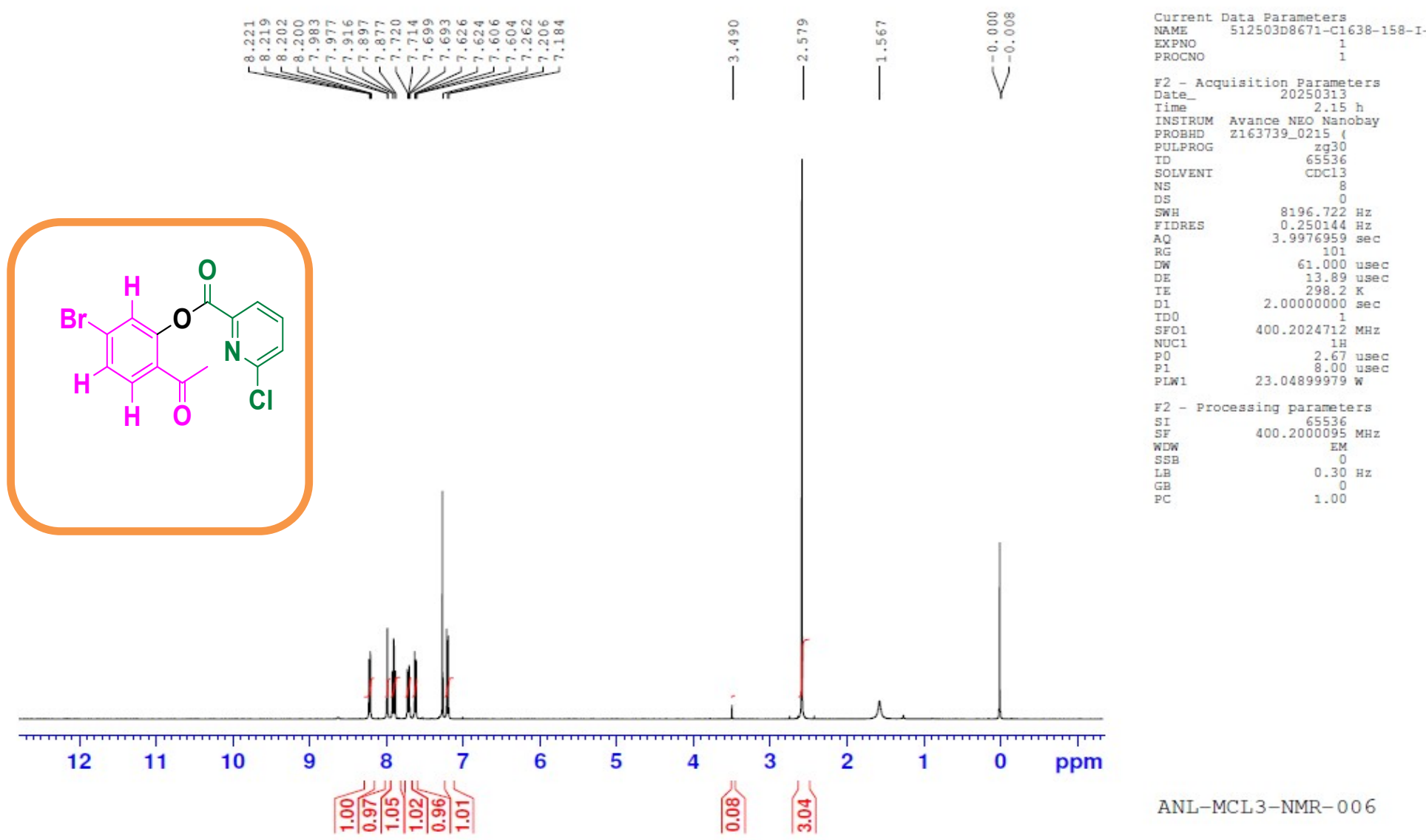

S7-3b ^{13}C NMR

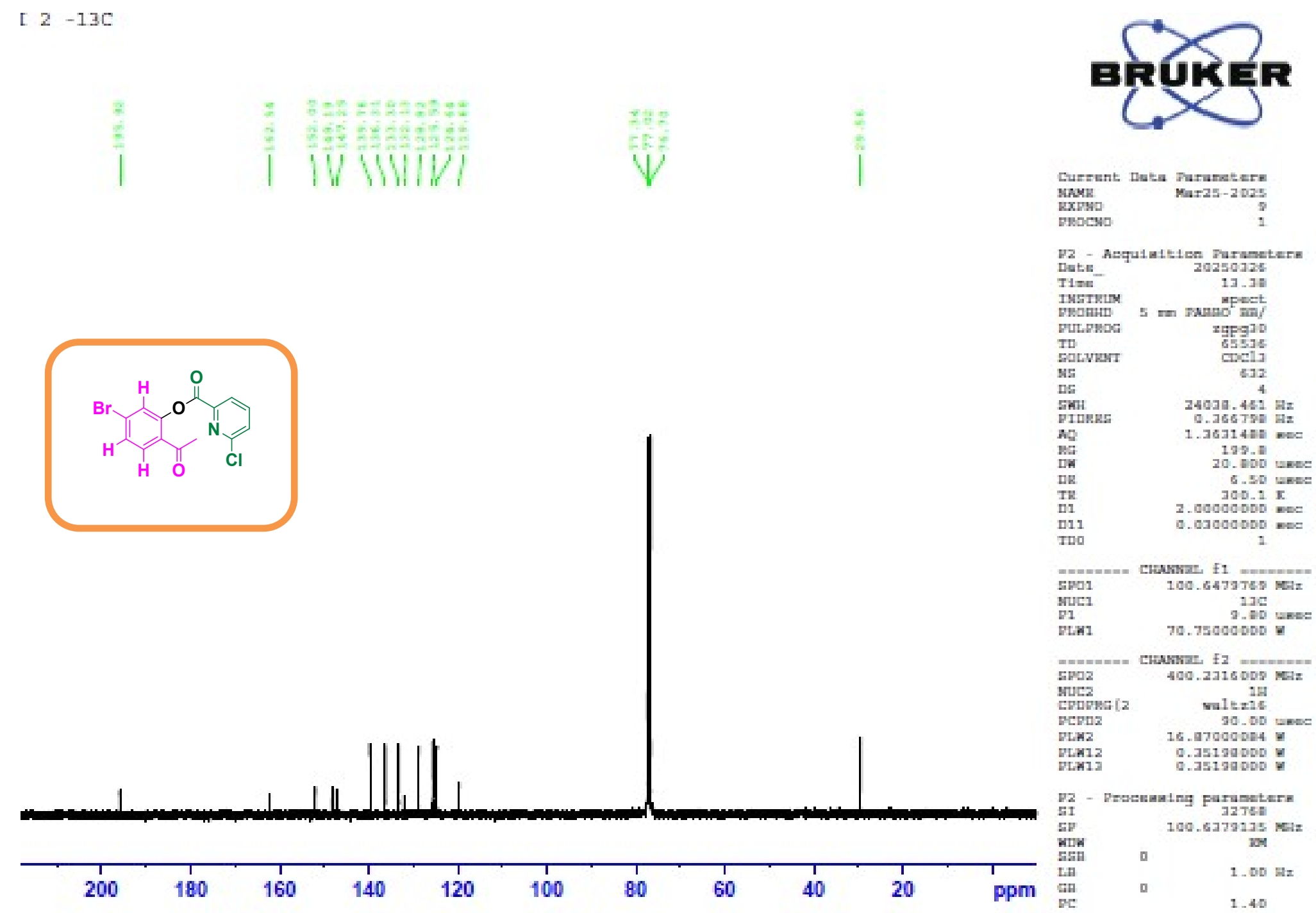

LC-MS 3b

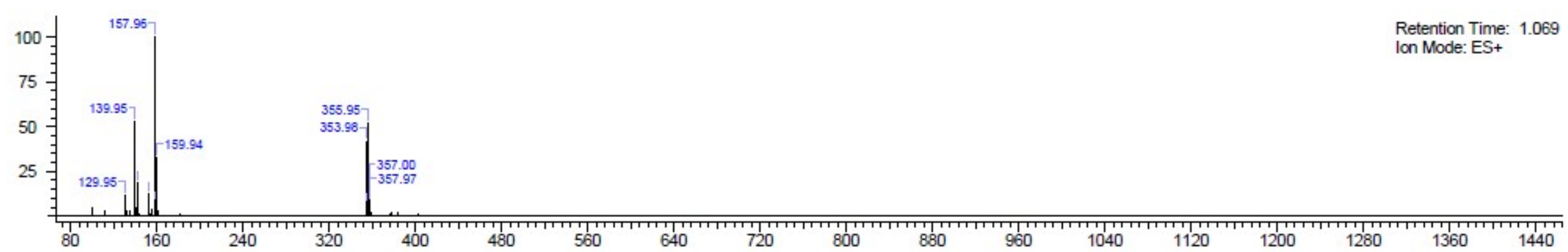

Retention Time: 1.069
Ion Mode: ES+
157.96
139.95
159.94
129.95
355.95
353.98
357.00
357.97

S8-3b HR-MS

Savitribai Phule Pune University - Central Instrumentation Facility

Analysis Info

Analysis Name	D:\Data\2026\APR\SPPU COLLEGE\RBNB COLLEGE SHRIRAMPUR\ARUNA SHINDE\3b_RD3_01_12379.d	Acquisition Date	4/9/2026 1:46:58 PM
Method	dlc_50-1200mz_pos_mode_3min_0.2ml_apr-2026.m	Analyst :	Nitin S. Kadam CIF
Sample Name	3b	Instrument	impact HD 1819696.00184

Acquisition Parameter

Source Type	ESI	Ion Polarity	Positive	Set Nebulizer	0.7 Bar
Focus	Active	Set Capillary	4000 V	Set Dry Heater	200 °C
Scan Begin	50 m/z	Set End Plate Offset	-500 V	Set Dry Gas	4.0 l/min
Scan End	1200 m/z	Set Charging Voltage	2000 V	Set Divert Valve	Source
		Set Corona	0 nA	Set APCI Heater	0 °C

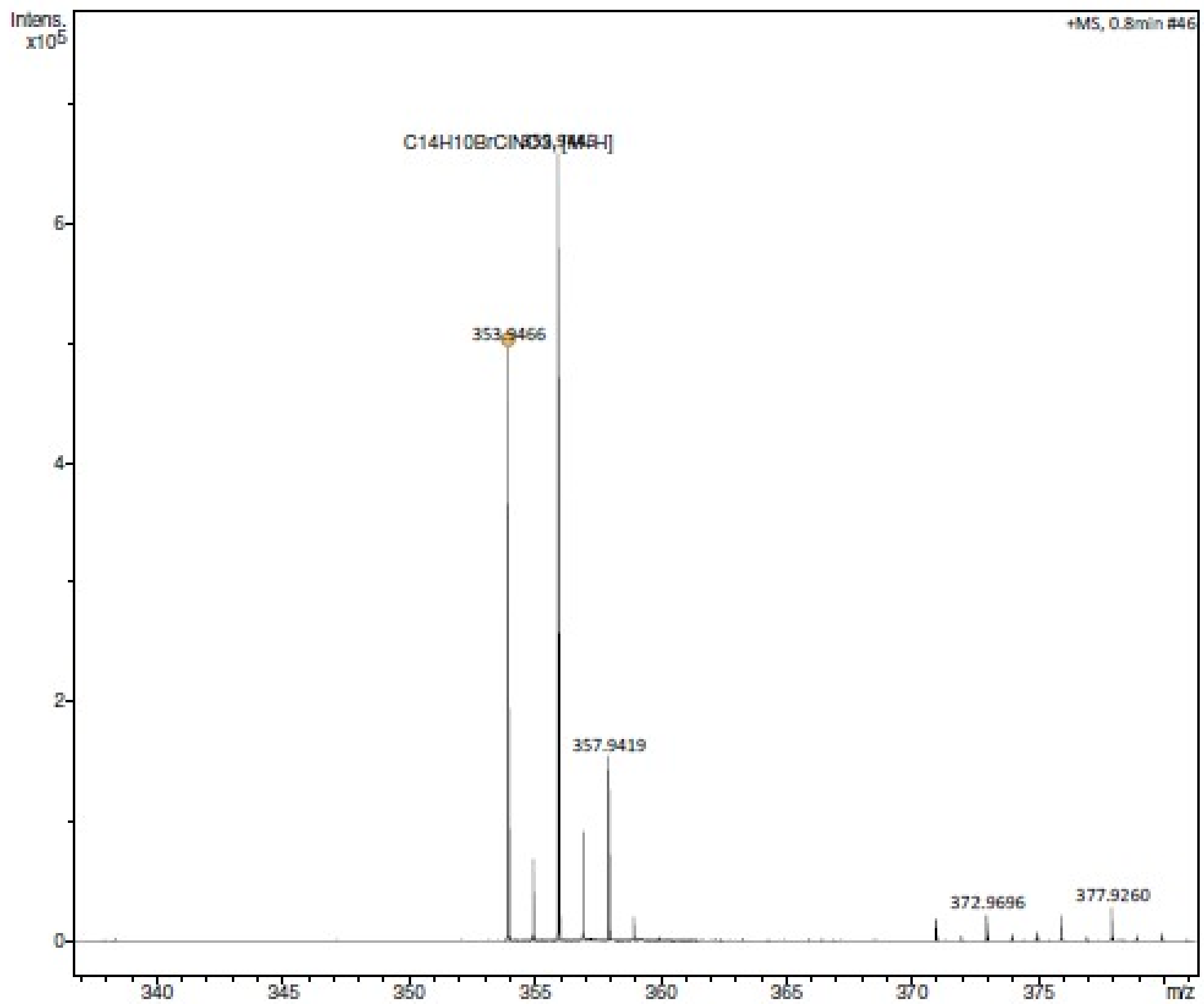

Meas. m/z	#	Ion Formula	Score	m/z	err [mDa]	err [ppm]	mSigma	rdb	e⁻ Conf	N-Rule	Adduct
353.946561	1	C14H10BrClNO3	100.00	353.952709	6.1	17.4	13.8	9.5	even	ok	M+H

S9-3c IR

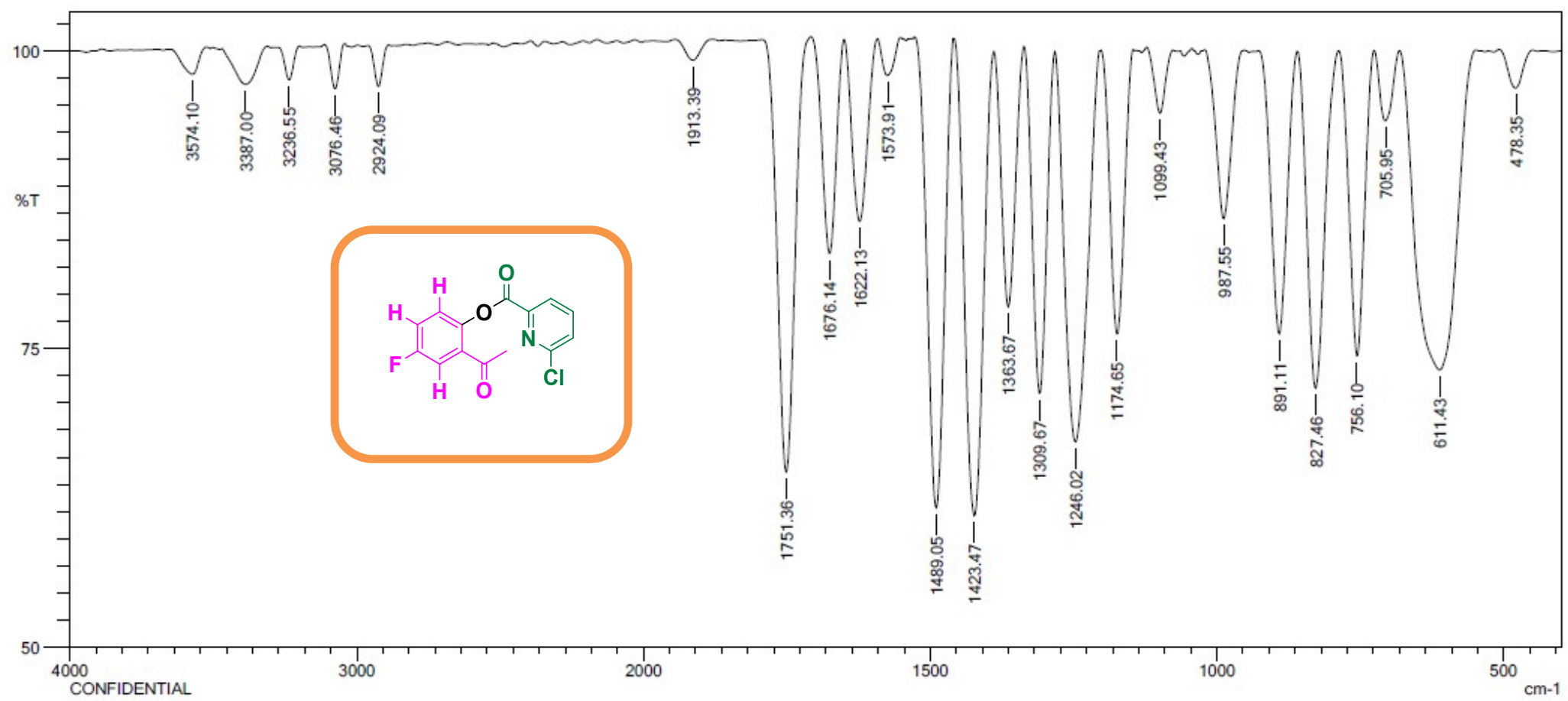

S10-3c ^{1}H NMR

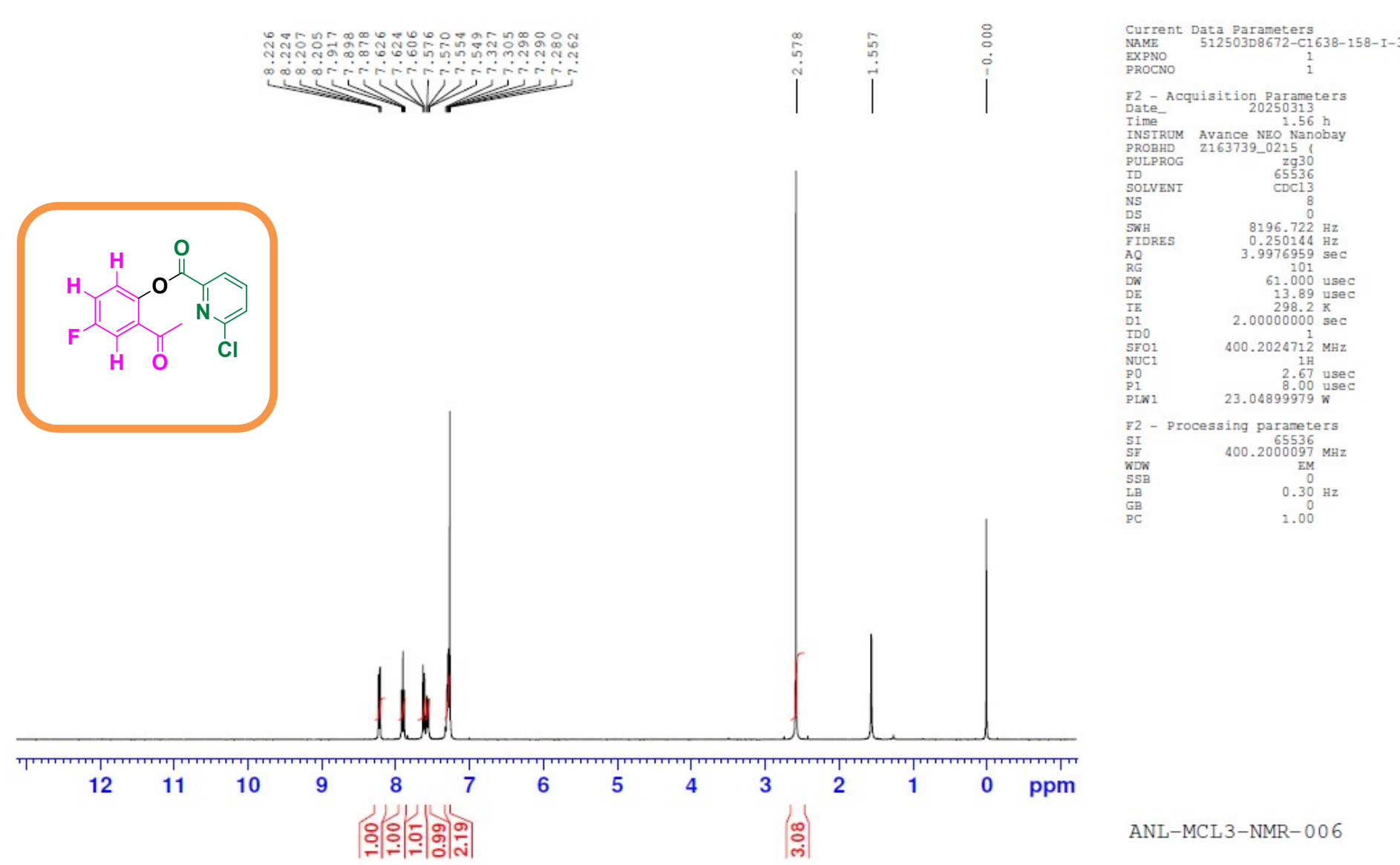

S11-3c ^{13}C NMR

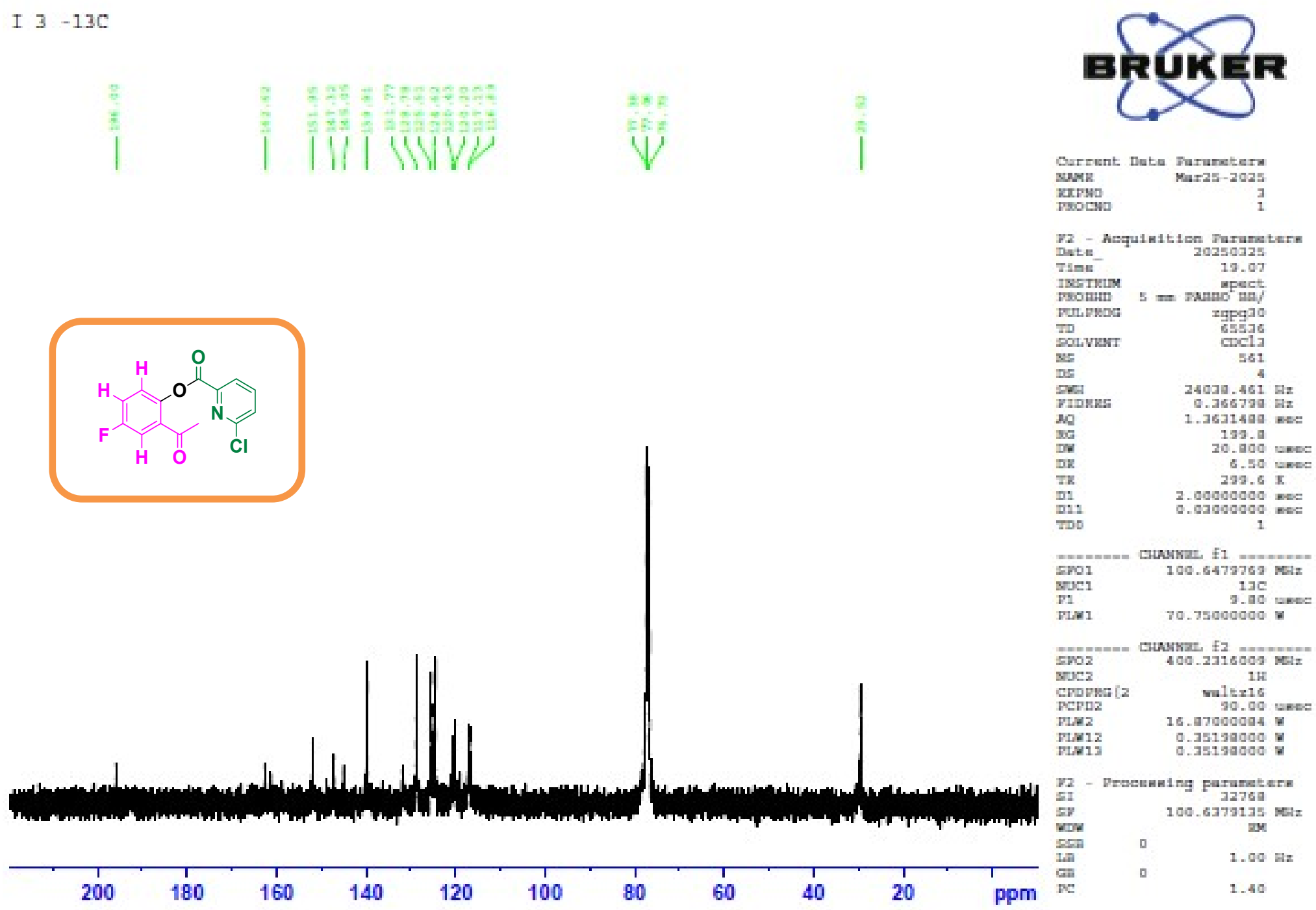

LC-MS 3c

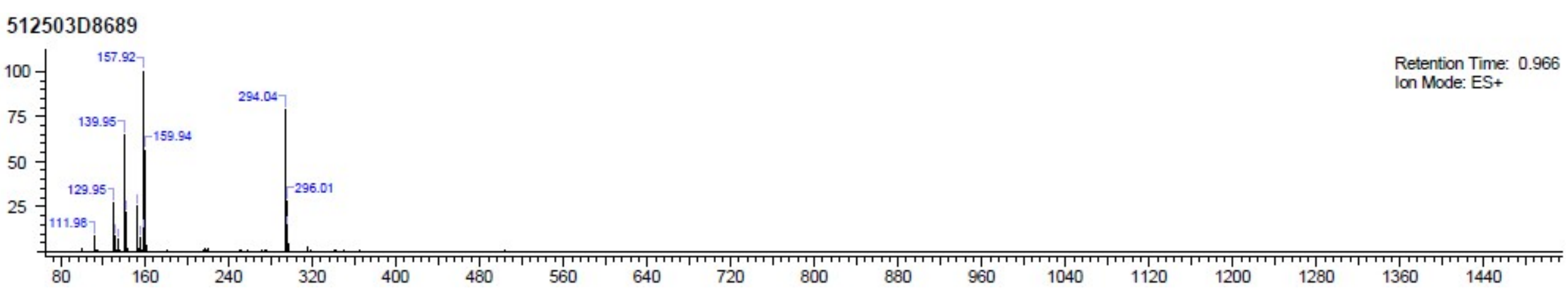

S12-3c HR-MS

Savitribai Phule Pune University - Central Instrumentation Facility

Analysis Info

Analysis Name	D:\Data\2026\APR\SPPU COLLEGE\RBNB COLLEGE SHRIRAMPUR\ARUNA SHINDE\3c_RD5_01_12381.d	Acquisition Date	4/9/2026 1:54:22 PM
Method	dlc_50-1200mz_pos_mode_3min_0.2ml_apr-2026.m	Analyst :	Nitin S. Kadam CIF
Sample Name	3c	Instrument	impact HD 1819696.00184

Acquisition Parameter

Source Type	ESI	Ion Polarity	Positive	Set Nebulizer	0.7 Bar
Focus	Active	Set Capillary	4000 V	Set Dry Heater	200 °C
Scan Begin	50 m/z	Set End Plate Offset	-500 V	Set Dry Gas	4.0 l/min
Scan End	1200 m/z	Set Charging Voltage	2000 V	Set Divert Valve	Source
		Set Corona	0 nA	Set APCI Heater	0 °C

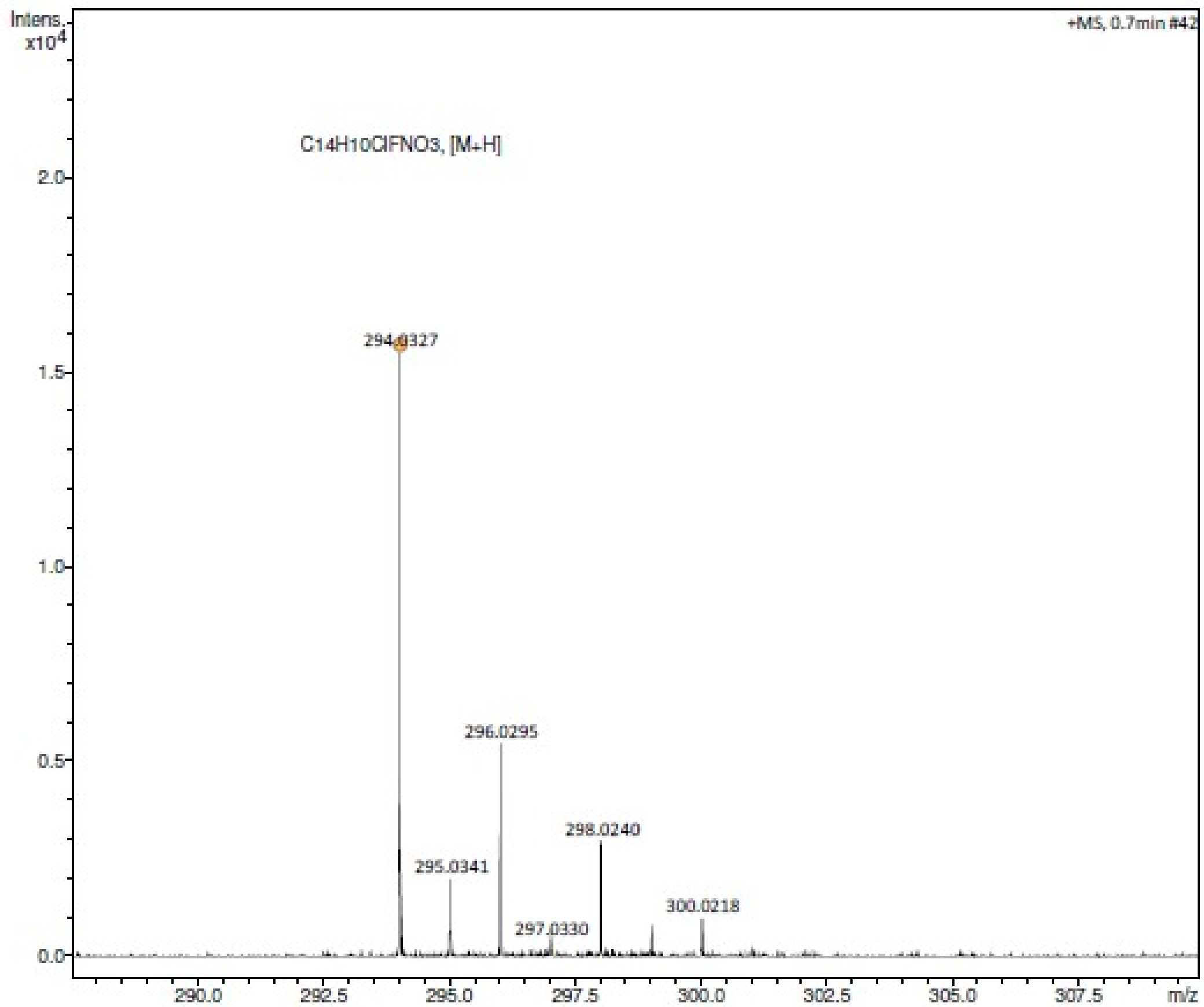

Meas. m/z	#	Ion Formula	Score	m/z	err [mDa]	err [ppm]	mSigma	rdb	e⁻ Conf	N-Rule	Adduct
294.032656	1	C14H10ClFNO3	100.00	294.032776	0.1	0.4	21.5	9.5	even	ok	M+H

S13-4a IR

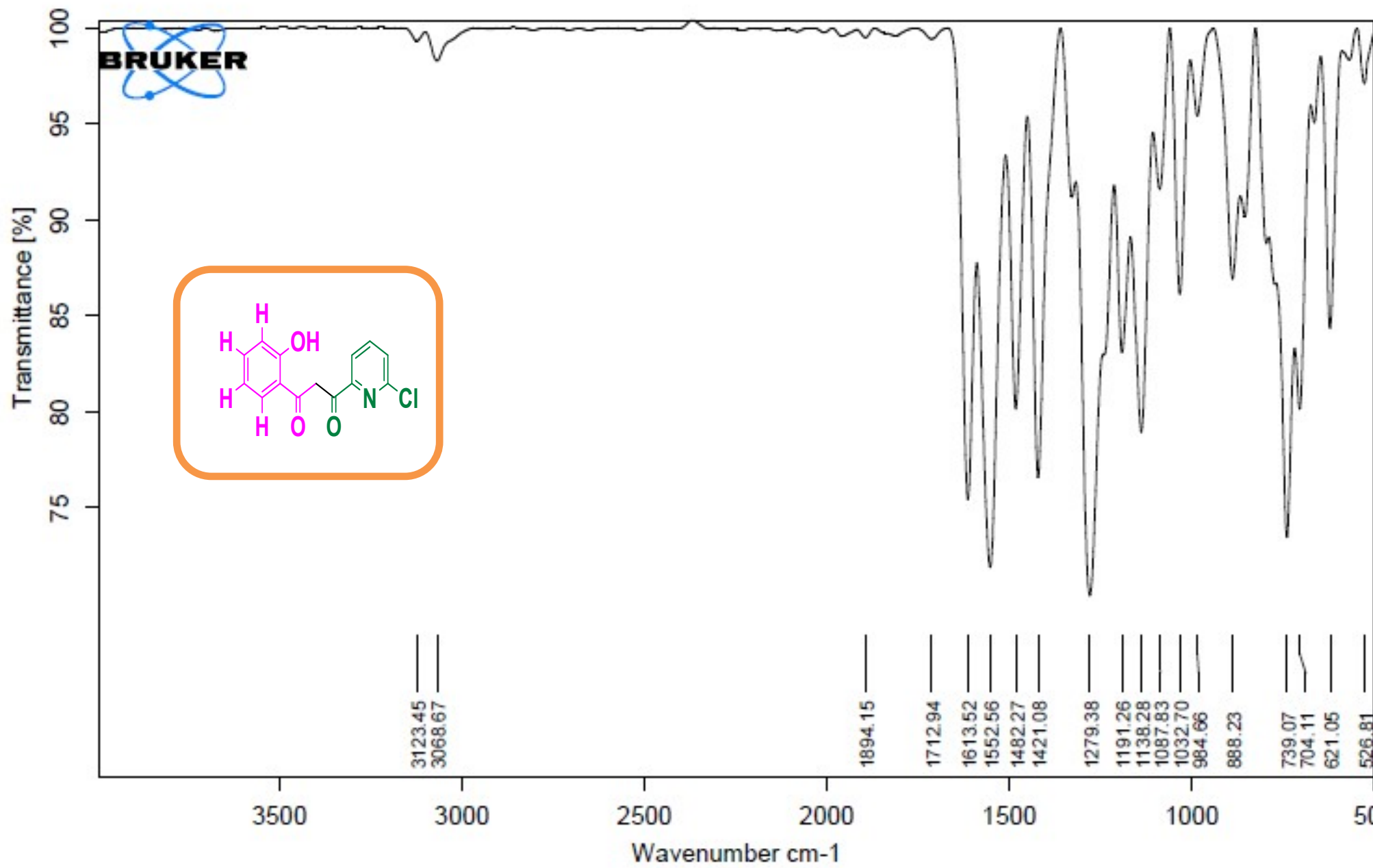

S14 - 4a[1]H NMR

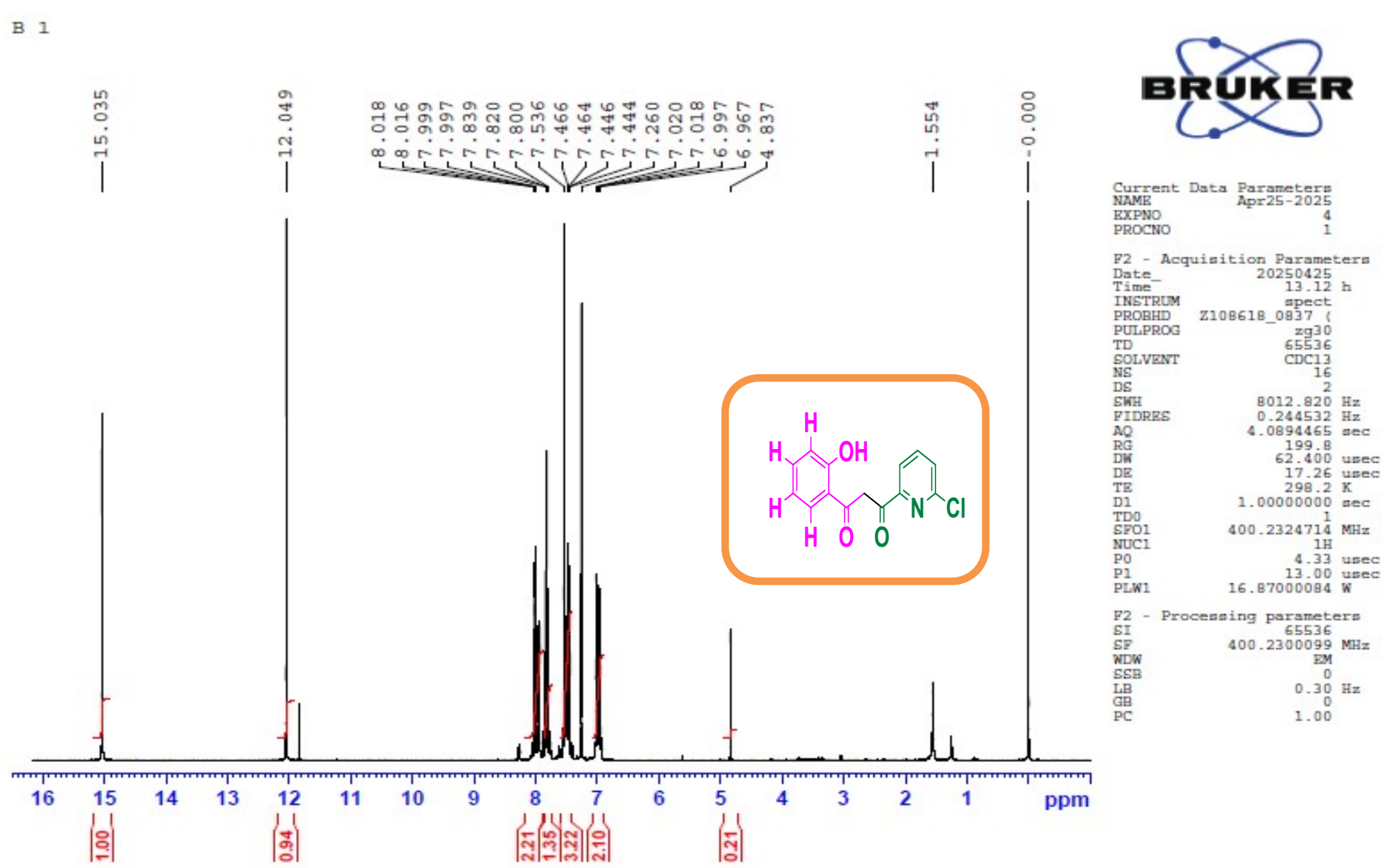

S15 - 4a MS

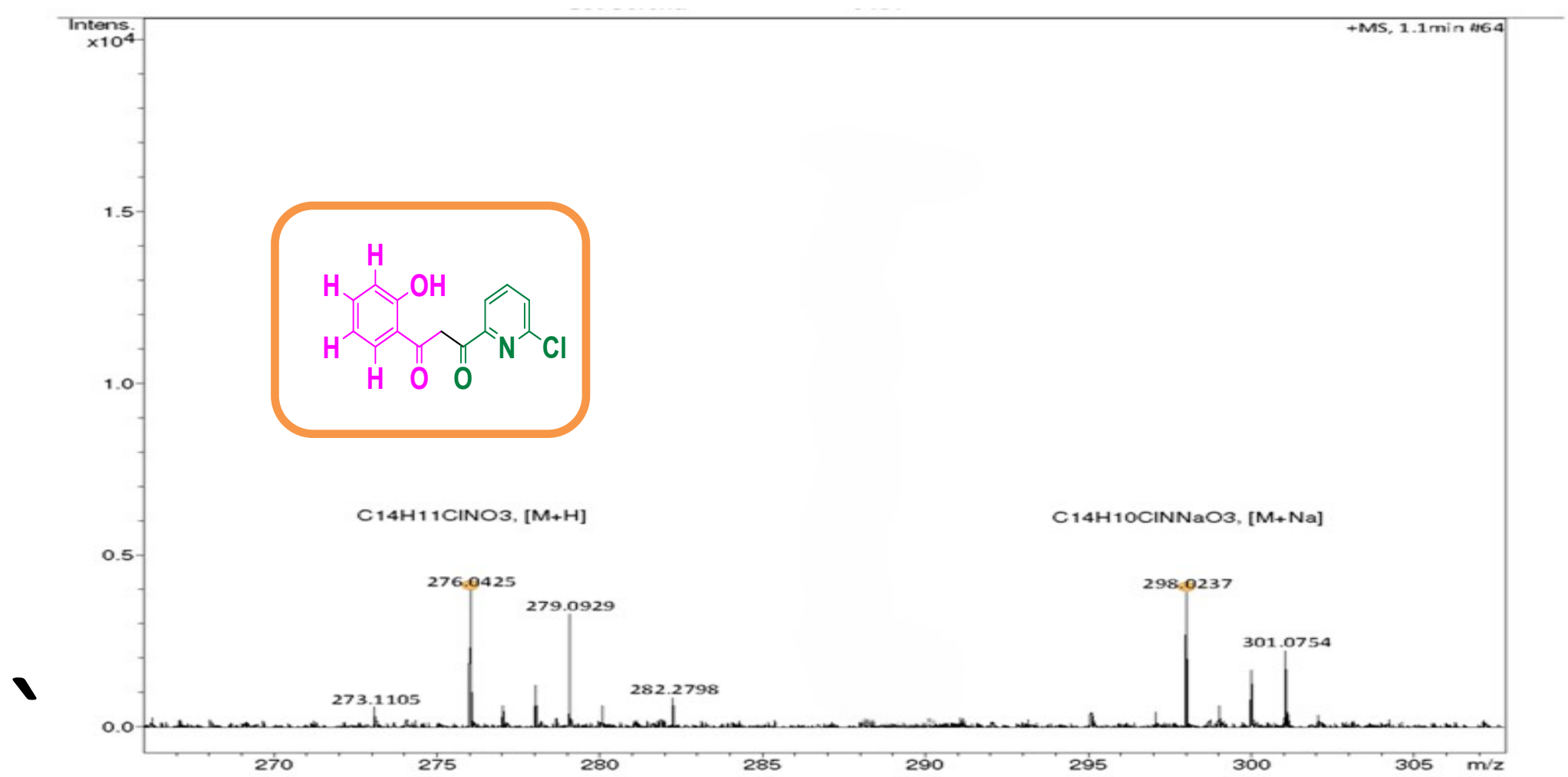

S16 - 4b IR

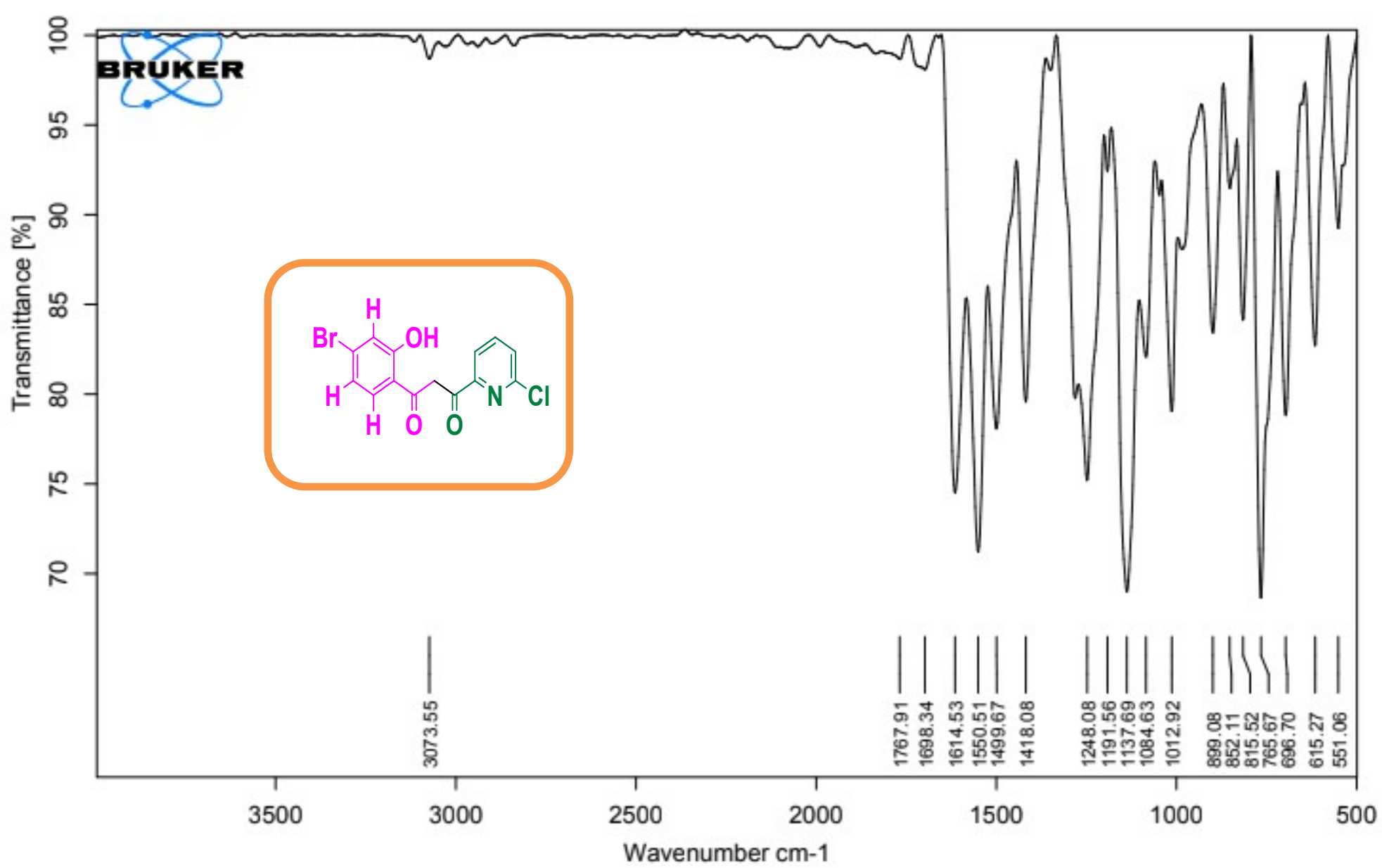

S17 - 4b ^{1}H NMR

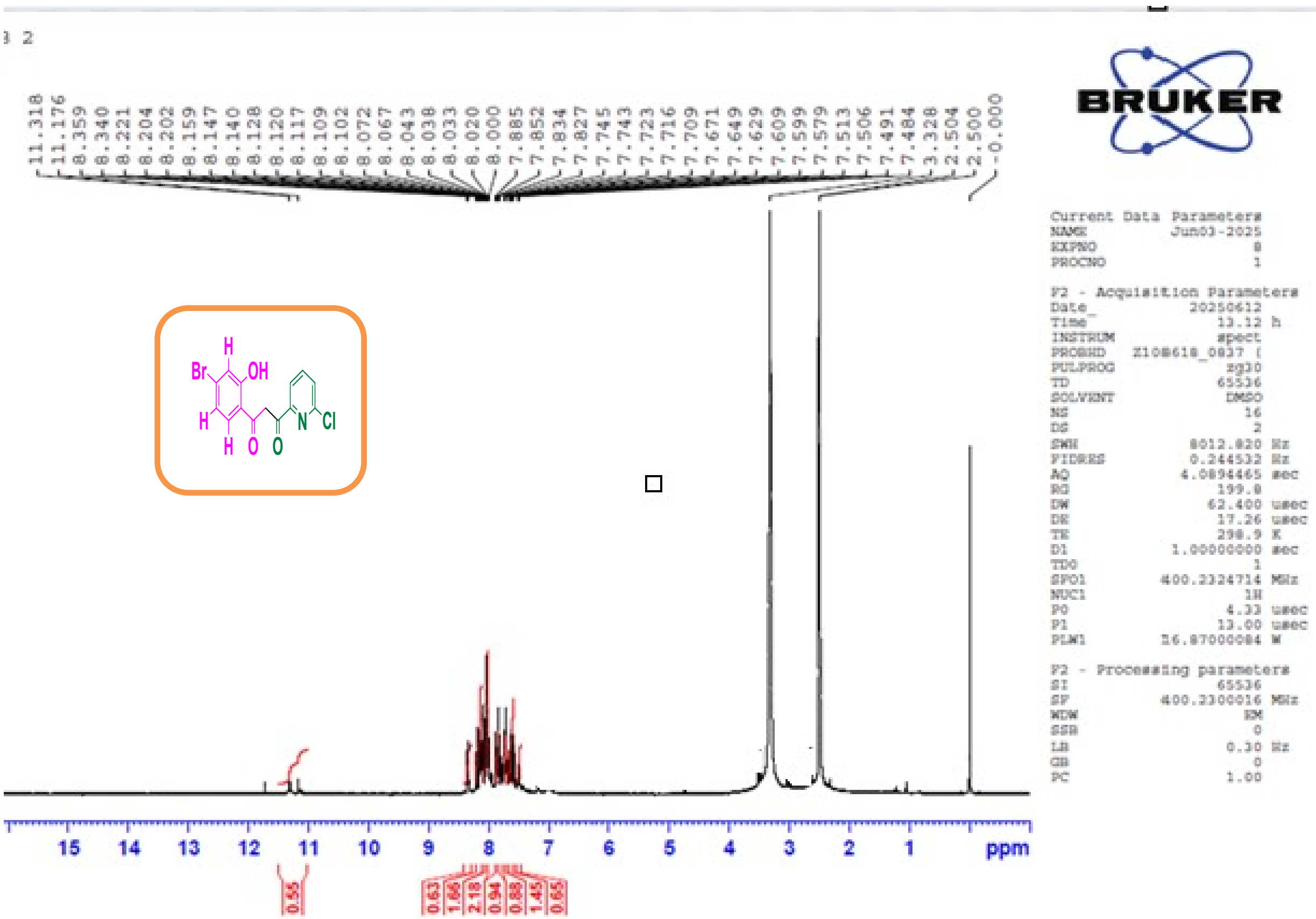

3 2
11.318
11.176
8.359
8.340
8.221
8.204
8.202
8.159
8.147
8.140
8.128
8.120
8.117
8.109
8.102
8.072
8.067
8.043
8.038
8.033
8.020
8.000
7.885
7.852
7.834
7.827
7.745
7.743
7.723
7.716
7.709
7.671
7.649
7.629
7.609
7.599
7.579
7.513
7.506
7.491
7.484
3.328
2.504
2.500
-0.000
Br
H
OH
H
H
O
O
N
Cl
15
14
13
12
11
10
9
8
7
6
5
4
3
2
1
ppm
0.55
0.63
1.66
2.18
0.94
0.88
1.45
0.65
Current Data Parameters
NAME Jun03-2025
EXPNO 8
PROCNO 1
F2 - Acquisition Parameters
Date_ 20250612
Time 13.12 h
INSTRUM spect
PROBHD Z108618_0837 (
PULPROG zg30
TD 65536
SOLVENT DMSO
NS 16
DS 2
SWH 8012.820 Hz
FIDRES 0.244532 Hz
AQ 4.0894465 sec
RG 199.8
DW 62.400 usec
DE 17.26 usec
TE 298.9 K
D1 1.00000000 sec
TD0 1
SFO1 400.2324714 MHz
NUC1 1H
P0 4.33 usec
P1 13.00 usec
PLW1 16.87000084 W
F2 - Processing parameters
SI 65536
SF 400.2300016 MHz
WDW EM
SSB 0
LB 0.30 Hz
GB 0
PC 1.00

S18 - 4b HRMS

Savitribai Phule Pune University - Central Instrumentation Facility

Analysis Info

Analysis Name	D:\Data\2025\SEPT\SPPU COLLEGE\MODERN COLLEGE,GANESHKHIND\ARUNA SHINDE\3 b_RD2_01_10798.d	Acquisition Date	9/15/2025 5:18:44 PM
Method	dlc_50-1200mz_pos_mode_7min_0.2ml_jun2025.m	Analyst :	Nitin S. Kadam CIF
Sample Name	3 b	Instrument	impact HD 1819696.00184

Acquisition Parameter

Source Type	ESI	Ion Polarity	Positive	Set Nebulizer	0.7 Bar
Focus	Active	Set Capillary	4000 V	Set Dry Heater	200 °C
Scan Begin	50 m/z	Set End Plate Offset	-500 V	Set Dry Gas	4.0 l/min
Scan End	1200 m/z	Set Charging Voltage	2000 V	Set Divert Valve	Source
		Set Corona	0 nA	Set APCI Heater	0 °C

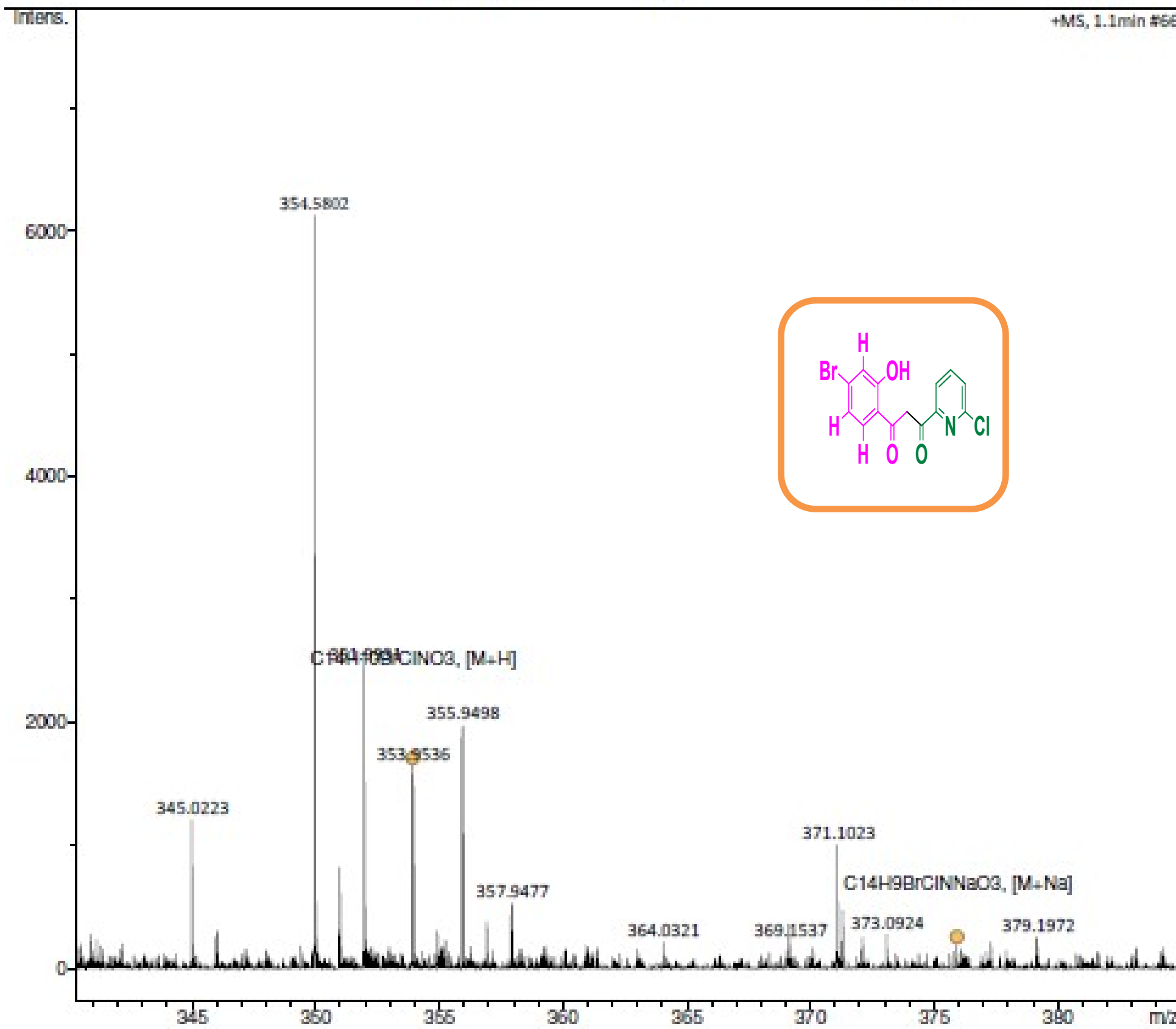

Meas. m/z	#	Ion Formula	Score	m/z	err [mDa]	err [ppm]	mSigma	rdb	e⁻ Conf	N-Rule	Adduct
353.953646	1	C14H10BrClNO3	100.00	353.952709	-0.9	-2.6	41.0	9.5	even	ok	M+H
375.935090	1	C14H9BrClNNaO3	100.00	375.934654	-0.4	-1.2	190.1	9.5	even	ok	M+Na

3 b_RD2_01_10798.d

Bruker Compass DataAnalysis 4.2 printed: 9/16/2025 4:11:07 PM by: Nitin S. Kadam CIF Page 1 of 1

S19 - 4c IR

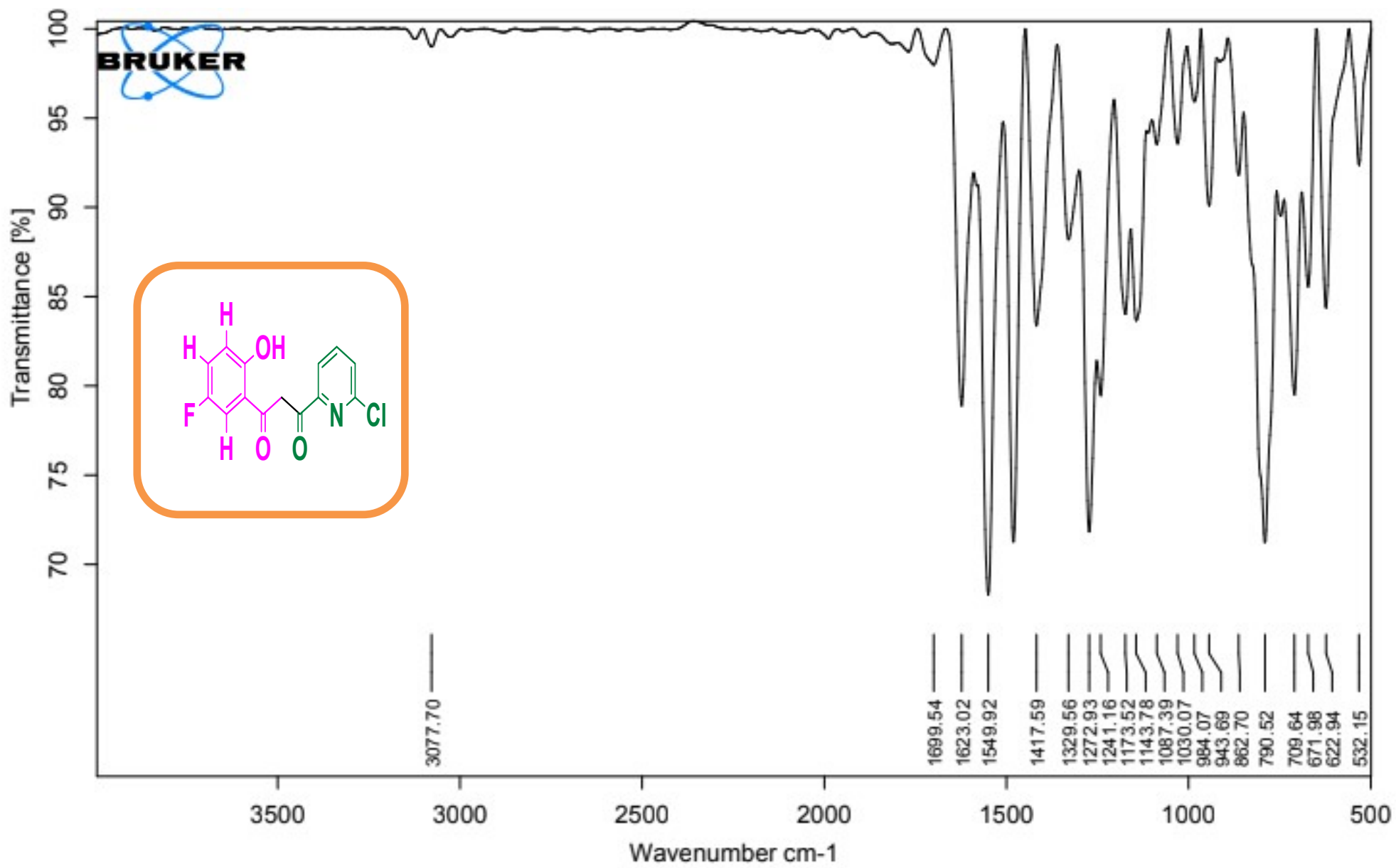

S20 - 4c ^{1}H NMR

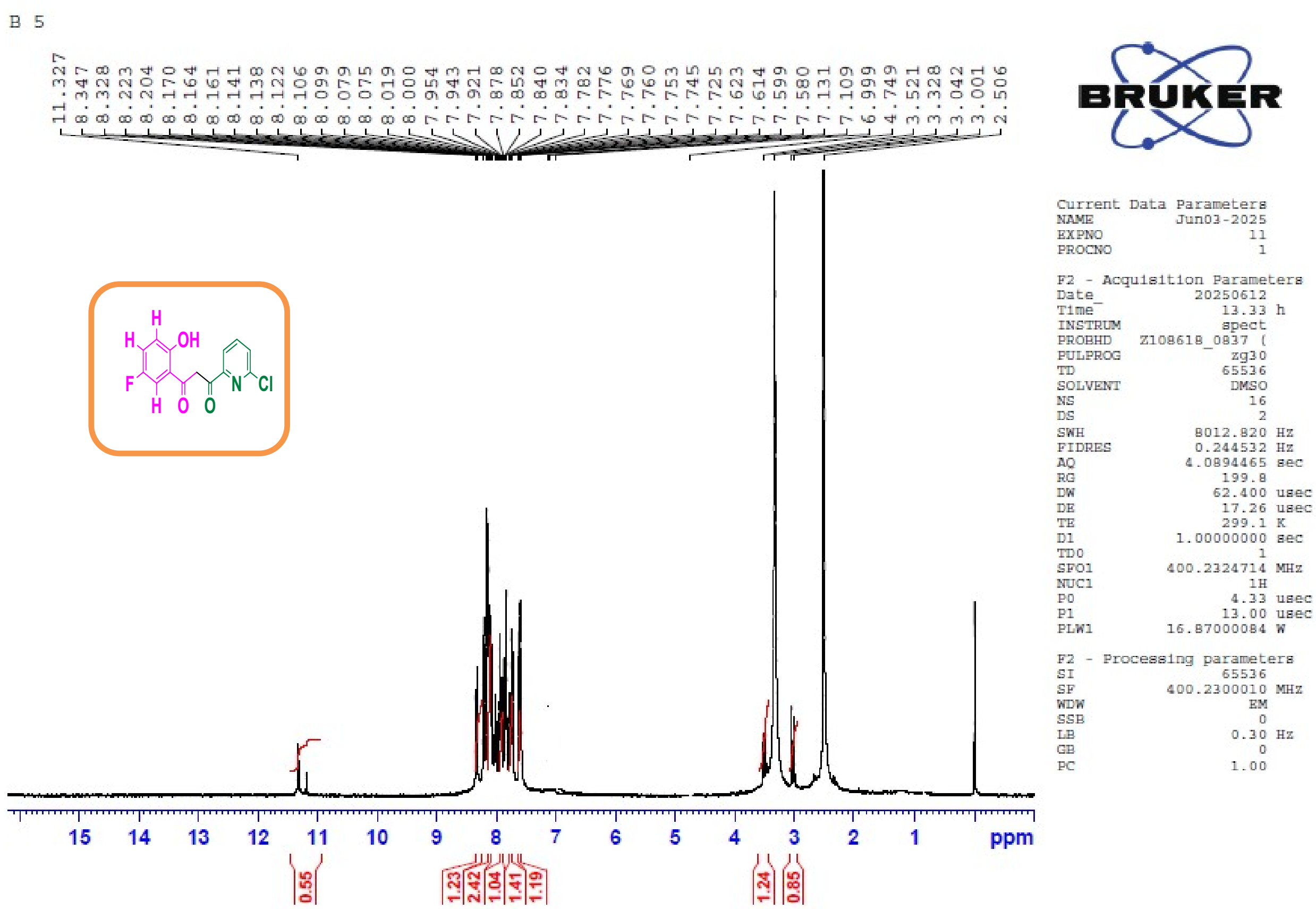

S21 - 4c HRMS

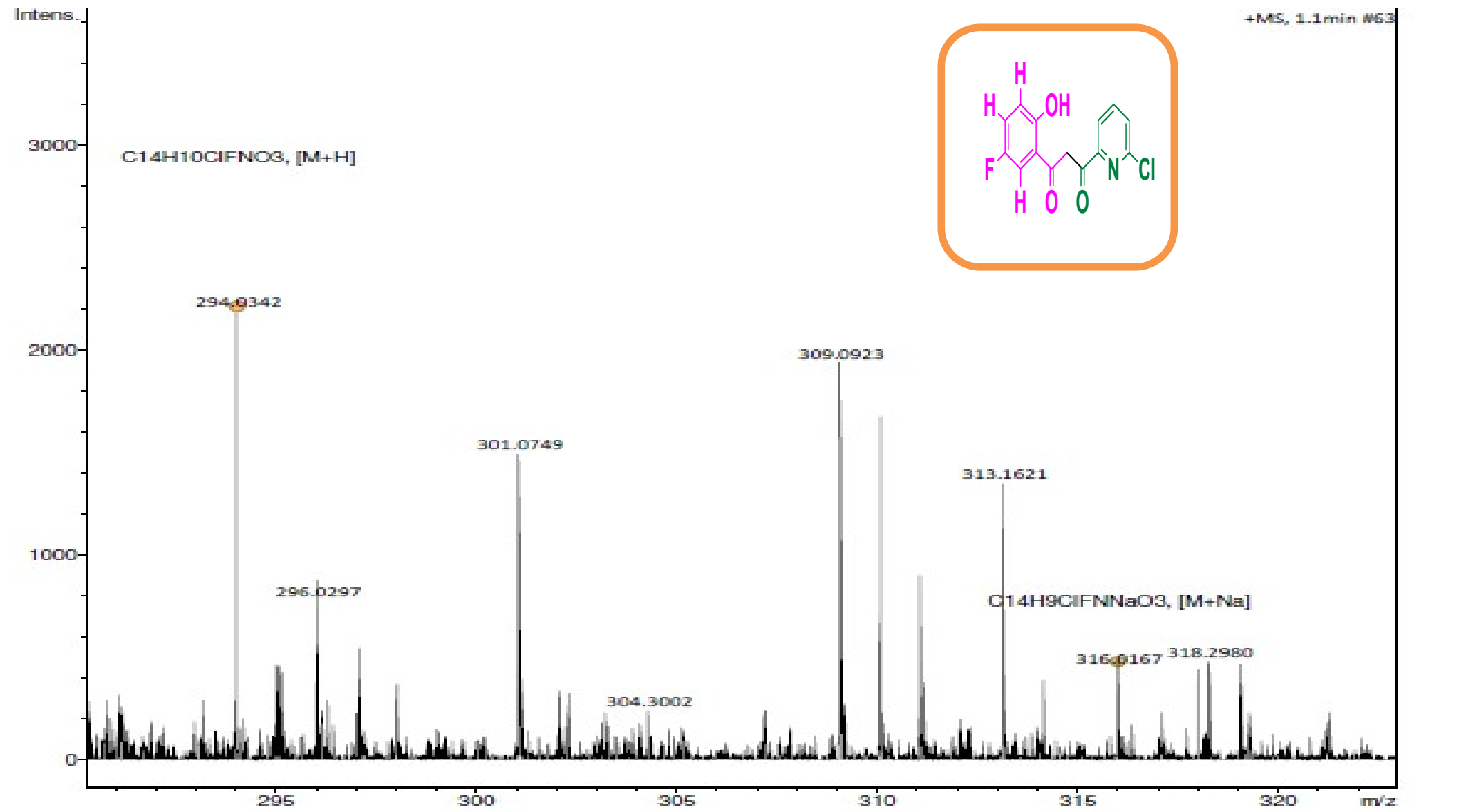

S22 - 5a IR

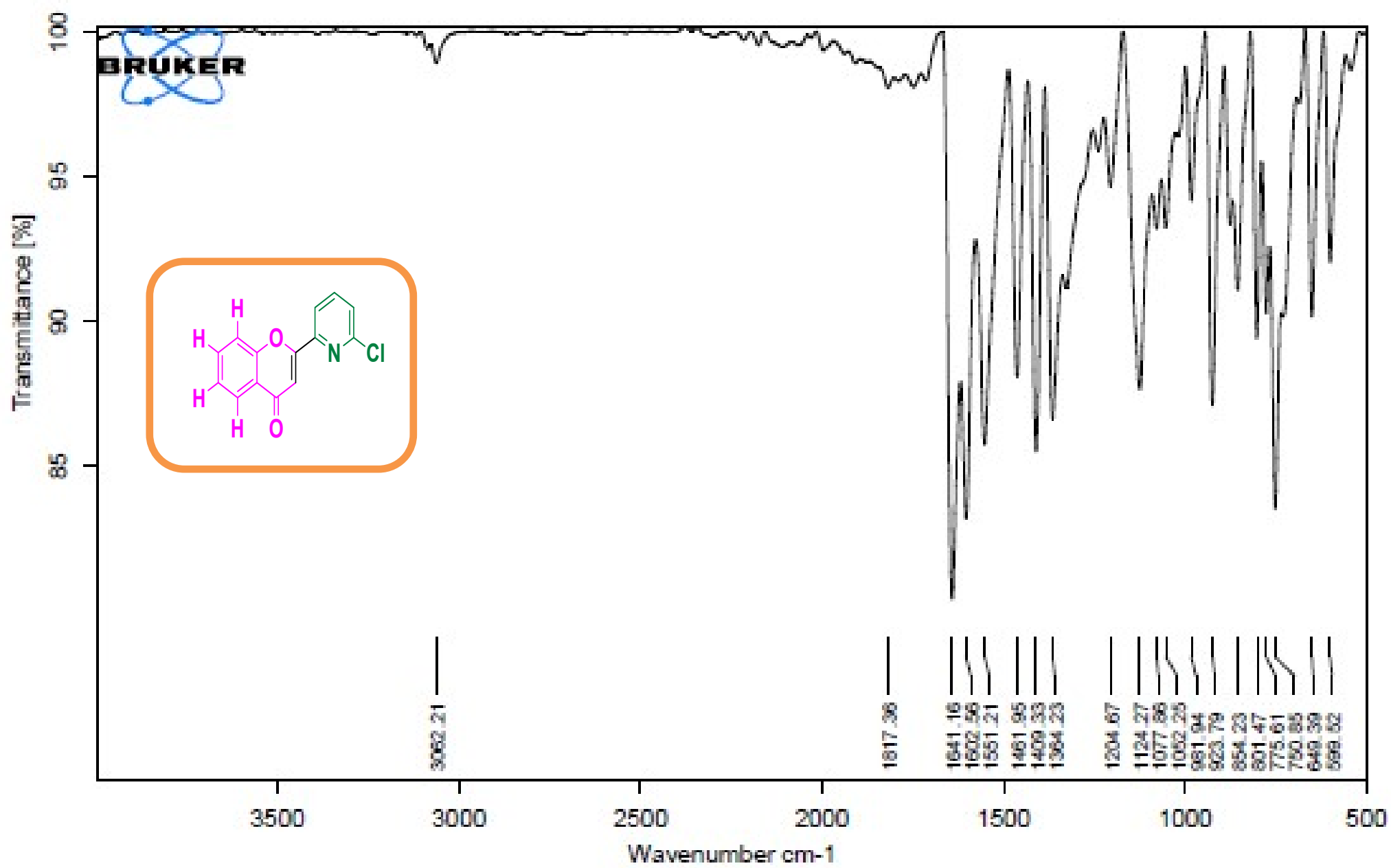

S23 - 5a ^{1}H NMR

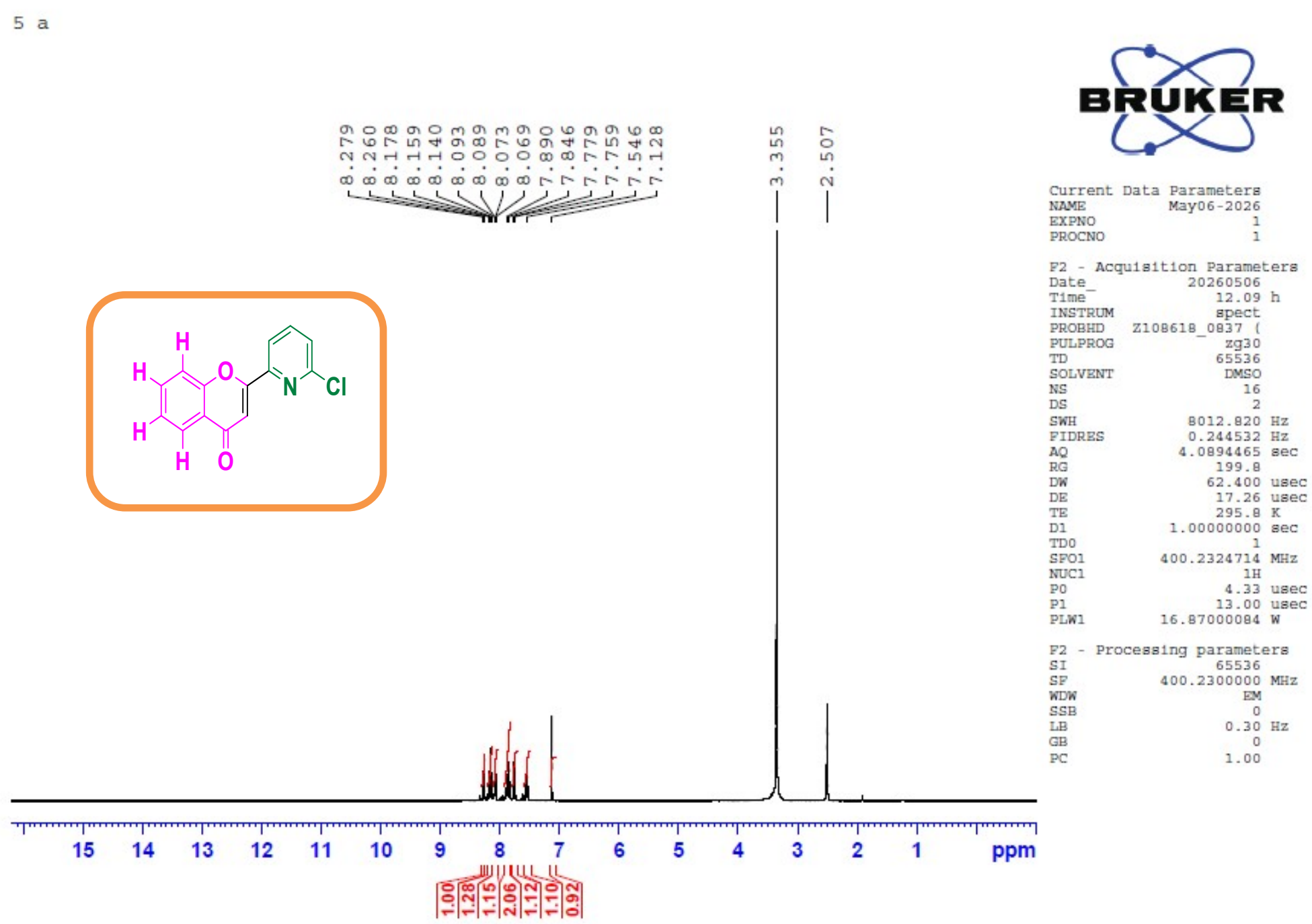

S24 - 5a ^{13}C NMR

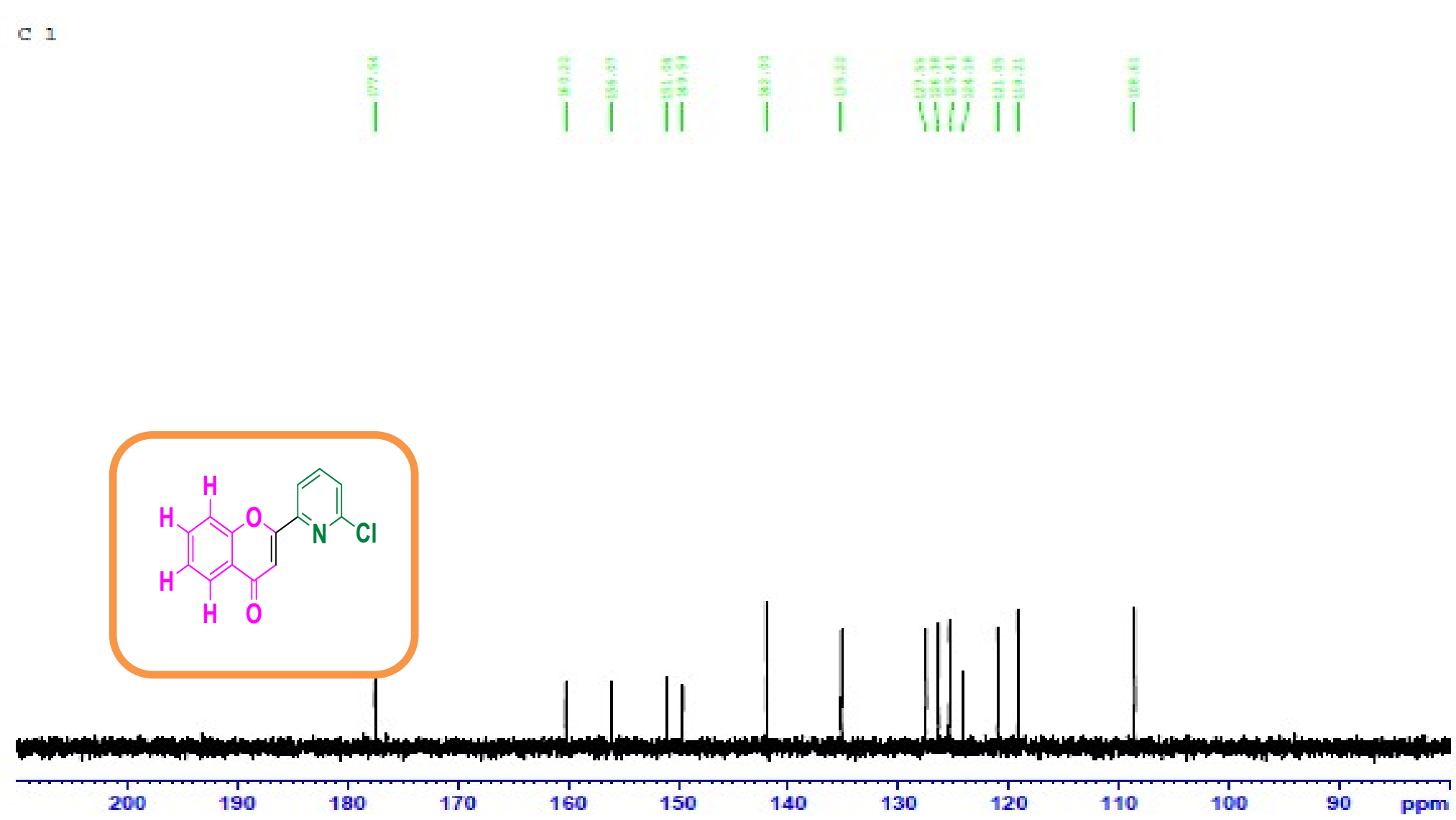

S25 - 5a HRMS

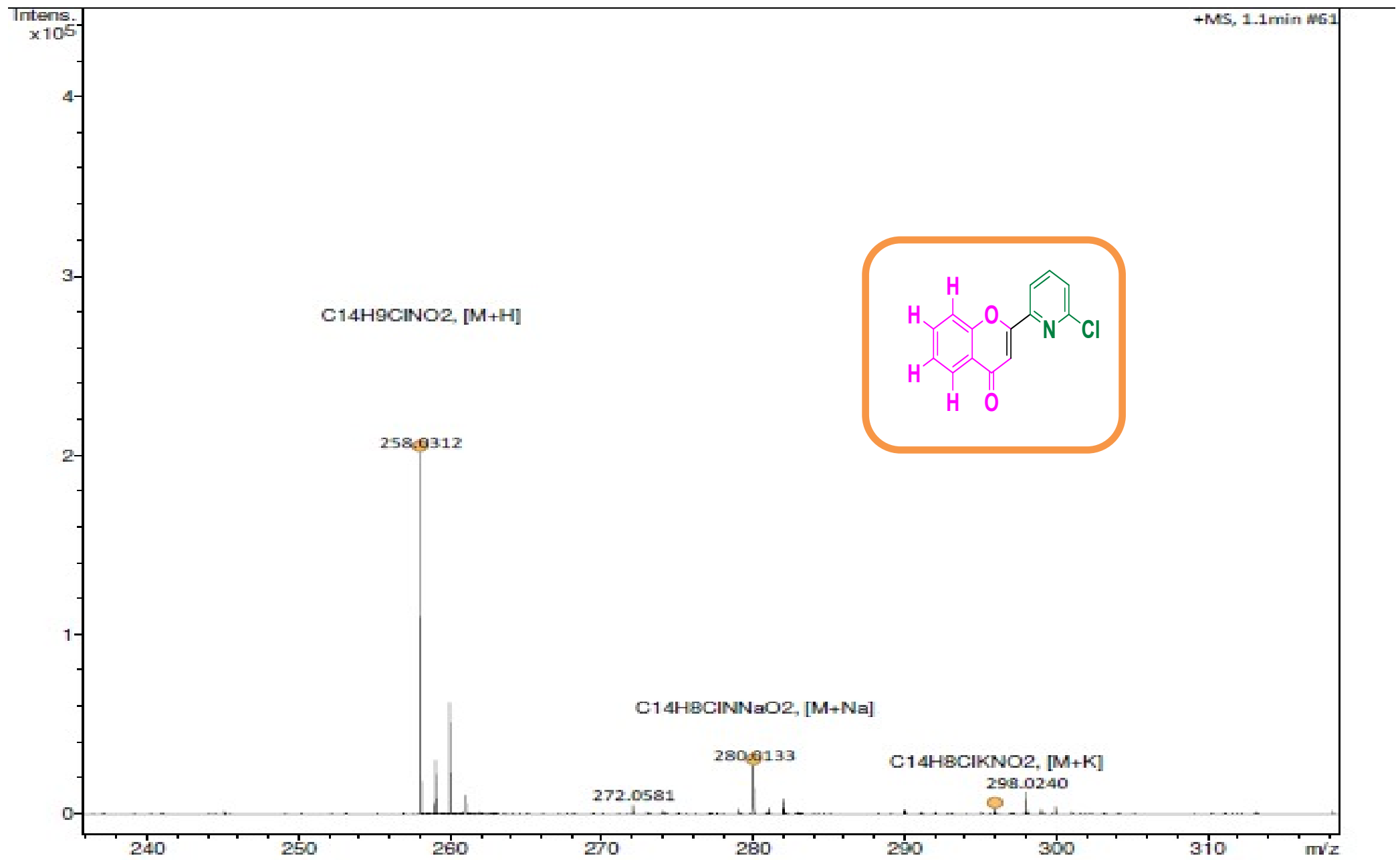

S26 - 5b IR

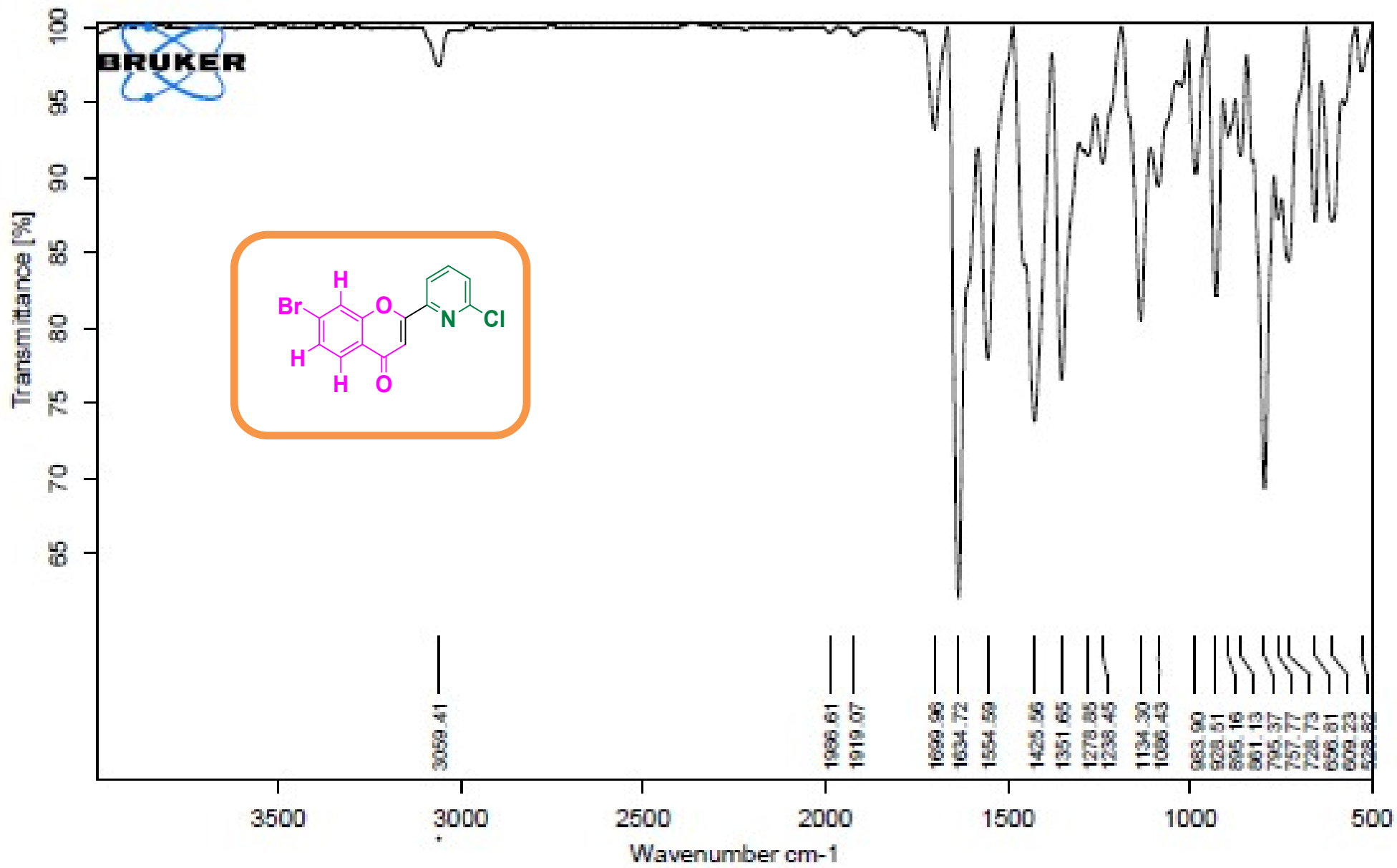

S27 - 5b ^{1}H NMR

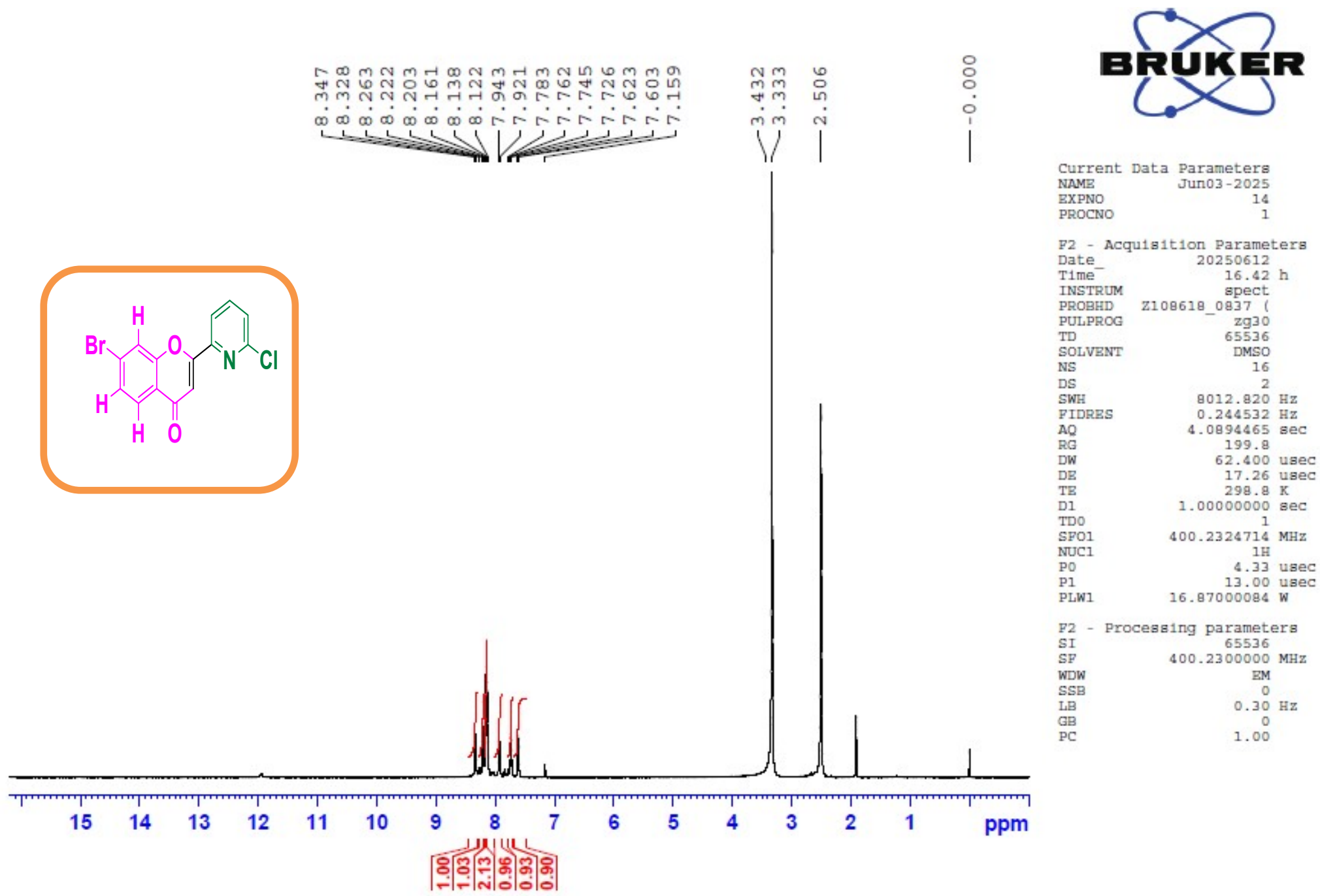

S27 - 5b ^{1}H NMR Splitting pattern spectra

C 5

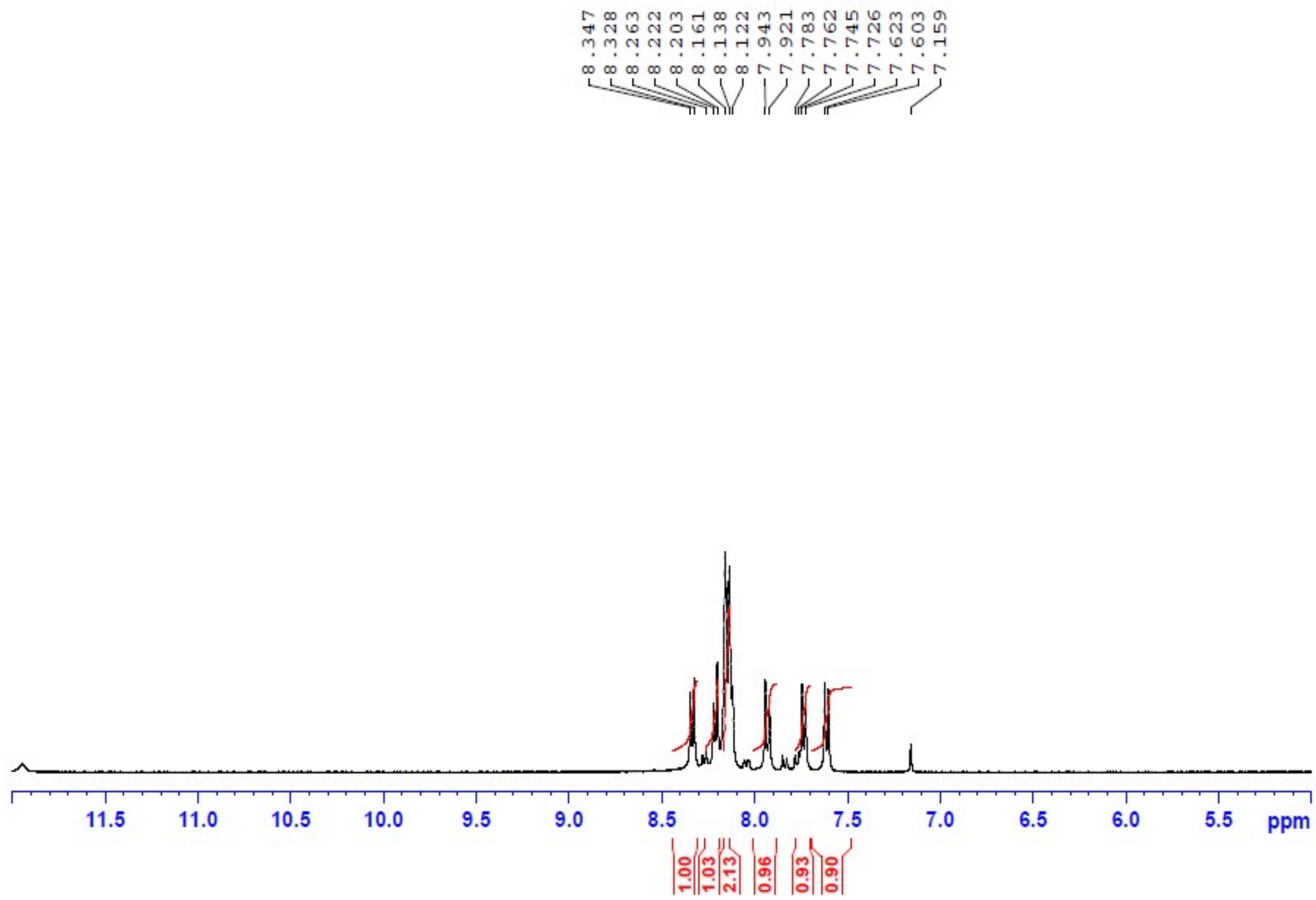
8.347
8.328
8.263
8.222
8.203
8.161
8.138
8.122
7.943
7.921
7.783
7.762
7.745
7.726
7.623
7.603
7.159
11.5
11.0
10.5
10.0
9.5
9.0
8.5
8.0
7.5
7.0
6.5
6.0
5.5
ppm
1.00
1.03
2.13
0.96
0.93
0.90

S28 - 5b ^{13}C NMR

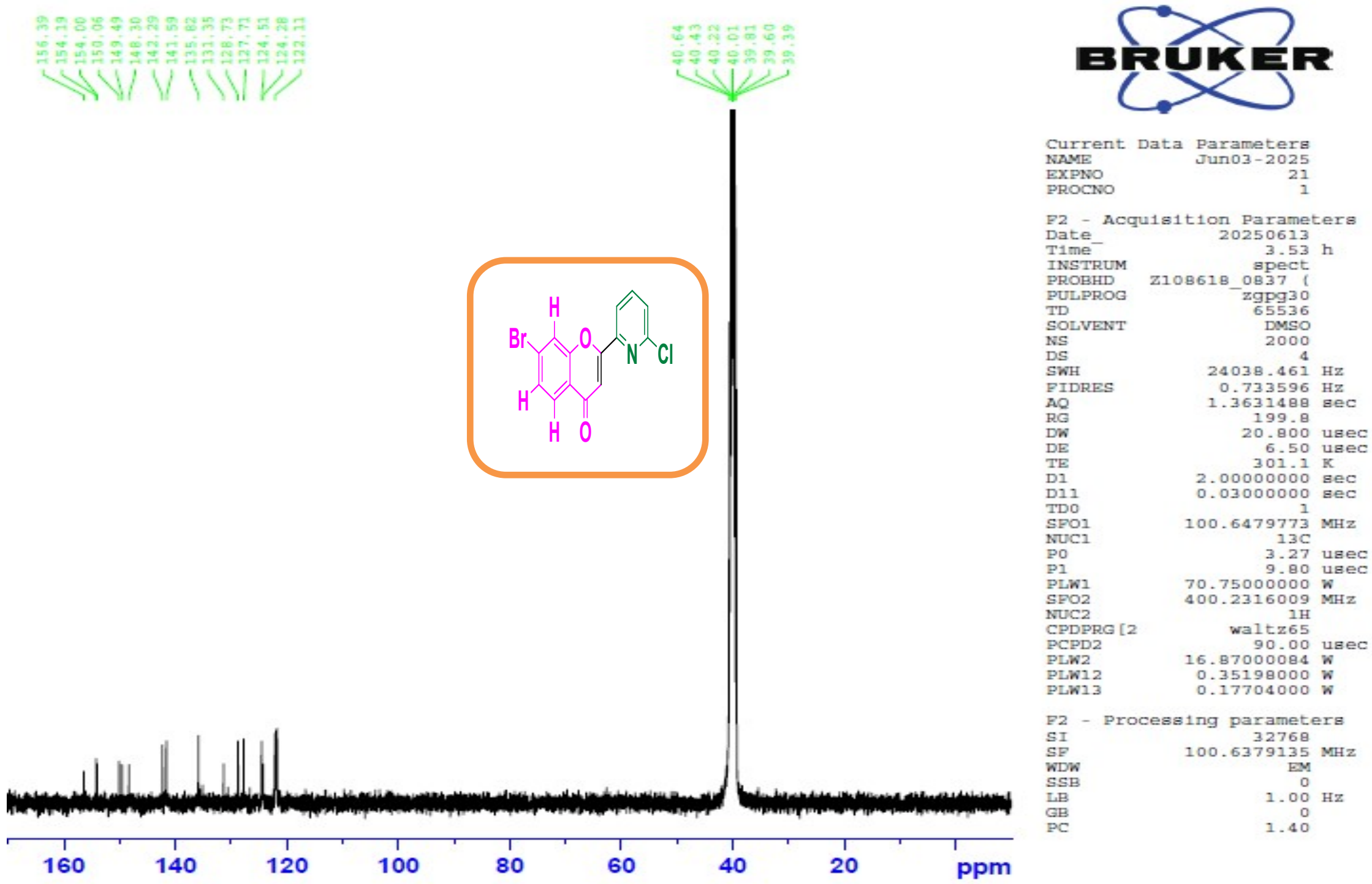

S29 - 5b HRMS

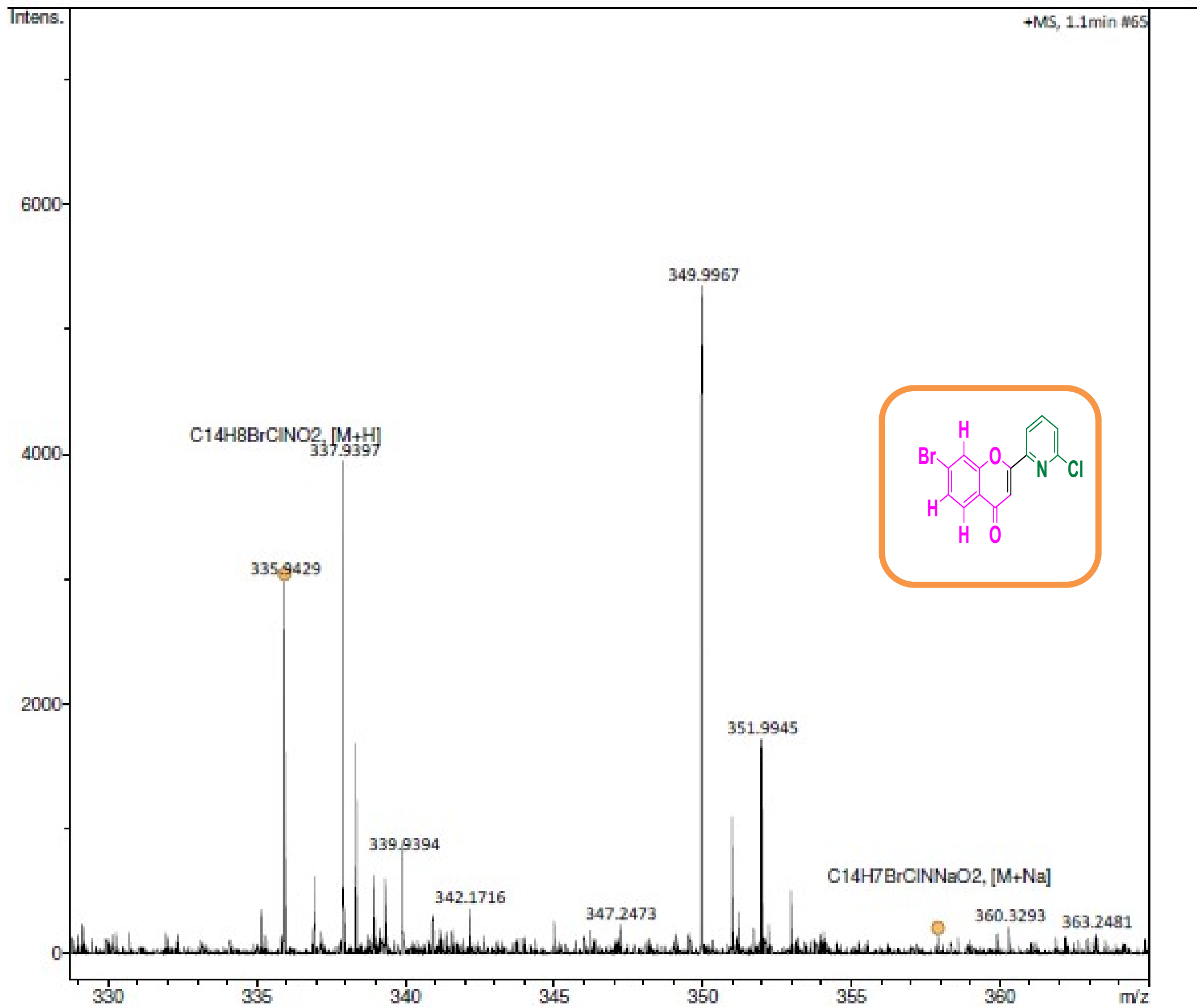

Intens.
+MS, 1.1min #65
6000
4000
2000
0
349.9967
C14H8BrClNO2, [M+H]
337.9397
335.9429
351.9945
339.9394
342.1716
347.2473
C14H7BrClNNaO2, [M+Na]
360.3293
363.2481
330
335
340
345
350
355
360
m/z
Br
H
O
N
Cl

S30 - 5c IR

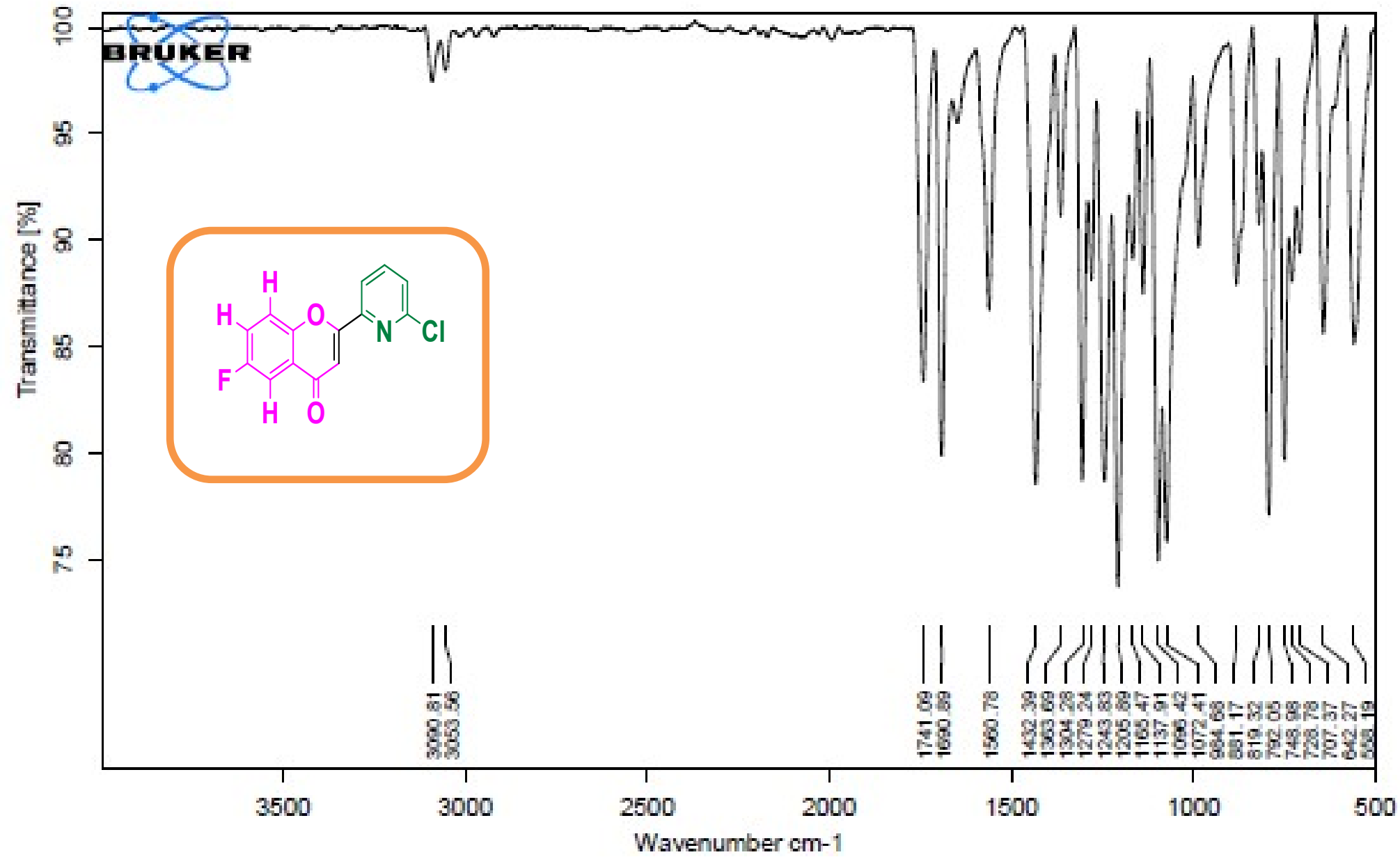

S31 - 5c ^{1}H NMR

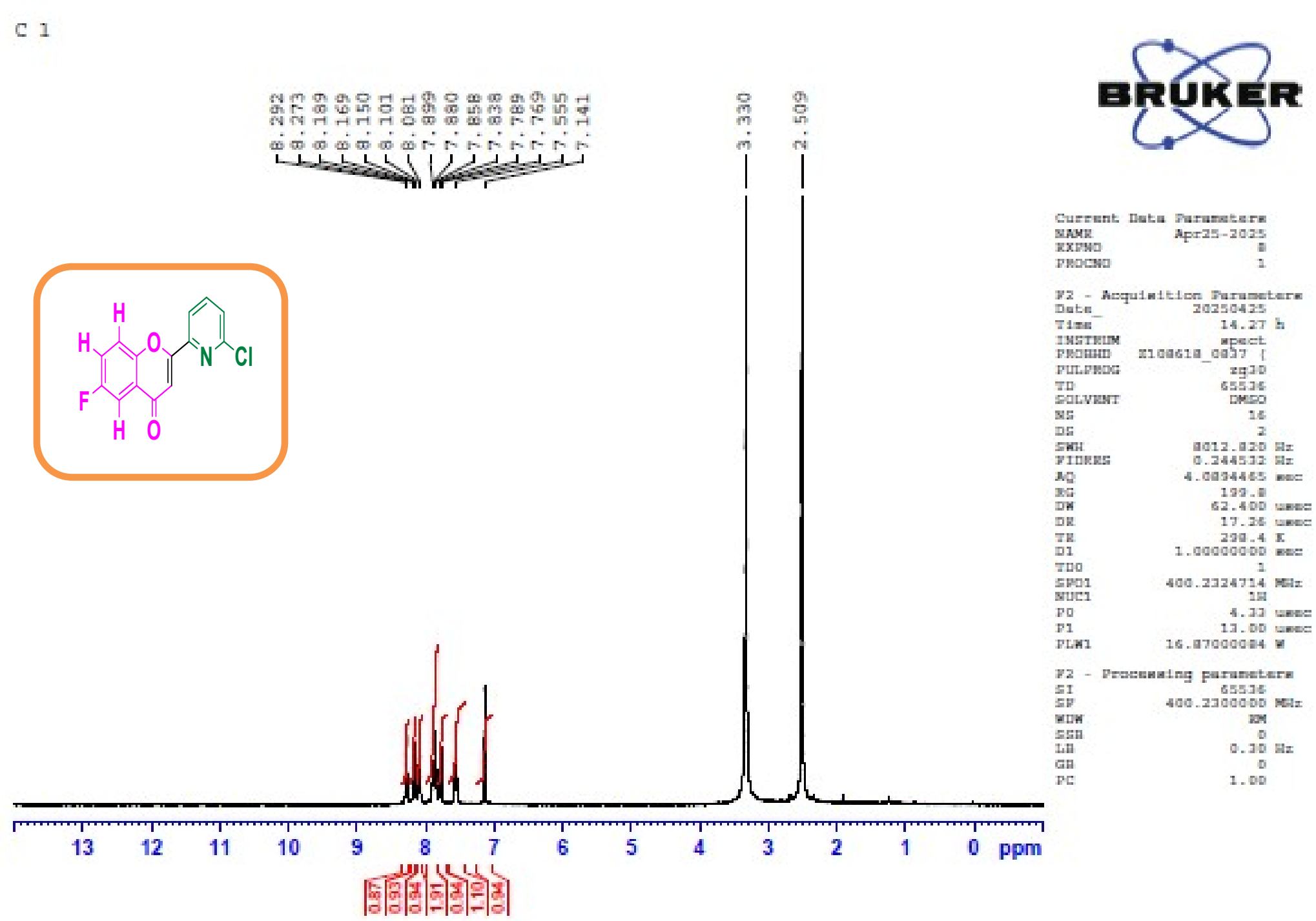

S32 - 5c ^{13}C NMR

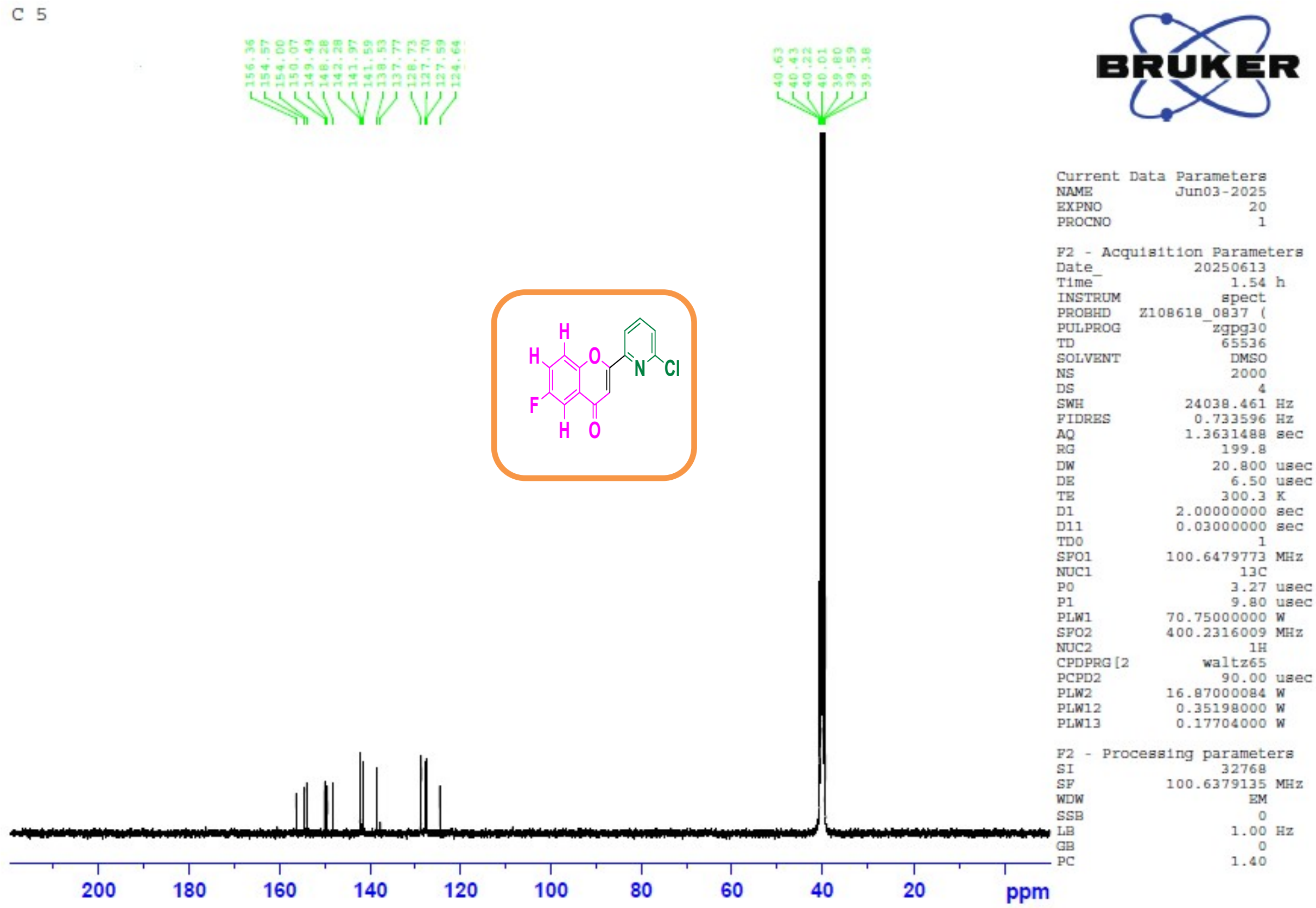

S33 - 5c HRMS

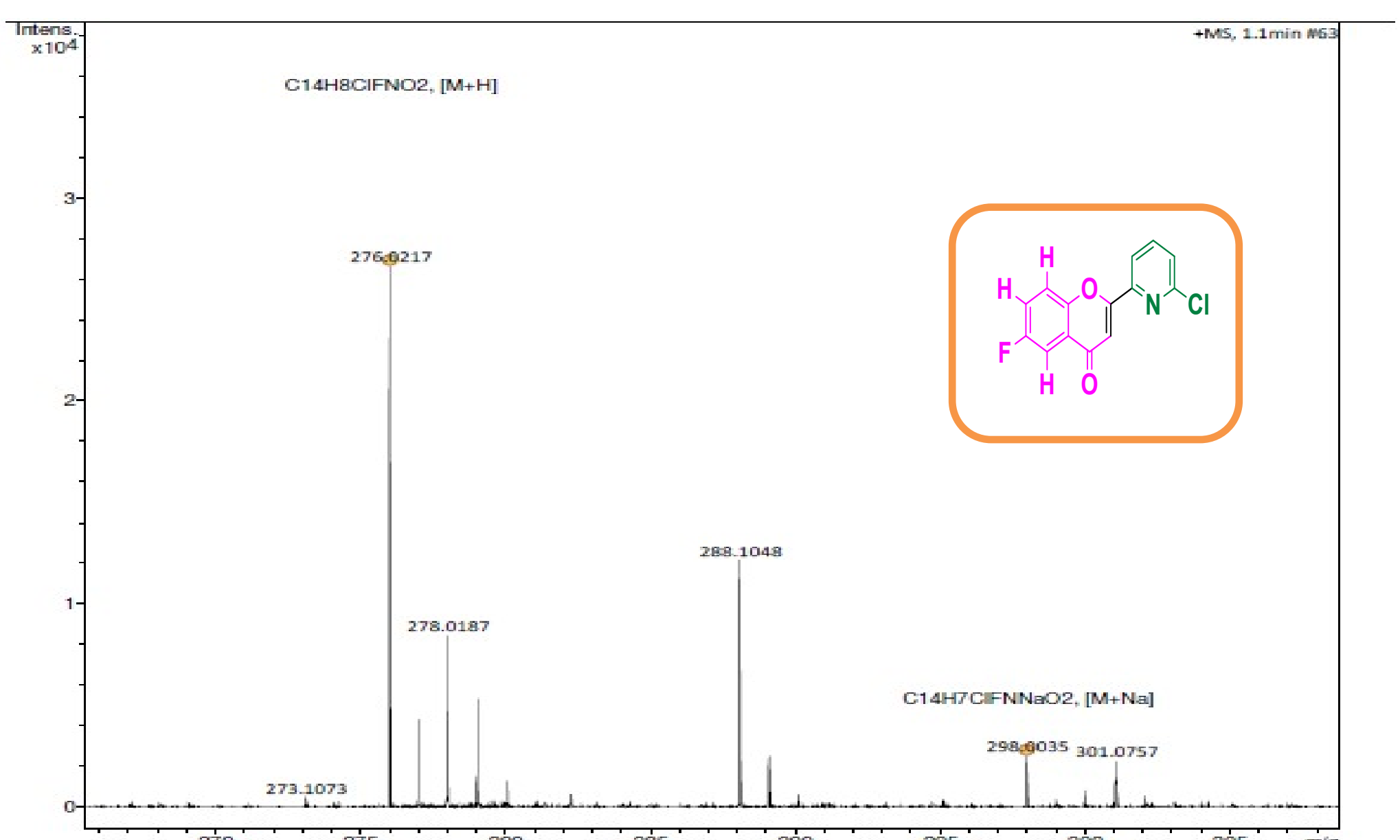

S34 - 6a IR

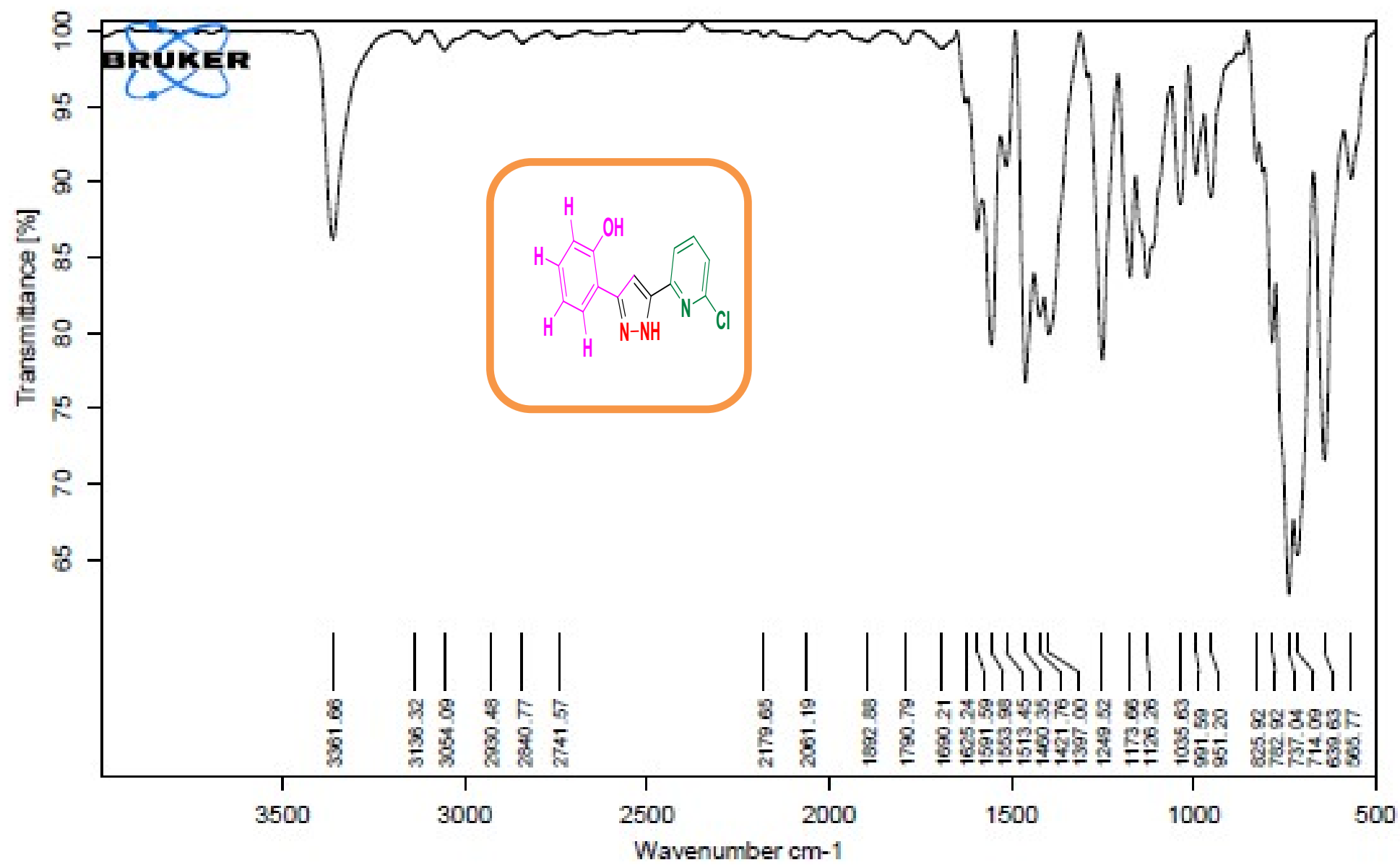

S35 - 6a ^{1}H NMR

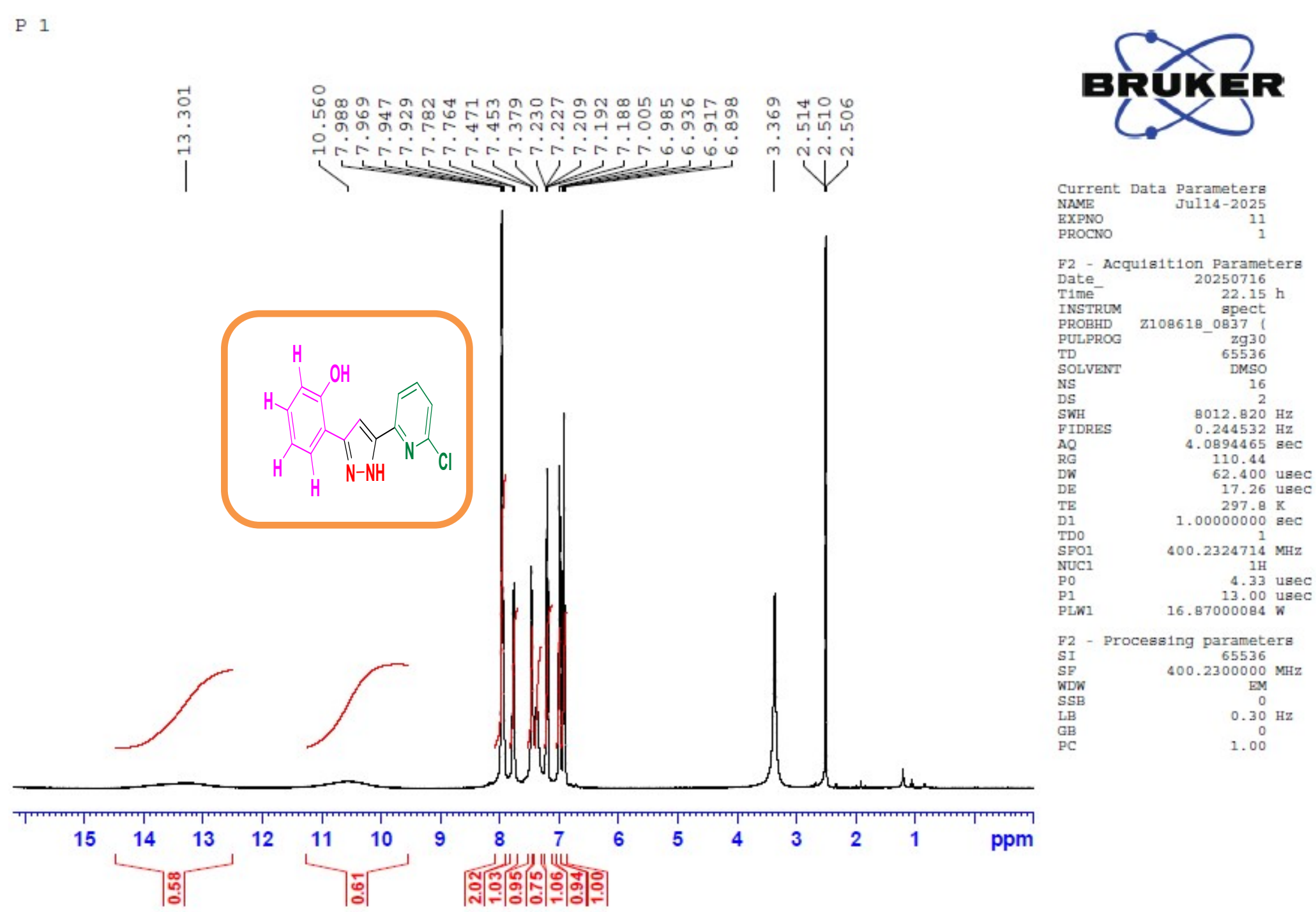

S36 - 6a ^{13}C NMR

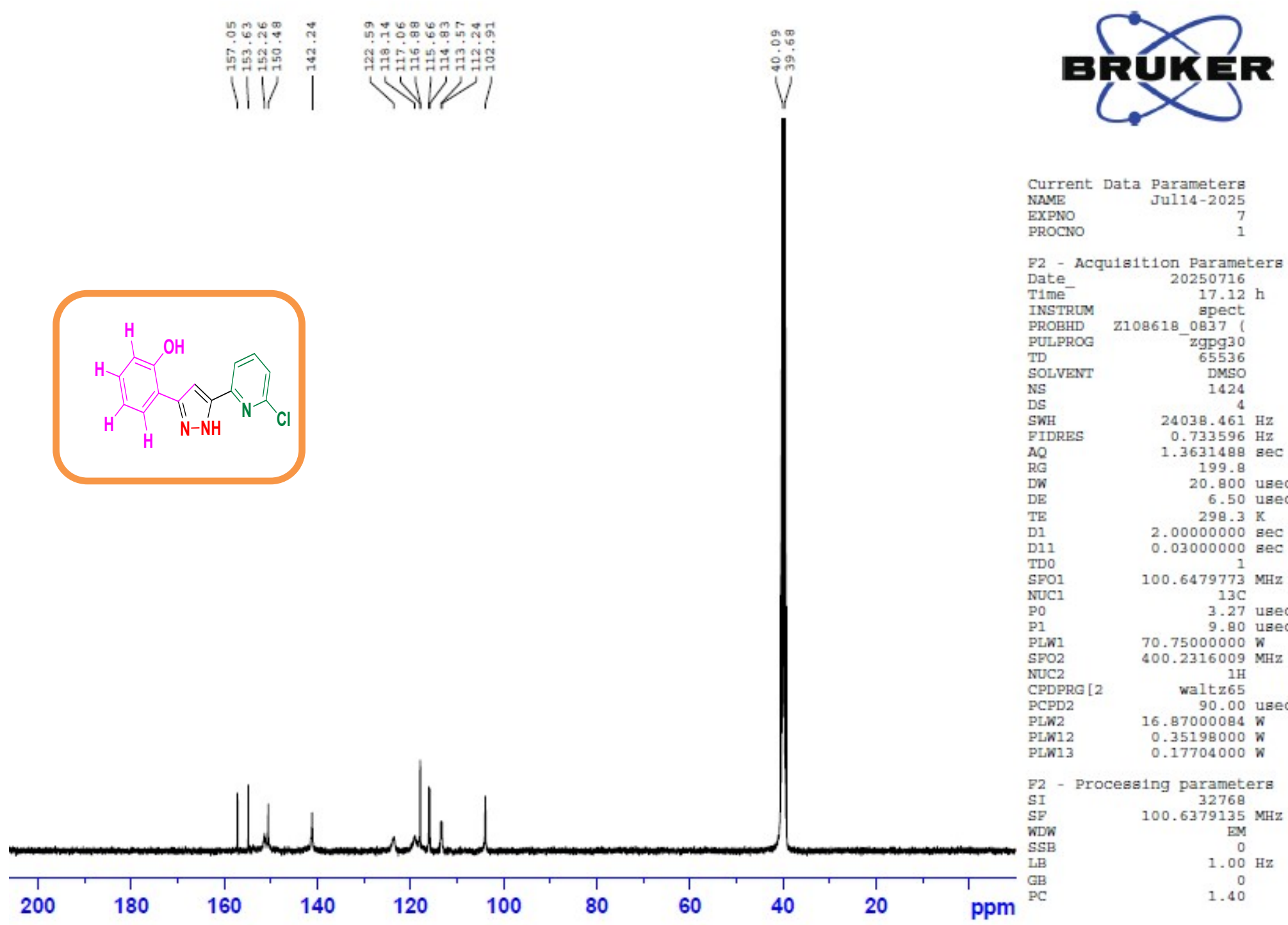

S37 - 6a HRMS

Acquisition Parameter

Source Type	ESI	Ion Polarity	Positive	Set Nebulizer	0.7 Bar
Focus	Active	Set Capillary	4000 V	Set Dry Heater	200 °C
Scan Begin	50 m/z	Set End Plate Offset	-500 V	Set Dry Gas	4.0 l/min
Scan End	1200 m/z	Set Charging Voltage	2000 V	Set Divert Valve	Source
		Set Corona	0 nA	Set APCI Heater	0 °C

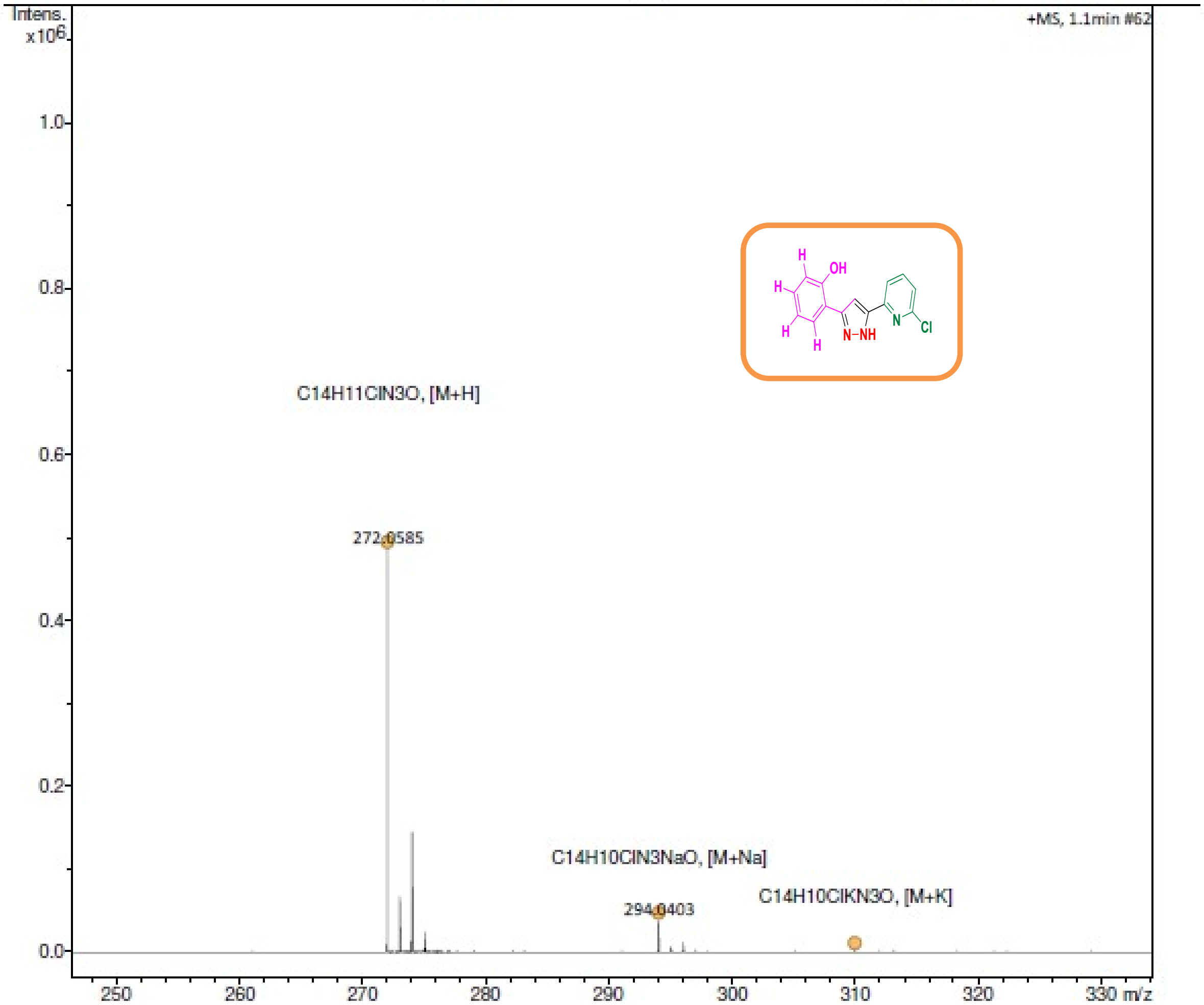

Meas. m/z	#	Ion Formula	Score	m/z	err [mDa]	err [ppm]	mSigma	rdb	e⁻ Conf	N-Rule	Adduct
272.058521	1	C14H11ClN3O	100.00	272.058516	-0.0	-0.0	22.2	10.5	even	ok	M+H
294.040310	1	C14H10ClN3NaO	100.00	294.040460	0.2	0.5	10.9	10.5	even	ok	M+Na

S38 - 6b IR

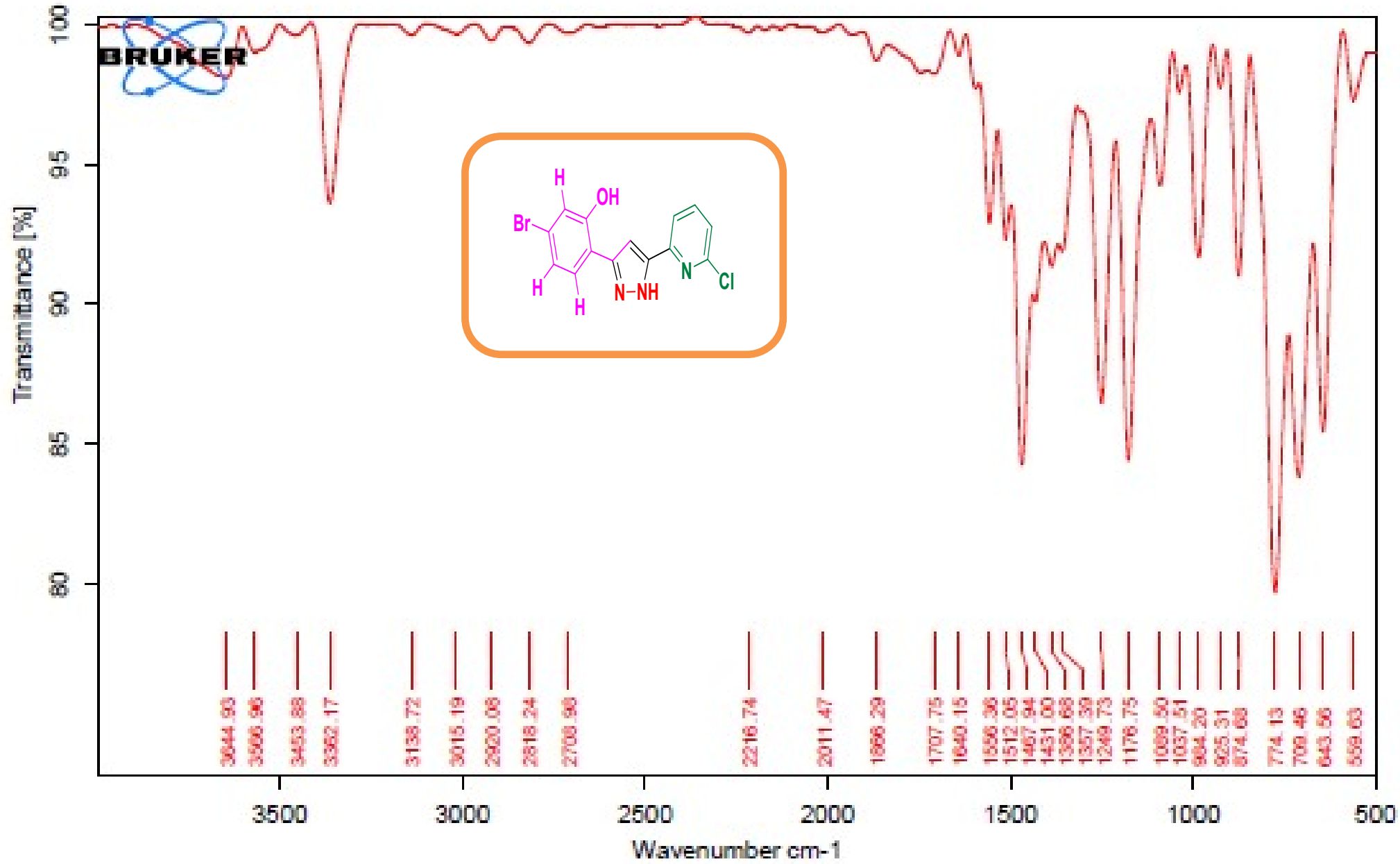

S39 - 6b ^{1}H NMR

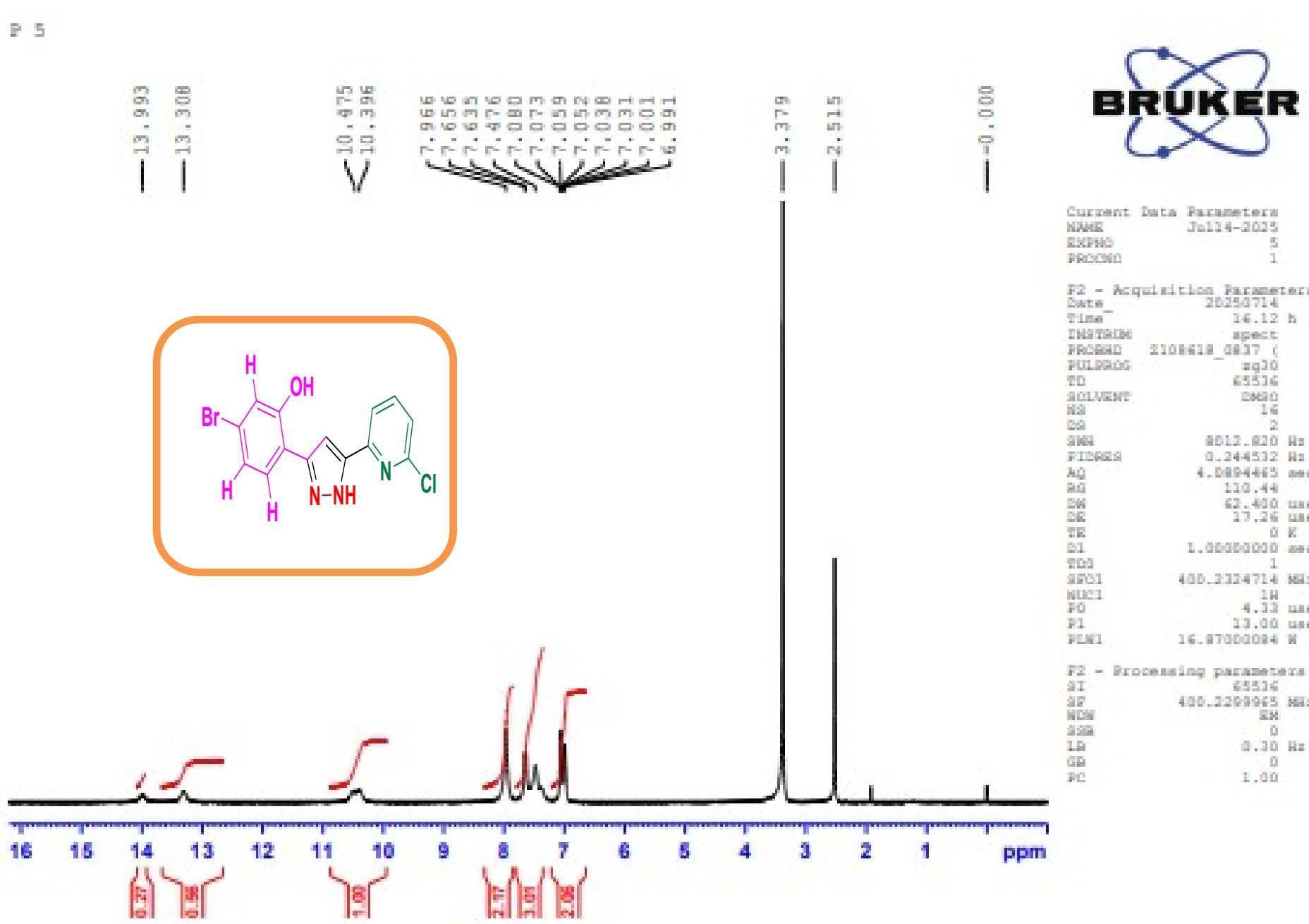

S40- 6b ^{13}C NMR

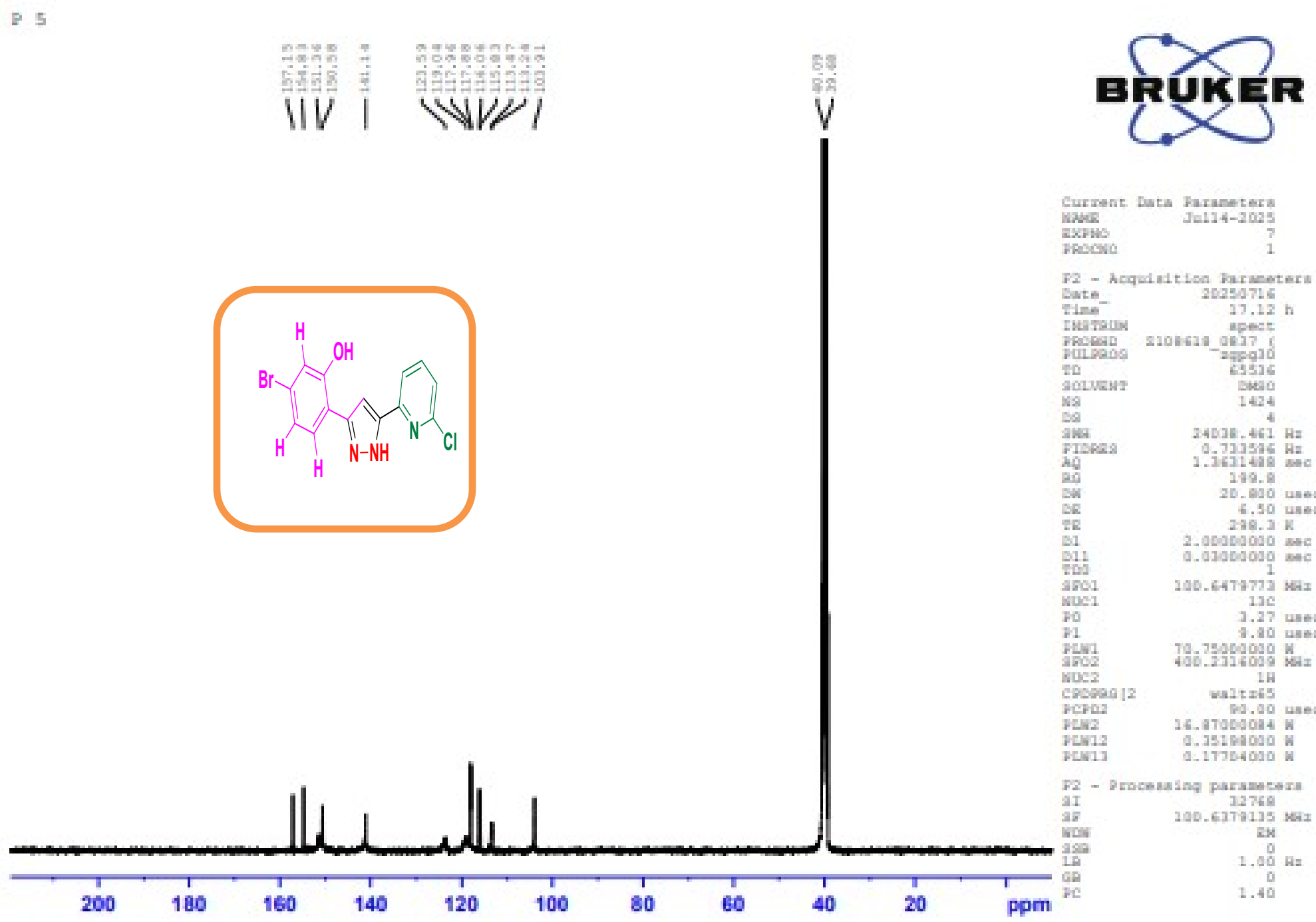

S41 - 6b HRMS

Acquisition Parameter

Source Type	ESI	Ion Polarity	Positive	Set Nebulizer	0.7 Bar
Focus	Active	Set Capillary	4000 V	Set Dry Heater	200 °C
Scan Begin	50 m/z	Set End Plate Offset	-500 V	Set Dry Gas	4.0 l/min
Scan End	1200 m/z	Set Charging Voltage	2000 V	Set Divert Valve	Source
		Set Corona	0 nA	Set APCI Heater	0 °C

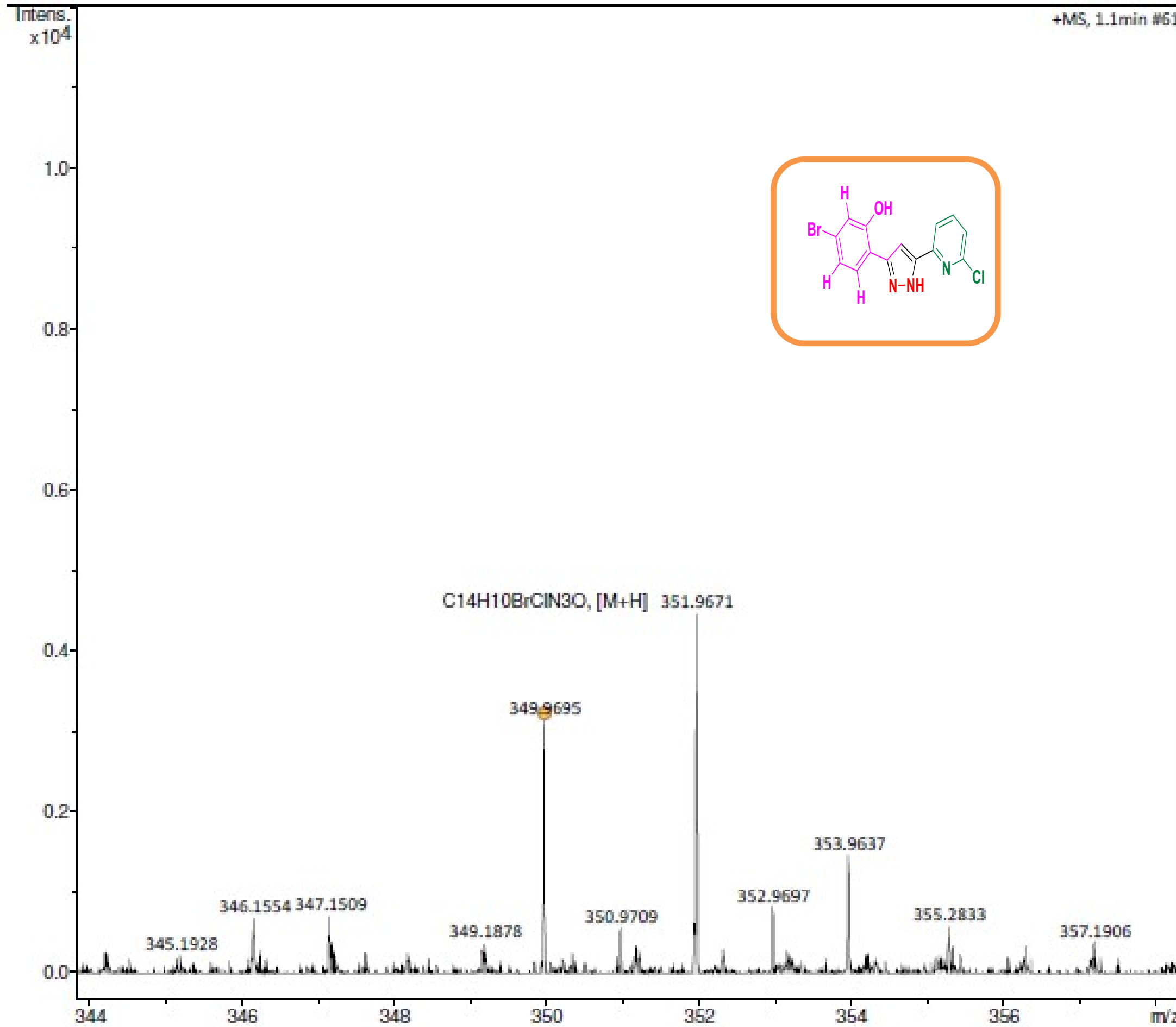

Meas. m/z	#	Ion Formula	Score	m/z	err [mDa]	err [ppm]	mSigma	rdb	e⁻ Conf	N-Rule	Adduct
349.969538	1	C14H10BrClN3O	100.00	349.969028	-0.5	-1.5	43.9	10.5	even	ok	M+H

S42 - 6c IR

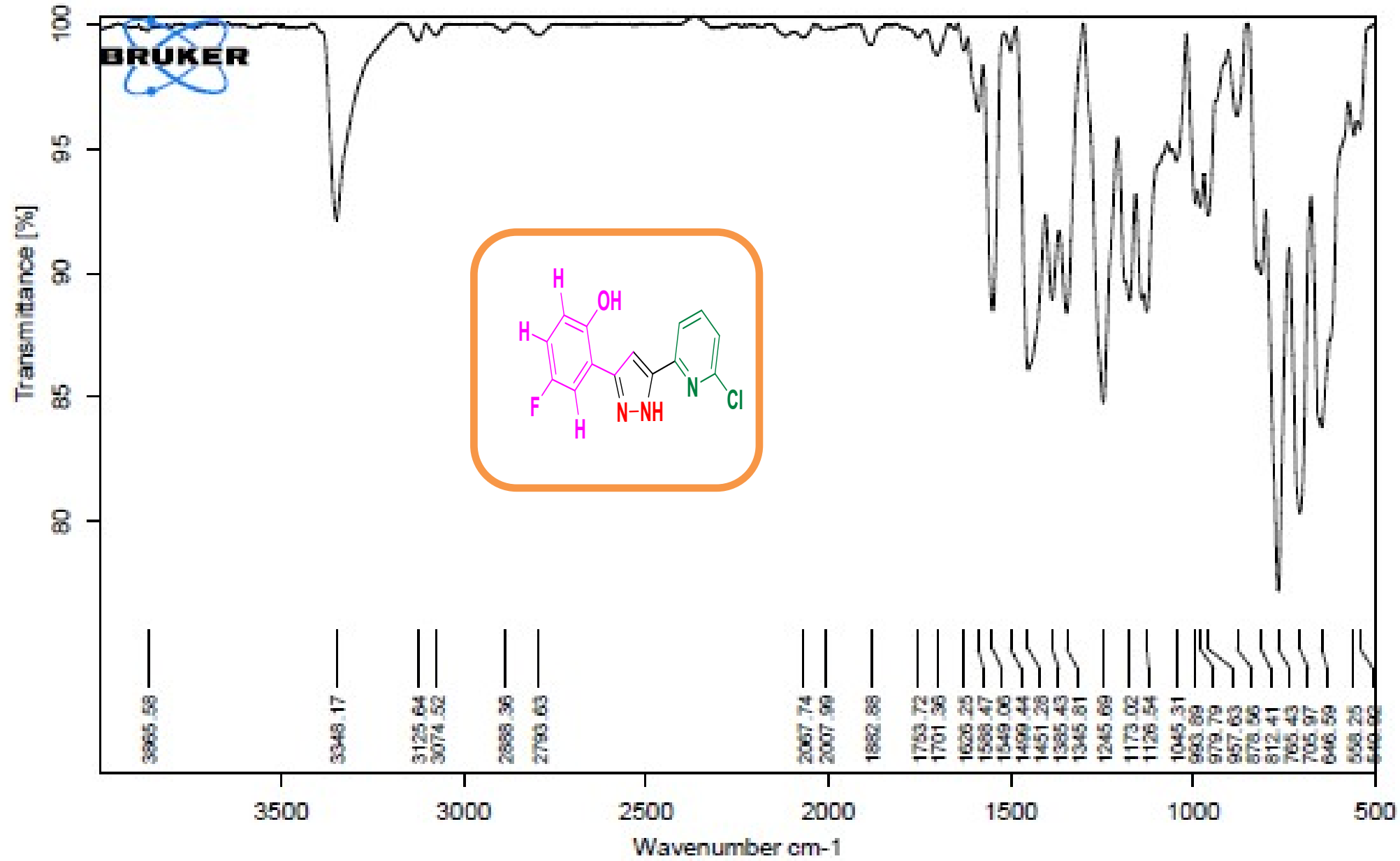

S43 - 6c ^{1}H NMR

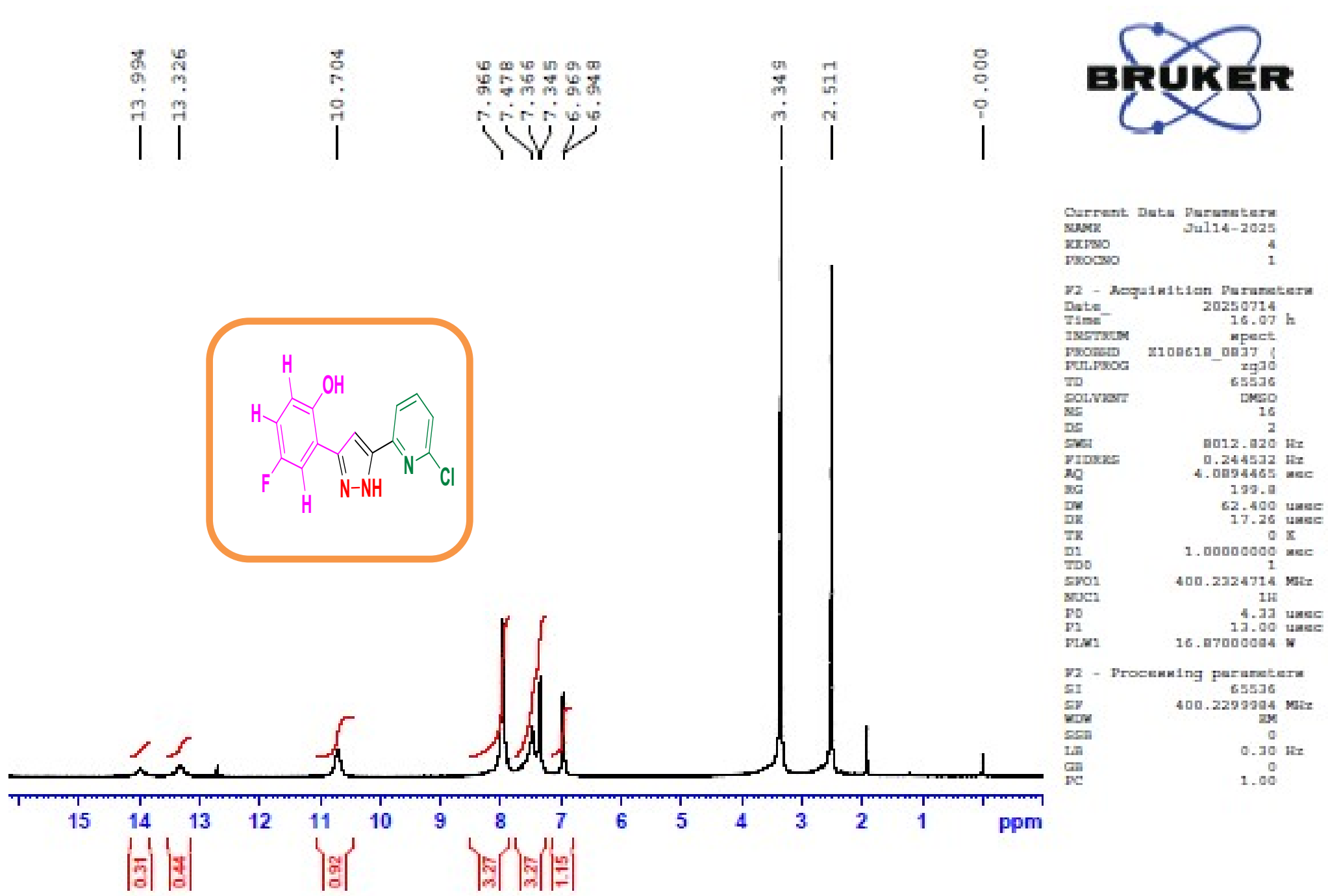

S44 - 6c ^{13}C NMR

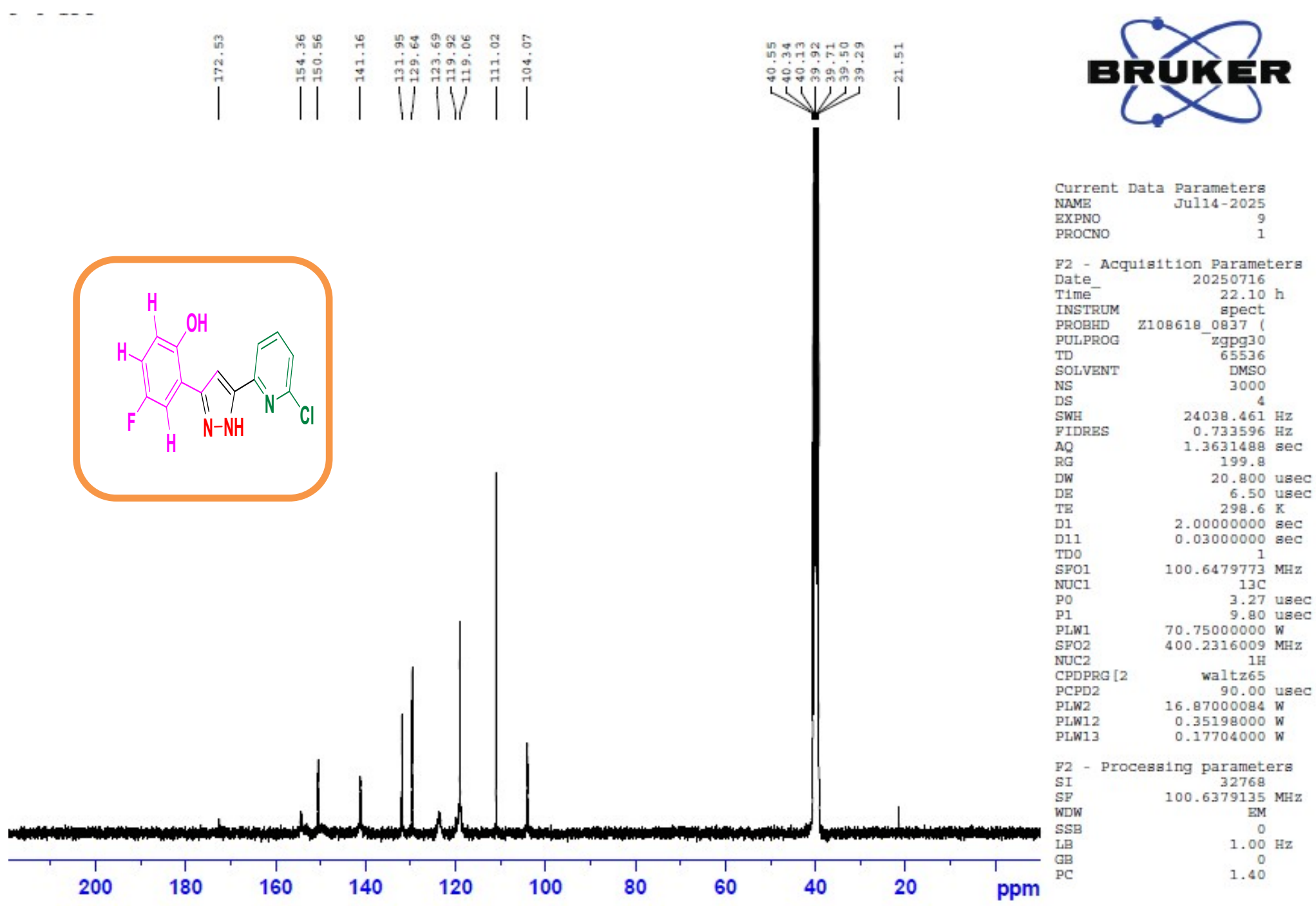

S45 - 6c HRMS

Savitribai Phule Pune University - Central Instrumentation Facility

Analysis Info

Analysis Name	PM D:\Data\2025\SEPT\SPPU COLLEGE\MODERN COLLEGE,GANESHKHIND\ARUNA SHINDE\3 C_RD1_01_10797.d	Acquisition Date	05/05/2026 5:11:08
Method	dlc_50-1200mz_pos_mode_7min_0.2ml_jun2025.m	Analyst :	Nitin S. Kadam CIF
Sample Name	6C	Instrument	impact HD 1819696.00184

Acquisition Parameter

Source Type	ESI	Ion Polarity	Positive	Set Nebulizer	0.7 Bar
Focus	Active	Set Capillary	4000 V	Set Dry Heater	200 °C
Scan Begin	50 m/z	Set End Plate Offset	-500 V	Set Dry Gas	4.0 l/min
Scan End	1200 m/z	Set Charging Voltage	2000 V	Set Divert Valve	Source
		Set Corona	0 nA	Set APCI Heater	0 °C

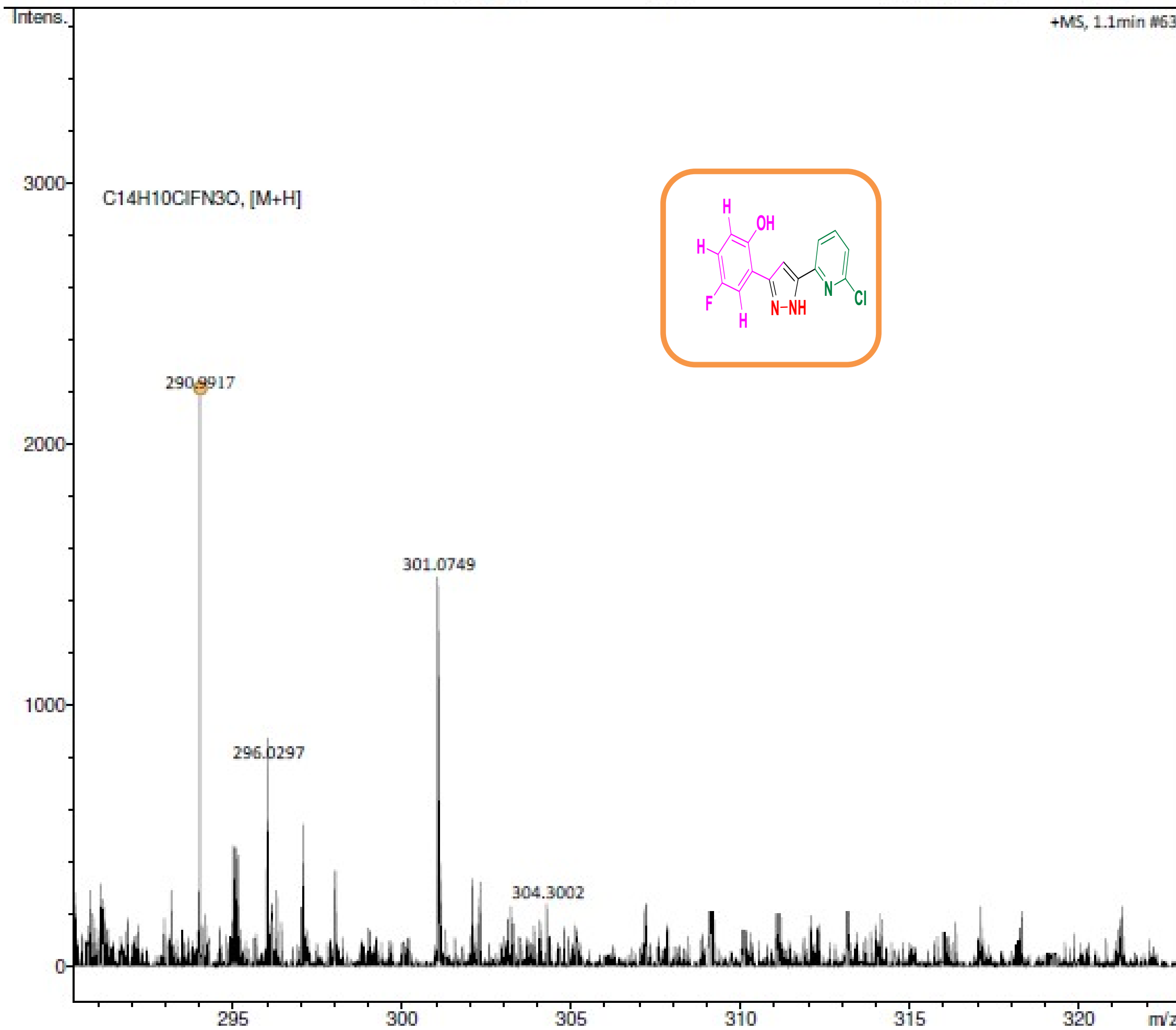

Meas. m/z	#	Ion Formula	Score	m/z	err [mDa]	err [ppm]	mSigma	rdb	e⁻ Conf	N-Rule	Adduct
290.991711	1	C14H10ClFN3O	100.00	290.991711	-1.5	-4.9	38.1	9.5	even	ok	M+H

Figure 1: Antimicrobial activity of synthesized compounds (3a-6c): (A) agar well diffusion plates showing inhibition zones (mm)

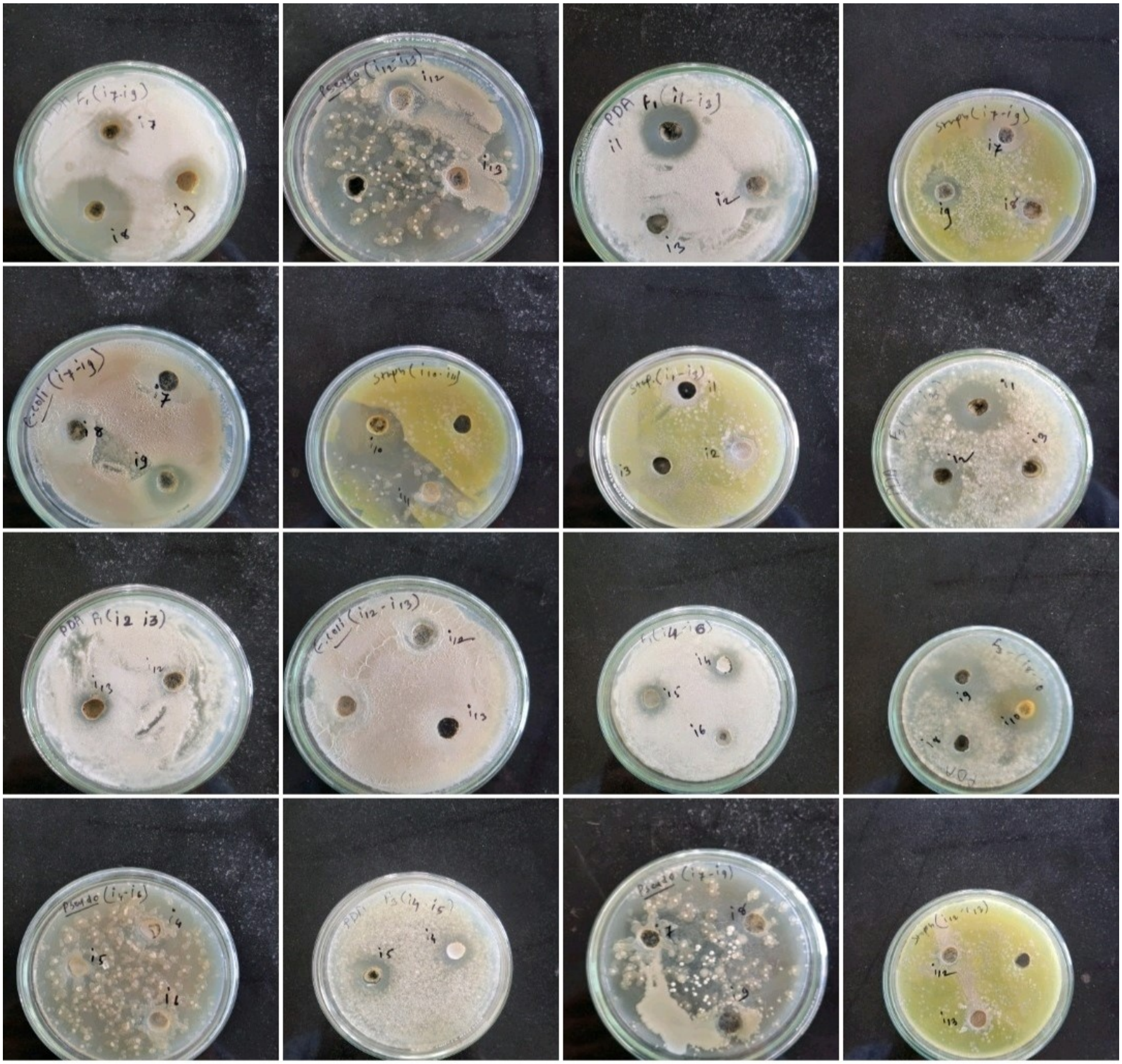

Figure 2. Bar graph illustrating zone of inhibition (mm) against tested microorganisms.

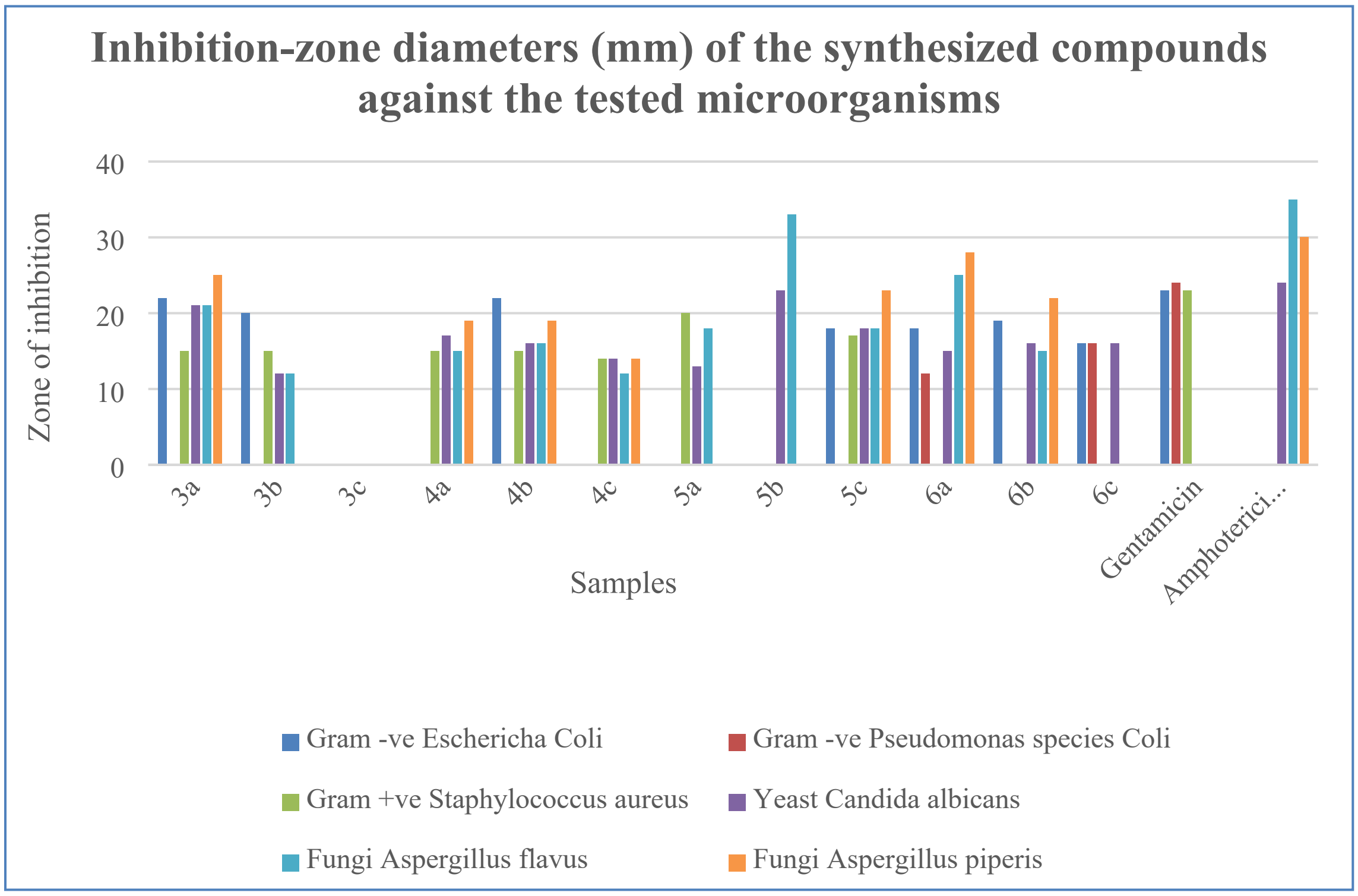

2.3. Agar well diffusion method:

The microbial strains were sub cultured on the appropriate agar slants for 24 to 48 hours at 4 °C before testing. To prepare the test solutions, the synthesised compounds (**3a-6c**) were dried and dissolved in dimethyl sulfoxide (DMSO). To prevent DMSO from interfering with the antibacterial action, the final concentration in the test wells was kept between 1% and 2% (v/v). A 0.22 µm membrane was used to filter-sterilise the stock solutions, which were then aseptically prepared at 100 mg/mL (or at their maximum solubility), vortexed to ensure complete dissolution, and stored at 4 °C for no more than a week.

Mueller-Hinton agar (MHA) was used as the selective medium for bacterial cultures, while potato dextrose agar (PDA) was used for fungal species. To standardise the inoculum, three to five well-isolated colonies from freshly prepared (18-24 hours) cultures of *Escherichia coli*, *Pseudomonas* sp., and *Staphylococcus aureus* were suspended in sterile 0.85% saline. A 24-48 hrs. PDA culture of *Candida albicans* was used to prepare the yeast inoculum in sterile saline. Fungal spore suspensions of *Aspergillus flavus* and *Aspergillus piperis* were similarly prepared from 5-7 day old PDA cultures. The agar well diffusion method was utilised to assess the antibacterial and

antifungal properties. A sterilised glass spreader was used to evenly distribute the microbial inocula across the agar surface. The plates were lid and left to air-dry for three to five minutes following inoculation. A sterile borer was then used to aseptically punch 6 mm-diameter wells, ensuring at least 20 mm between each well. After carefully removing the agar plugs, 50 µL of each test solution was added to each well.

The positive controls for bacterial strains were gentamicin (10 µg/50 µL), whereas those for filamentous fungi and yeast were amphotericin B (10 µg/50 µL). Use of DMSO (≤1-2% v/v) as the negative control was employed. To ensure sufficient sample diffusion, the plates were left at room temperature with laminar airflow for 30 to 45 minutes after the wells were loaded.

Until clearly defined inhibitory zones formed, the infected plates were incubated at 35 ± 2 °C for 16-20 hours for bacterial strains, 30-35 °C for 24-48 hours for Candida albicans, and 25-28 °C for 48-72 hours for Aspergillus species. The diameters of the inhibitory zones (mm), including the 6 mm well diameter, were measured to evaluate antimicrobial activity. A digital vernier calliper or transparent ruler was used to measure the bottom of each Petri dish. Three measurements were taken around each zone at approximately 60°C intervals to ensure accuracy, and the mean value was reported to reduce observational error.
